# The interplay between species and locations shapes vector fleas microbial communities in plague foci: pathogens rather than symbionts?

**DOI:** 10.3389/fcimb.2025.1568103

**Published:** 2025-05-20

**Authors:** Shahin Seidi, Abbasali Raz, Naseh Maleki-Ravasan, Esmaeil Forouzan, Fateh Karimian, Florent Sebbane, Aria Sohrabi, Saber Esmaeili, Ehsan Mostafavi

**Affiliations:** ^1^ Department of Epidemiology and Biostatics, Research Centre for Emerging and Reemerging Infectious Diseases, Pasteur Institute of Iran, Tehran, Iran; ^2^ National Reference Laboratory for Plague, Tularemia and Q Fever, Research Centre for Emerging and Reemerging Infectious Diseases, Pasteur Institute of Iran, Kabudar Ahang, Hamadan, Iran; ^3^ Malaria and Vector Research Group, Biotechnology Research Center, Pasteur Institute of Iran, Tehran, Iran; ^4^ Department of Parasitology, Pasteur Institute of Iran, Tehran, Iran; ^5^ Razi Vaccine and Serum Research Institute (RVSRI), Agricultural Research, Education and Extension Organization (AREEO), Karaj, Iran; ^6^ University Lille, CNRS, INSERM, CHU Lille, Institut Pasteur de Lille, U1019-UMR9017-CIIL-Centre d’Infection et d’Immunité de Lille, Lille, France

**Keywords:** microbiome, *Yersinia pestis*, *Bartonella*, *Wolbachia*, *Cardinium*, *Rickettsia*, flea-borne diseases

## Abstract

Besides causing allergies from their bites, fleas transmit the most notorious zoonotic pathogen, *Yersinia pestis*. They also harbor commensal bacteria in their guts. Here, the microbial communities of fleas were characterized using *16S rRNA* Next-Generation Sequencing to understand microbial interactions and functions in areas with historical plague-outbreaks in Iran with the ultimate idea of ​​managing flea-borne diseases. *Meriones persicus*, *Xenopsylla buxtoni* and *Bartonella* spp. were identified as the dominant host, vector and bacterium, respectively. Six bacteria *Bartonella*, *Sphingomonas*, *Wolbachia*, *Cardinium*, *Rickettsia* and *Ralstonia* were identified as the most abundant genera in the microbiome of five flea species. More detailed surveys revealed substantial intrageneric variations (e.g. nine phylotypes for *Bartonella*) and the diverse nature (from biofilm-forming human pathogens to insect reproductive manipulators, and environmental microbes) for the bacteria studied. The fleas microbiome is largely affected by species and to a lesser extent by location, and circulates by both horizontal and vertical transmissions. The prevalence of *Bartonella* spp. infection in fleas highlights the potential to explore One Health approaches, particularly in addressing travel-related and zoonotic disease risks. Environmental drivers—such as climate change, habitat alteration, and host dynamics—shape flea microbiomes and influence disease risk, while concerns about antimicrobial resistance further complicate control efforts. Our findings advocate for coordinated strategies that combine public health education, ecological monitoring, and global collaboration to sustainably manage flea-borne diseases.

## 1 Introduction

Fleas (Siphonaptera) are small, wingless, and highly specialized blood-sucking ectoparasites of mammals and birds. Among the 2,718 known flea species (Hastriter et al., 2023), six species of *Pulex irritans*, *Ctenocephalides felis felis*, *Xenopsylla cheopis*, *Nosopsyllus fasciatus*, *Echidnophaga gallinacea*, and *Tunga penetrans* are synanthropic (Bitam et al., 2010). Fleas affect directly and indirectly human and livestock health, as their bites can cause significant discomfort, skin allergies, and secondary infections. More importantly, fleas are vectors and intermediate hosts of serious zoonotic pathogens (Sharif, 1938; Dobler and Pfeffer, 2011; Maleki-Ravasan et al., 2017; Sanchez et al., 2018; Boucheikhchoukh et al., 2022). Remarkably, they transmit the plague bacillus (*Yersinia pestis*), the agent of murine typhus (*Rickettsia typhi*), and the agent of cat scratch disease (*Bartonella henselae*) (Comer et al., 2001; Billeter et al., 2008; Seidi et al., 2024). Fleas have also been proposed as vectors of prevalent zoonotic foodborne diseases such as listeriosis, brucellosis, and salmonellosis (Lane and Crosskey, 2012). Fleas transmit pathogenic microorganisms via their feces (e.g. *R. typhi* and *B. henselae*), their soiled mouthparts (e.g. viral pathogens), or after regurgitation of gut contents (e.g. *Y. pestis*) or saliva (e.g. *R. felis*) (Eisen and Gage, 2012). Lastly, unintentional flea ingestion by hosts during grooming can also lead to flea-borne transmission of the pathogenic agents (Lane and Crosskey, 2012; Eisen et al., 2021).

There have been three human plague pandemics that considerably affected human civilizations (Ditchburn and Hodgkins, 2019). During the 19^th^ and 20^th^ centuries, 49 plague outbreaks were documented in Iran, with approximately one-third of the outbreaks occurring in the northwest of the country (Hashemi Shahraki et al., 2016). Among them, three outbursts in Kurdistan and its neighboring areas, Bukan and Sarab, are conspicuous (Hashemi Shahraki et al., 2016). In Iran, *Meriones* species (*M. persicus*, *M. tristrami*, *M. vinogradovi*, and *M. libycus*) and *Mesocricetus auratus* are known as hosts, while fleas *P. irritans*, *X. cheopis*, *X. astia*, *X. buxtoni*, *X. conformis*, *N. fasciatus*, and *N. iranus iranus* are recognized as vectors of plague infection (Seyf, 1989; Baltazard et al., 1952; Baltazard and Bahmanyar, 1960a; Baltazard and Bahmanyar, 1960b; Karimi et al., 1977; Karimi, 1980).

Only a limited number of bacteria act as pathogens, while many other bacteria are either harmless, beneficial, or even essential for the insect host (Douglas, 2015). These microbes play essential roles in meeting nutritional needs, reproduction/development, immune system modulation, pathogen colonization, and pathogen transmission, i.e. vector competence (Vogel and Coon, 2020; Sonenshine and Stewart, 2021). However, their role outside the vector’s gut and during transmission to the vertebrate host is controversial (Amni et al., 2023; Maleki-Ravasan et al., 2024). These microbes are categorized on a spectrum including specialized symbionts that are vertically transferred from mother to offspring, to optionally symbiotic and free-living microbes that are obtained from the surrounding environment or food (Vogel and Coon, 2020; Chamankar et al., 2023). These features make the microbiota—microscopic life forms inhabiting specific environments within insect vectors— an arsenal to combat the transmission of pathogens by insect vectors in the context of strategies such as *Wolbachia*-mediated biocontrol or paratransgenesis (Dehghan et al., 2017; Saldaña et al., 2017; Dehghan et al., 2022).

Despite the economic and medical importance of fleas, the flea microbiome has not yet been fully investigated. Several studies have examined the microbiomes of *Echidnophaga ambulans*, *Ceratophilus idius*, *Oropsylla*, and *Rhadinopylla* species (Jones et al., 2012; Lawrence et al., 2015; Li et al., 2018; Sridhar et al., 2022). Most of these studies have focused on the microbiome of the flea *C. felis*, exploring the effect of environmental conditions, infection, and host taxonomy on the gut microbiota composition (Pornwiroon et al., 2007; Lawrence et al., 2015; Manvell et al., 2022; Matthee et al., 2023; Moore et al., 2023; Wu et al., 2023). These suverys have identified *Rickettsia*, *Bartonella*, and *Wolbachia* as the predominant genera in the cat flea microbiome (Vasconcelos et al., 2018). However, there is a lack of comprehensive research to the nature of flea bacteria and compare their microbiome in endemic and non-endemic areas of zoonotic diseases. Comparing the microbiomes of fleas from endemic and non-endemic regions provides critical insights into the ecological and environmental drivers of flea-borne disease dynamics. Endemic areas, often characterized by higher densities of reservoir hosts and vectors, serve as hotspots for zoonotic pathogens like *Y. pestis* and *Bartonella* spp., whereas non-endemic areas offer a contrasting backdrop with potentially lower pathogen prevalence and different microbial patterns. This comparison helps identify key bacterial taxa linked to disease risk, assess microbial diversity and transmission modes (horizontal vs. vertical), and evaluate how environmental factors such as climate and habitat influence pathogen persistence. Ultimately, this approach supports targeted surveillance, public health interventions, and One Health strategies to prevent disease emergence and manage travel-related risks. With this background, two main questions are addressed in this research: (1) what is the structure and diversity of bacterial communities in the fleas of endemic and non-endemic foci of plague?; (2) Can the flea microbiota be categorized into pathogenic and symbiotic groups to support the development of targeted control strategies, such as disease risk prediction and microbiome-based vector control (e.g., through the use of symbionts for biocontrol)? To achieve this goal, the structure and diversity of bacterial communities in fleas were characterized in terms of species, gender, and geographic regions. The findings of the present study would be helpful not only in understanding the potential zoonotic flea-borne diseases but also in unraveling the symbiotic species from the pathogen ones to use in safe vector control strategies.

## 2 Materials and methods

### 2.1 Study areas, collection, and processing of specimens

**Figure 1:**
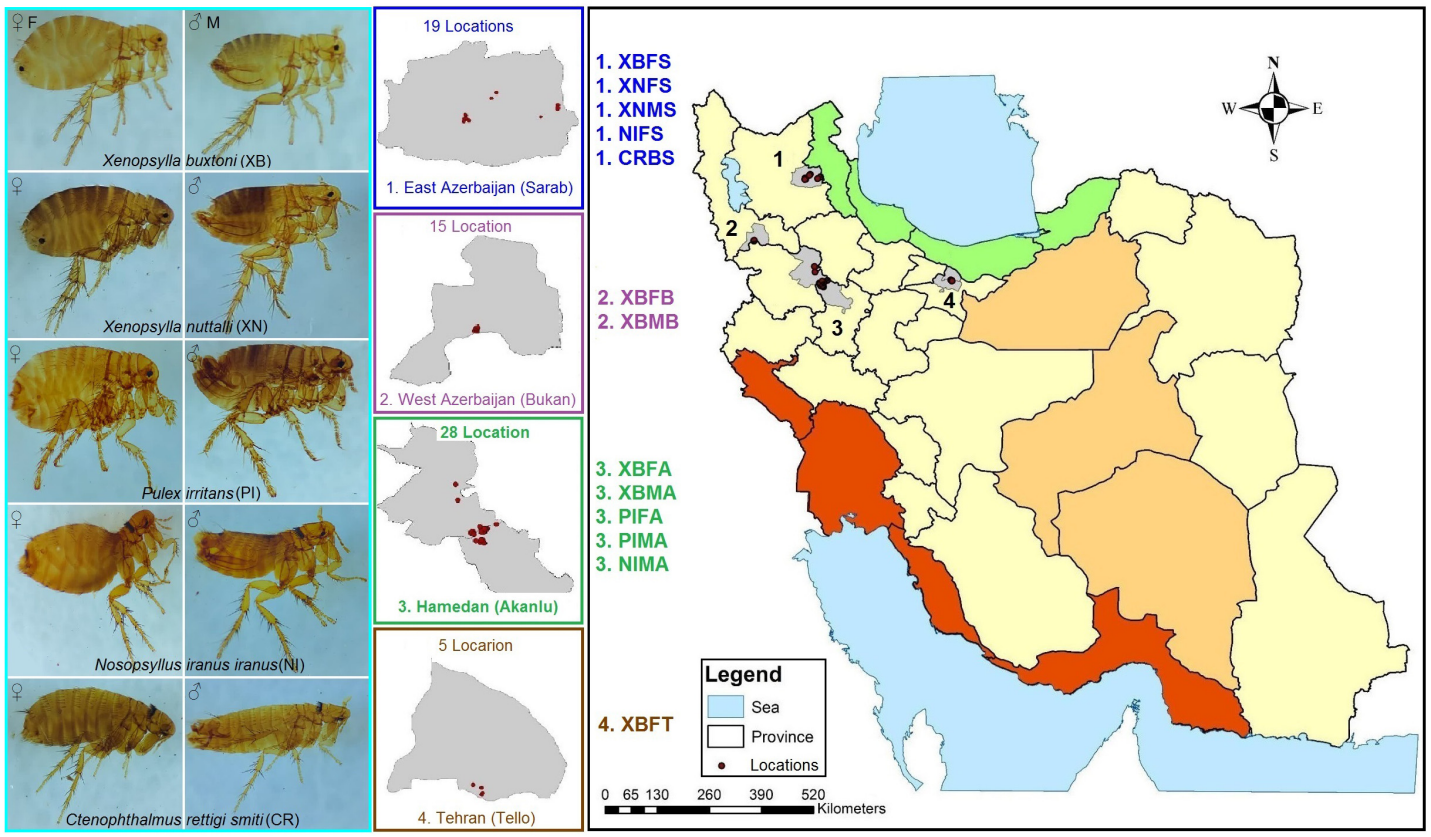
Map of study areas showing locations with (East and West Azerbaijan and Hamadan Provinces) or without (Tehran Province) historical occurrence of plague in Iran. The coloring of the provinces is based on the major climate zones of Iran. The coding of the specimen names was generated based on the species, gender and place of collection according to the pattern presented in the map.

This study was conducted in Hamadan (Akanlu), West Azerbaijan (Bukan), and East Azerbaijan (Sarab) Provinces, located in the west and northwest of Iran, where plague outbreaks and circulation of *Y. pestis* have been reported in the last hundred years, and one region in the northeast of Tehran (Tello) Province at the center of Iran, where there have been no reports of plague outbreaks so far (**Figure 1**). The samples were collected from 67 locations in these areas from April to September 2022. Sampling and interaction with vertebrates and higher invertebrates were carried out in accordance with national and international (IR.PII.REC.1400.047 and 1986; 86/609/EEC) animal study guidelines.

Rodents were captured in wooden live catch traps. The traps baited with dates were set in the early evening, and the closed traps containing the captured animals were collected the next morning. The rodent trapping continued for three days. Geographic coordinates of sampling points were recorded using the Global Positioning System (GPS). The captured rodents were identified using key morphological characteristics (Kryštufek and Vohralík, 2001; Darvish et al., 2014). Also, a dead red fox that had collided with passing vehicles on Akanlu Road was selected for sampling.

Fleas were removed from the hosts by blowing the hairs of the captured animals into a tray of clean water. This process caused the fleas to float in the water, making them unable to move and jump. The fleas were immediately gathered from the water and transferred to the research laboratory in sterile Eppendorf tubes containing 70% alcohol within a cold chain. Fleas collected from each host and location, comprising 146 samples and 13 pools, were divided into two groups. The first group was used for morphological identification of fleas by mounting them on slides in Pouri’s solution, and the second group was used to examine bacterial symbionts and pathogens using the next-generation sequencing (NGS) technique. Each slide was assigned a unique code. Fleas species were morphologically identified using microscopic (Olympus SZ40, Olympus Corporation, Tokyo, Japan) examination and available identification keys (Asmar et al., 1979), along with other references cited in (Maleki-Ravasan et al., 2017). Following the taxonomic identification of the first group, the samples from the second group were treated twice with 70% ethanol and centrifugated (Eppendorf 5415R centrifuge, Eppendorf AG, Hamburg, Germany) to remove any adhering microbes and foreign particles from the exoskeleton. The samples were then stored in ethanol at -20°C.

### 2.2 DNA extraction, PCR, and amplicon sequencing

Fleas of the same genus, species, sex, and collection site—isolated from a single host—were pooled. Each pool included 12 specimens, except for the *Ctenophthalmus rettigi smiti* pool, which contained only two specimens. All samples were subjected to genomic DNA extraction. Initially, the alcohol-free fleas’ bodies were entirely ground inside 1.5 mL microtubes. Total DNA was then extracted from the specimens using the QIAamp DNA Mini Kit (ID: 51304, Qiagen, Germany) following the manufacturer’s instructions for extracting DNA from tissues. Also, RNA contamination in the specimens was eliminated by adding 1 μl of RNase A (10 mg/ml; Thermo Fisher Scientific, Netherlands) to each 100 μl of DNA dissolved in the elution buffer and by incubating at 37°C for 1 h. The primers 341F (5′-CCTAYGGGRBGCASCAG-3′) and 806R (5′-GGACTACNNGGGTATCTAAT-3′) were utilized to amplify approximately 460 bp of the *16S rRNA* gene flanking the V3–V4 hypervariable regions of both bacteria and archaea (Yu et al., 2005). Details on negative controls and contamination prevention were considered as mentioned in the previous work (Chamankar et al., 2023). PCR was carried out for 35 cycles of amplification involving 5 s at 98°C, 20 s at 56°C, and 20 s at 72°C using Titanium Taq DNA Polymerase (Clontech, Takara, Japan). After electrophoresis and purification, the successful amplicons were submitted to the BGI group in China for sequencing *16S rRNA*-NGS using the Illumina HiSeq platform, with the minimum error rates <1%. The metagenomic sequencing libraries were developed in line with our prior protocol (Chamankar et al., 2023). Raw sequences have been deposited in the NCBI Sequence Read Archive (SRA) under the BioProject accession number PRJNA995036.

### 2.3 Bioinformatic and statistical analysis of microbiome data

The process was conducted with the analysis of raw sequences following the paired end-data instructions using QIIME2 v. 2018.1 (Quantitative Insights Into Microbial Ecology 2) (Bolyen et al., 2019). The quality of the raw high-throughput sequencing data was initially assessed by the FastQC program (Andrews, 2010). Unwanted or low-quality sequences were trimmed from the reads using Cutadapt and Trimmomatic software (Martin, 2011; Bolger et al., 2014). The PEAR v0.9.11 tool was employed to merge paired-end reads and generate larger amplicons of approximately 400 bp (Zhang et al., 2014). Following the identification and removal of chimeric sequences, the remaining sequences were clustered into Operational Taxonomic Units (OTUs) at a 97% similarity level. The SILVA (v.123) *rRNA* gene database was utilized for the taxonomic assignment of the categorized reads from phylum to species levels (Quast et al., 2013).

OTU saturation or sampling depth was assessed by examining rarefaction curves based on the estimated number of observed features. Alpha diversity was measured by indices such as Shannon’s entropy (Shannon et al., 1950), observed features (Yu et al., 2005), Faith’s phylogenetic diversity (PD) (Faith, 1992), and Pielou’s evenness (Pielou, 1966) was calculated using the QIIME2 v. 2018.1 package. Beta diversity analysis was performed using Bray-Curtis (Jaccard, 1908; Sorensen, 1948), Jaccard, unweighted UniFrac, and weighted UniFrac distances (Lozupone and Knight, 2005) with a sub-sampling depth of 200 sequences per sample. The non-parametric analyses of Kruskal–Wallis and permutation multivariate analysis of variance (PERMANOVA) were respectively applied to explore variations within and between groups. Principal coordinate analysis (PCoA) ordinations of bacterial community structure were computed using the ordinate function implemented in the QIIME2 v. 2018.1 tools.

Taxonomic profiles across specimens were analyzed with the aid of the program STAMP (Statistical Analysis of Metagenomic Profiles) v. 2.1.3, a graphical software package that provides statistical hypothesis tests and exploratory plots for analyzes of two samples, two groups, or multiple groups (Parks et al., 2014). Statistical analysis parameters were set to evaluate divergences among mentioned analyzes, including tests for mean differences, variance, and distributional shifts, in accordance with the software’s recommendations (Parks et al., 2014). Circos, Cytoscape, and Venn tools were used to classify and quantify the common and unique bacteria found in fleas in relation to species and sampling sites (Shannon et al., 2003; Krzywinski et al., 2009).

To determine the phylogenetic position of the bacteria detected in the fleas, the representative *16S rRNA* sequences of each bacterium were compared with the symbiotic and pathogenic counterparts reported in the literature, using maximum likelihood tree construction in MEGA software version 11. Only representative sequences >50 reads were included in the phylogenetic analysis.

## 3 Results

### 3.1 Faunistic findings

From the 67 locations investigated in Akanlu (A), Bukan (B), Sarab (S) and Tello (T), a total of 222 hosts were trapped, and 1,437 fleas were collected (**Figure 1**). Among them, 3 and 5 different mammalian host and flea species were identified, respectively. Also, 94% of the rodents were *M. persicus* (n = 209), while the other hosts were *Microtus arvalis* (n = 14) and *Vulpes vulpes* (n = 1). In addition, 93% of fleas belonged to the *Xenopsylla* genus, i.e. *X. buxtoni* (XB = 1,169) and *X. nuttalli* (XN = 171). A small number of *P. irritans* (PI = 54), *N. iranus iranus* (NI = 40), and *C. rettigi smiti* (CR = 3) were identified, as well. Finally, the collected fleas were almost equally divided between males (M) and females (F), with 622 males and 815 females (**Supplementary Table S1**). A total of 61 male fleas and 85 female fleas from all sampling sites and all hosts were selected for microbiome analysis. From now on, for the sake of shorthand, the names of the taxon, sex, and collection sites of the studied fleas will be abbreviated with four letters, indicating the name of the flea species, sex of the flea, and location of specimen isolation. For instance, XBFB represents *Xenopsylla buxtoni* females collected from Bukan. All abbreviations are provided at the end of the manuscript.

### 3.2 Taxonomic profiling and visualization of *16S rRNA* metagenomic data

The Illumina HiSeq platform detected a total of 1,482,496 sequences from 13 pooled specimens after quality filtering, trimming, and paired read merging, as well as removing chimeric sequences (**Supplementary Table S1**; **Supplementary Material 2**). The average read depth was 114,038 per specimen, with a minimum of 43,706 and a maximum of 133,952 for the XNMS and the NIMA, respectively (**Supplementary Table S2**). The rarefaction analysis indicated the adequacy of sequencing output for each specimen, as all curves reached the plateau/stationary phase along the x-axis (**Supplementary Figure S1**).

**Figure 2:**
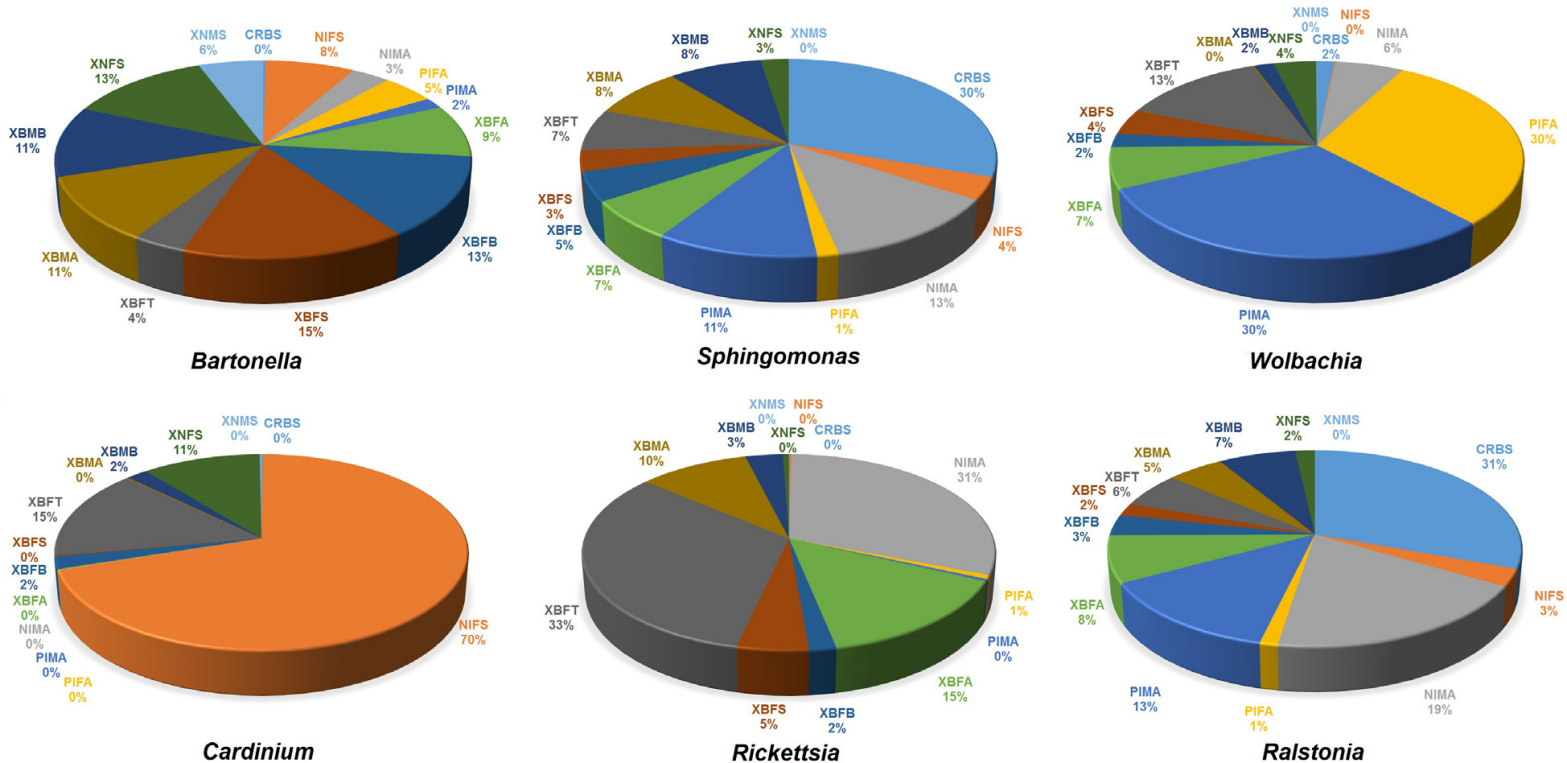
The relative frequency of six bacterial genera with high abundance in the flea specimens surveyed in this study.

The *16S rRNA*-OTUs identified from flea specimens belonged to 24 phyla, 45 classes, 108 orders, 181 families, 349 genera, and 431 species. The most abundant phyla were Proteobacteria (x̄ ~ 93%; 57-100%), Bacteroidota (x̄ ~ 5%; 0.06-42%), and Firmicutes (x̄ ~ 0.80%; 0.02-5%), with main bacteria classes, compeising Alphaproteobacteria (x̄ ~ 89%; 55-99%), Bacteroidia (x̄ ~ 5%; 0.06-42%), Gammaproteobacteria (x̄ ~ 4%; 0.42-17%), and Bacilli (x̄ ~ 0.6%; 0.01-5%). The most plentiful family was Rhizobiaceae (x̄ ~ 51%; 2-97%), followed by Sphingomonadaceae (x̄ ~ 17%; 0.004-78%), Anaplasmataceae (x̄ ~ 15%; 0.01-66%), Amoebophilaceae (x̄ ~ 5%; 0.01-41%), and Rickettsiaceae (x̄ ~ 4%; 0-19%). At the genus level, the top six genera, namely *Bartonella* (52%), *Sphingomonas* (16%), *Wolbachia* (15%), *Cardinium* (5%), *Rickettsia* (3%), and *Ralstonia* (2%), combined accounted for approximately 93% of the flea’s microbiome (**Supplementary Material 2**; **Supplementary Figure S2**). The relative frequency of these genera varied from zero to 70% in the studied specimens. The highest number of *Bartonella* sequences was found in XBFS (15%), *Sphingomonas* in CRBS (30%), *Wolbachia* in PIFA/PIMA (30%), *Cardinium* in NIFS (70%), *Rickettsia* in XBFT (33%), and *Ralstonia* in CRBS (31%) (**Figure 2**).

### 3.3 Alpha and beta-diversity analysis

The analyses differences in the structure of microbial composition within and between the compared specimens. The alpha-diversity indicated that the composition of flea microbiome depends on the location where the fleas have been collected, the flea species, and the flea gender. Notably, fleas collected from Tello and Sarab displayed the most and the least complex microbiota. Similarly, *N. iranus iranus* and *P. irritans* had the most and least complex microbiota. Lastly, males had a more complex microbiota than females. However, statistical analysis (Kruskal-Wallis) showed that only the microbiota between *P. irritans* and *X. buxtoni* was significantly different and only according to the Pielou Evenness index (*p* = 0.045; **Supplementary Table S3**; **Supplementary Figure S3**).

Assessment of beta-diversity indices using PERMANOVA statistics showed significant variations only between microbiomes from *X. buxtoni* and *P. irritans*, and between *X. buxtoni* and *X. nuttalli* (**Table 1**). However, among the four indices (Bray-Curtis (3A), Jaccard (3B), unweighted (3C), and weighted UniFrac (3D)), the Jaccard index (3B) did not yield significant results when comparing *X. buxtoni* and *P. irritans*. In contrast, it is the only index that shows a significant difference between the microbiomes of *X. buxtoni* and that of *X. nuttalli*.

**Table 1:** *Pseudo F* table of PERMANOVA analysis based on Bray-Curtis, Jaccard, and unweighted/weighted UniFrac dissimilarities.

Group 1	Group 2	Sample size	Bray-Curtis		Jaccard		Unweighted UniFrac		Weighted UniFrac	
			pseudo-F	*p* value*	pseudo-F	*p*value*	pseudo-F	*p*value*	pseudo-F	*p*value*
Female	Male	12	0.57	0.79	1.06	0.25	0.96	0.54	1.14	0.31
	Male-Female	8	1.34	0.399	1.07	0.385	1.04	0.52	2.77	0.23
Male	Male-Female	6	1.30	0.499	0.94	0.50	0.83	1.00	1.86	0.16
Akanlu	Bukan	7	1.38	0.189	0.97	0.71	1.03	0.38	1.25	0.39
	Sarab	10	1.74	0.159	1.10	0.12	1.02	0.38	2.03	0.05
	Tello	6	0.77	0.719	0.92	0.83	0.86	1.00	1.11	0.33
Bukan	Sarab	7	1.27	0.289	0.97	0.60	1.17	0.31	0.16	0.89
	Tello	3	21.16	0.349	1.20	0.36	1.33	0.33	49.26	0.33
Sarab	Tello	6	0.83	0.499	1.06	0.33	1.21	0.16	1.50	0.50
*Ctenophthalmus rettigi smiti*	*Nosopsyllus iranus iranus*	3	0.72	1.10	0.98	0.66	0.88	0.67	0.91	0.67
	*Pulex irritans*	3	14.68	0.35	1.21	0.37	1.10	0.66	21.14	0.35
	*Xenopsylla buxtoni*	7	3.65	0.13	1.09	0.29	1.27	0.27	5.04	0.15
	*Xenopsylla nuttalli*	3	5.28	0.34	0.99	0.64	0.75	1.00	11.73	0.33
*Nosopsyllus iranus iranus*	*Pulex irritans*	4	2.63	0.33	1.11	0.37	1.10	0.67	2.09	0.33
	*Xenopsylla buxtoni*	8	2.77	0.06	1.22	0.08	1.10	0.67	2.60	0.07
	*Xenopsylla nuttalli*	4	2.10	0.30	1.11	0.34	1.03	0.65	1.12	0.34
*Pulex irritans*	*Xenopsylla buxtoni*	8	9.27	**0.04**	1.14	0.06	1.70	**0.03**	5.39	**0.03**
	*Xenopsylla nuttalli*	4	12.83	0.33	1.26	0.34	1.12	0.32	15.03	0.34
*Xenopsylla buxtoni*	*Xenopsylla nuttalli*	8	3.16	0.07	1.24	**0.04**	1.14	0.32	1.32	0.28

*Only statistically significant differences (*p* ≤ 0.05) are highlighted.

**Figure 3:**
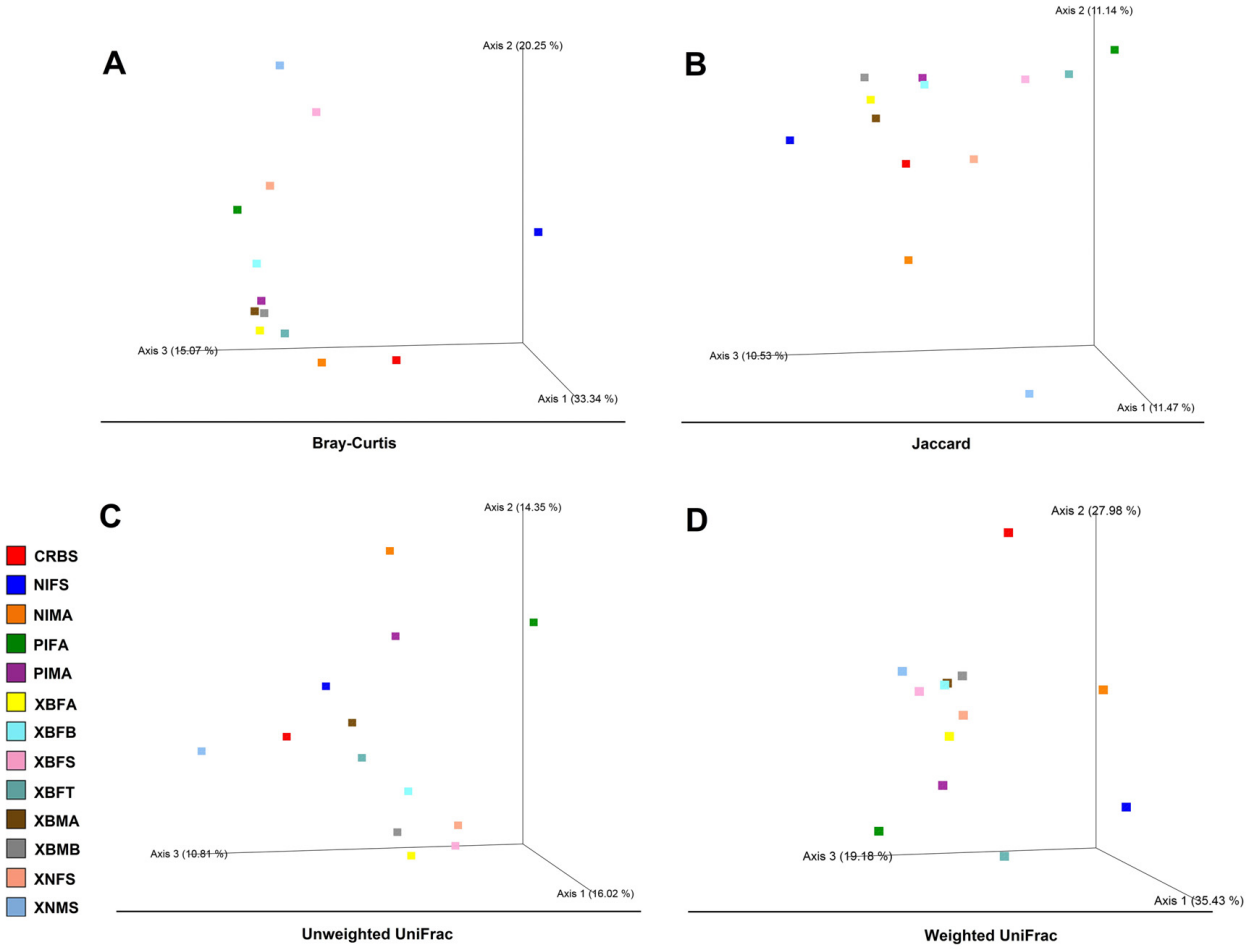
Principal coordinates analysis (PCoA) plots of bacterial beta-diversity distances [Bray−Curtis **(A)**, Jaccard **(B)**, and unweighted **(C)**/weighted UniFrac **(D)**] among flea specimens. Distances between points on the ordination plot reflect relative dissimilarities in microbiome structures. Eigenvalues of PCoA1, PCoA2, and PCoA3 are given in brackets. Samples were colored according to the field’s sources.

Overall, 68.66% and 82.59% of the variation in the bacterial community structure could be explained by the three axes based on Bray-Curtis (3A) and weighted UniFrac metrics (3D), respectively (**Figure 3**). In general, the PCoA ordinations based on Bray-Curtis (3A) distance demonstrated that the microbiomes of PIFA, PIMA, and NIFS specimens were taxonomically different from the rest of the specimens (**Figure 3**). Likewise, PCoA ordinations based on weighted UniFrac distance indicated that the microbiomes of NIFS, as well as CRBS, were divergent from other specimens (**Supplementary Figure S4**). These data reflect the greater abundance of *Cardinium*, *Ralstonia*, and *Wolbachia* in NIFS, CRBS, and PIFA/PIMA, respectively (**Figures 2**, **3**).

### 3.4 Distribution pattern of microbiome across specimens

Taxonomic profiles of bacteria across different groups were visualized using the STAMP v. 2.1.3 package. At the genus level, PCA was conducted for abundance analysis (**Supplementary Figure S4**). PC1 explained 71.1% of the variance by the two axes. The results showed significant differences between the microbiomes of *X. nuttalli* and *P. irritans*, as well as between *X. nuttalli* and *C. rettigi smiti*. In terms of location, significant differences were also found between Tello and Bukan. PC1 did not show significant differences between the microbiomes in terms of sex and genus of the studied fleas (*p* > 0.05, **Supplementary Figure S4**).

STAMP analysis of the top nine bacteria in male and female *X. buxtoni* fleas from all study areas did not show significant differences (**Supplementary Figure S5**). Comparison of these bacteria between *X. buxtoni* and *X. nuttalli* show to be nonsignificant, as well (*p* > 0.05, **Supplementary Figure S6**).

**Figure 4:**
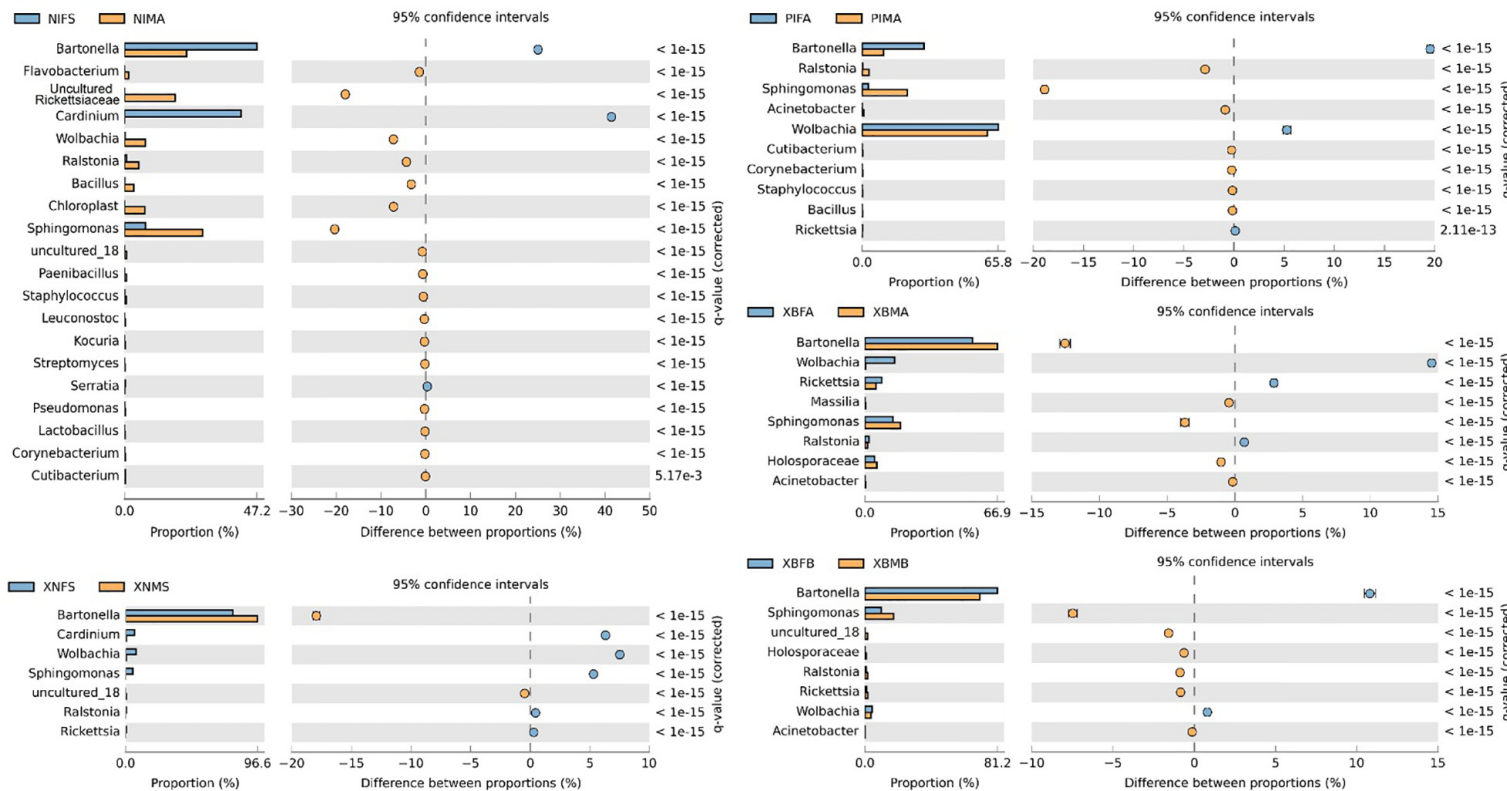
Bacterial profile (at the genus level) in male and female fleas. The profiles were generated using the STAMP package. Corrected *p* values were calculated based on Fisher’s exact test method using Storey’s FDR approach. *P* < 0.05 were considered for comparison. The blue and orange bars show a positive and negative difference, respectively. The size effect of > 200 reads is included in the comparisons. Differences between samples are shown at 95% confidence intervals. The meaning of the abbreviations is given in abbreviations.

Extended error bar diagrams were used to identify significant differences in the microbiome between the males and females of *N. iranus iranus*, *X. buxtoni*, *X. nuttalli*, and *P. irritans* species. Fisher’s exact analysis of *N. iranus iranus* fleas showed that *Bartonella* and *Cardinium* were present in greater abundance in females and lesser abundance in males with positive differences, whereas uncultured Rickettsiaceae and *Sphingomonas* were less abundant in females and more abundant in males with negative differences. In *X. nuttalli* the bacteria of *Cardinium, Wolbachia*, and *Sphingomonas* were present in greater abundance in females and *Bartonella* in males. In *P. irritans*, two bacteria, *Bartonella* and *Wolbachia*, were found to be more abundant in females than in males. This trend for *Sphingomonas* was reverse. In *X. buxtoni* fleas, *Wolbachia*, *Rickettsia* and *Bartonella* were more abundant in female fleas from Akanlu, while *Bartonella* and *Sphingomonas* were more abundant in male fleas from Bukan (**Figure 4**).

### 3.5 Effect of flea species and their location on the microbiome

**Figure 5:**
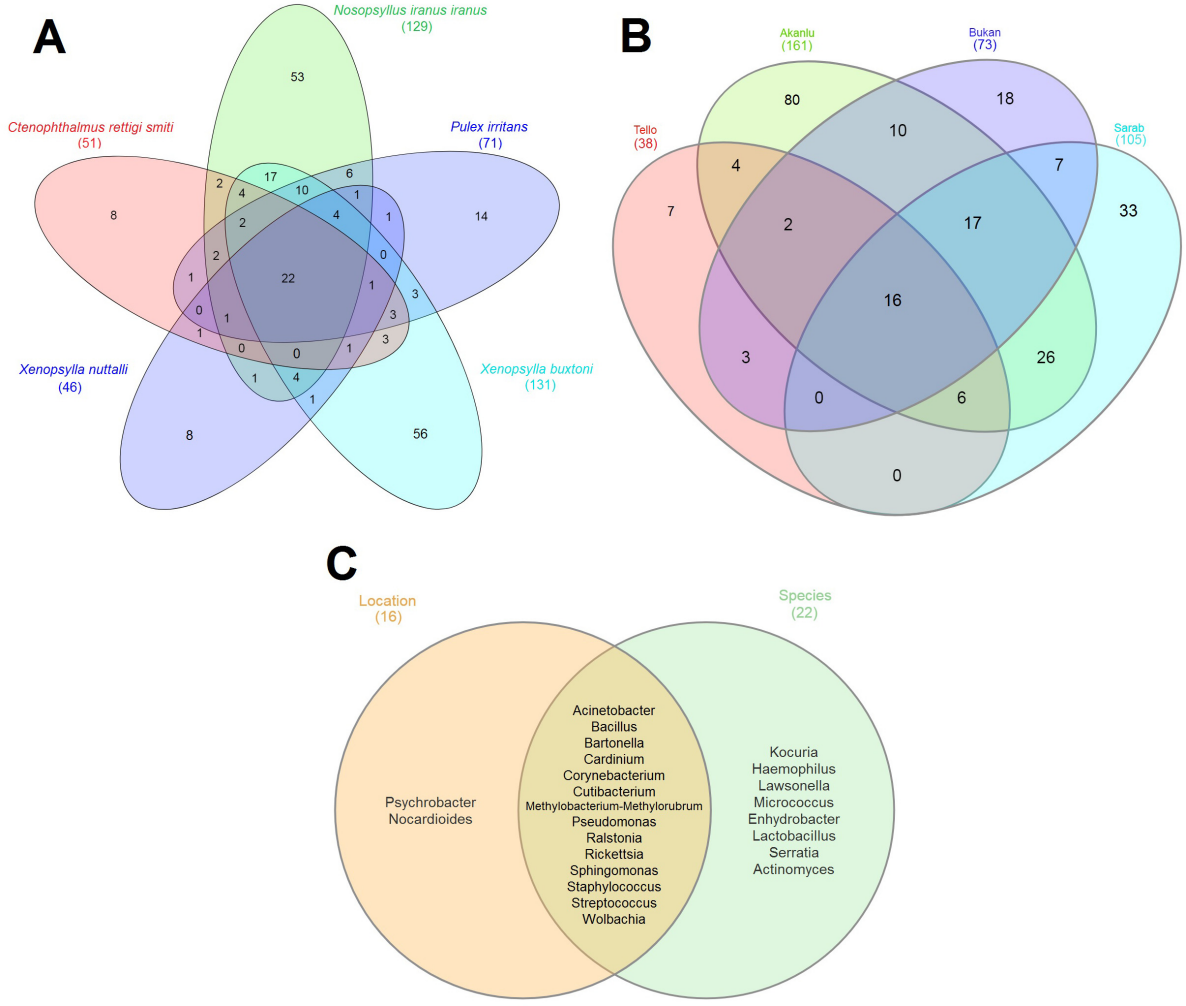
Venn diagrams showing the distribution of bacterial genera by flea species **(A)** by collection sites **(B)** and overlap of species-location **(C)**. Shared bacteria genera between sets are shown in the core. Total bacteria for each set are indicated within brackets outside the graph.

**Figure 6:**
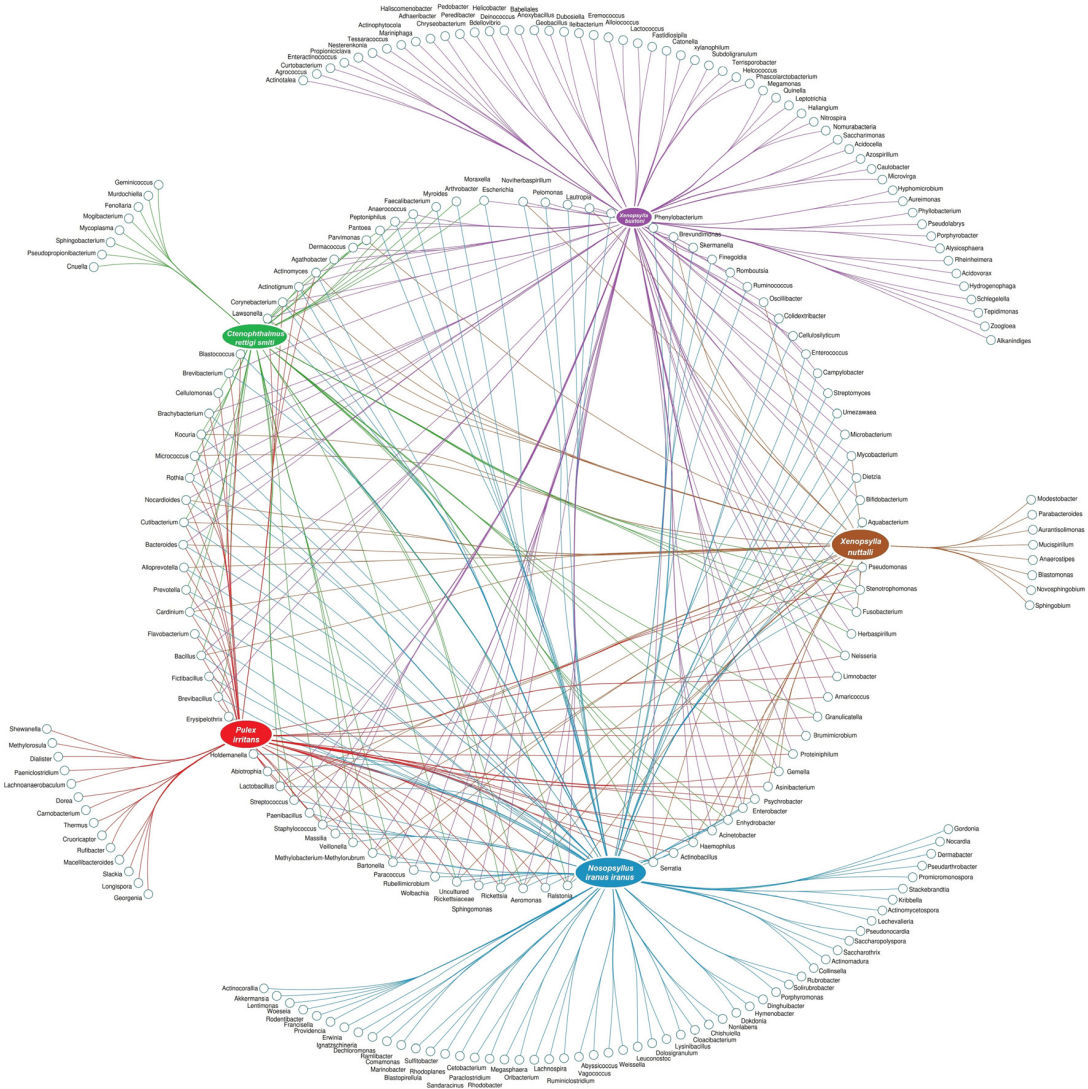
Network analysis displaying the shared and exclusive bacteria of flea specimens at the species level.

**Figure 7:**
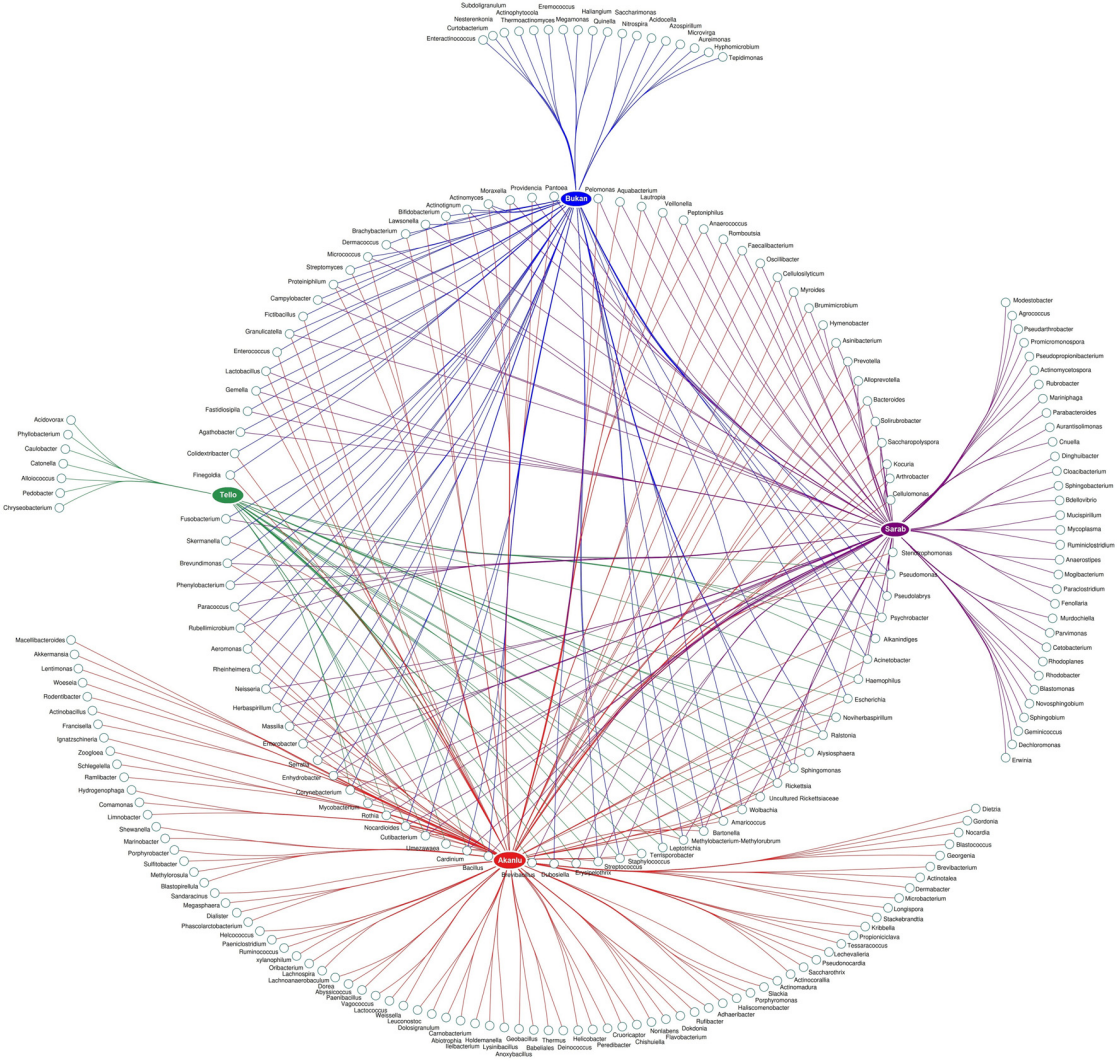
Network analysis showing the shared and unique bacteria in flea specimens across the location sites level.

To scrutinize the effect of flea species, the environment, and their interactions on the flea microbiome, the bacterial composition of five collected flea species (*N. iranus iranus*, *X. buxtoni*, *P. irritans*, *C. rettigi smiti* and *X. nuttalli*) from the four investigated locations (Akanlu, Sarab, Bukan, and Tello) was analyzed using complex data visualization platforms (**Figures 5A**, **6**). The analysis revealed the presence of 230 bacterial genera. Notably, 129 genera were identified for *N. iranus iranus*, 131 for *X. buxtoni*, 71 for *P. irritans*, 51 for *C. rettigi smiti*, and 46 for *X. nuttalli*. Also, 56 genera were unique to *X. buxtoni*, 53 to *N. iranus iranus*, 14 to *P. irritans*, and 8 to *C. rettigi smiti* and *X. nuttalli*, and 22 genera were found in all species (**Figures 5A**, **6**; **Supplementary Tables S4**, **S5**). Regarding the investigated sites, we identified 161, 105, 73, and 38 genera in fleas from Akanlu, Sarab, Bukan, and Tello, respectively (**Figure 5B**). Furthermore, 80, 33, 18, and 7 genera were found only in fleas from Akanlu, Sarab, Bukan, and Tello, respectively. A total of 16 genera were identified in all investigated locations (**Figures 5B**, **7**; **Supplementary Tables S6**, **S7**). Lastly, screening key bacteria from the co-intersection of species-location hubs revealed that 14 genera were shared among specimens regardless of the flea species and collection sites. Also, two and eight genera were unique to the studied sites and the flea species, respectively (**Figure 5C**; **Supplementary Figure S7**).

**Figure 8:**
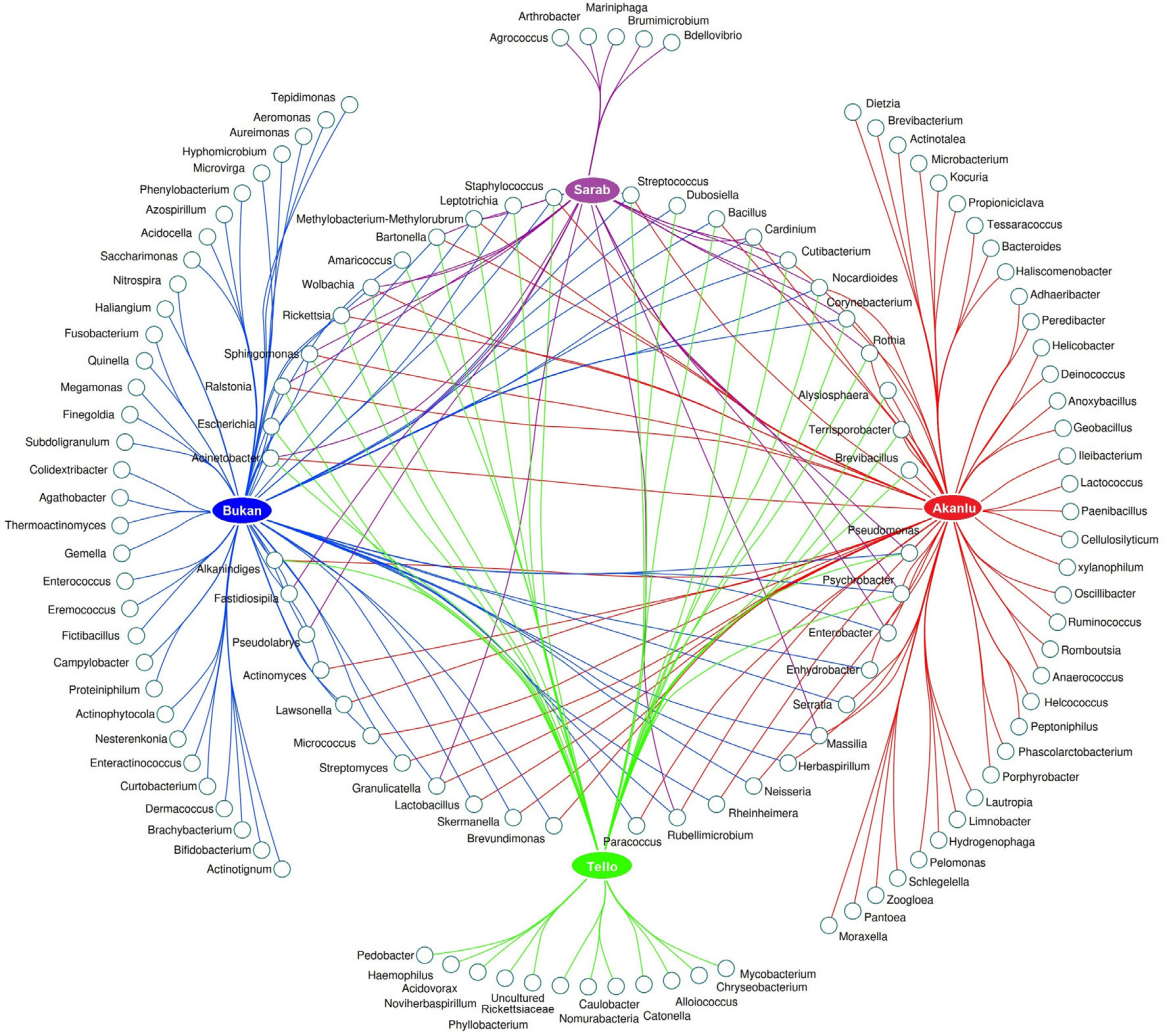
Network analysis presenting the common and unique bacteria of *X. buxtoni* specimens in the study sites.

*X. buxtoni* was the only flea species collected from all four sites; therefore, its microbiome was specifically compared across all sites. Regardless of flea gender, 44 bacterial genera were found in at least two locations. Likewise, 5, 12, 33, and 36 bacterial genera were exclusively found in fleas from Sarab, Tello, Bukan, and Akanlu, respectively (**Figure 8**; **Supplementary Figure S7**).

### 3.6 Phylogenetic inference of the bacteria

To understand the taxonomic details at levels lower than the genus and distinguish pathogenic from symbiotic bacterial species, the phylogenetic position of representative sequences of six bacterial genera (9 for *Bartonella*, 5 for *Sphingomonas*, 4 for *Wolbachia* and *Cardinium*, 2 for *Rickettsia*, and 1 for *Ralstonia*) found in this survey with high relative abundance was examined among fully identified conspecifics deposited in the Genbank database (**Supplementary Material 3**).

Phylogenetic analysis of nine *Bartonella* representative sequences obtained in the present study and 24 sequences from GenBank showed the distribution of our sequences across five distinct clusters. All compared sequences were located in the vicinity of human and rodent pathogens *Bartonella* (**Supplementary Figure S8**). The most abundant *Bartonella* sequences belonged to the isolate 9c98, which was found in all specimens (**Supplementary Table S8**).

Relationship study of five *Sphingomonas* representative sequences obtained in this study and 22 sequences from GenBank showed the clustering of our sequences in Clade 1. The compared sequences were affiliated with those isolated from water resources (*S. beigongshangi* and *S. aquatilis*) or with the plant pathogen (*S. meloni*) (**Supplementary Figure S9**). The most abundant *Sphingomonas* sequences, belonging to isolate 7,761, were found in all flea specimens except XNMS (**Supplementary Table S8**).

Phylogenetic analysis of four *Wolbachia* representative sequences obtained in this study and 40 sequences from various supergroups in GenBank showed the affinity of our sequences with supergroups A, B, and S/T (**Supplementary Figure S10**). The most abundant *Wolbachia* sequence was the isolate a0c9, which was found in all flea specimens, excluding NIFS and XNMS (**Supplementary Table S8**).

Evolutionary relationships of four *Cardinium* representative sequences obtained in this study and 15 sequences from groups A-G in GenBank demonstrated the clustering of our sequences with group A (**Supplementary Figure S11**). The most abundant *Cardinium* sequences belonged to the isolate 0920, which were found in all flea specimens, except for NIMA, PIFA, PIMA, XBFT, and XNMS (**Supplementary Table S8**).

Relationship investigation of two *Rickettsia* representative sequences obtained in this study and 18 sequences retrieved from GenBank proved the placement of our sequences within pathogenic and symbiotic *Rickettsia* Clades 1 and 2 (**Supplementary Figure S12**). The most abundant *Rickettsia* sequences were isolate 419d, which were found in all flea specimens, except XNMS (**Supplementary Table S8**).

Evolutionary inspection of one *Ralstonia* representative sequence obtained in this study and 20 sequences retrieved from GenBank revealed the clustering of our sequences in Clade 1 within human opportunistic pathogens of *R. mannitolilytica* and *R. pickettii* (**Supplementary Figure S13**). The *Ralstonia* isolate c10f was found in all flea specimens, except XNMS (**Supplementary Table S8**).

The evaluation of the bacteria identified at the species level (n = 431) showed that the bodies of fleas are a shelter for potentially human pathogenic bacteria, such as, *Acinetobacter ursingii*, *Actinomyces massiliensis*, *Corynebacterium appendicis*, *Cutibacterium avidum*, *Haemophilus parahaemolyticus*, *Leptotrichia hongkongensis*, *Mycobacterium tuberculosis*, *Prevotella oulorum*, *Pseudomonas luteola*, *Streptococcus mutans* and *Weissella viridescens* (**Supplementary Table S9**).

## 4 Discussion

Thanks to their parasitic lifestyle and association with diverse hosts, fleas are colonized with a plethora of different microbes, ranging from ancient to recently adapted pathogens and gut-associated bacteria. Herein, we reported the microbiomes of five different flea species collected from various regions in Iran. The results showed a high richness and diversity of microbiota associated with fleas, suggesting the persistence of a well-established and conserved core of bacteria. The diverse abundance of the main genera of bacteria in fleas reflects their varied ecology and adaptation processes. An interesting disparity was also detected between and within the species.

### 4.1 Bacterial isolates found and what can be suggested

Proteobacteria (primarily Alphaproteobacteria), Bacteroidota, and Firmicutes were found to be the most abundant phyla in the flea population, which is in agreement with previous studies on flea microbiotas (Lawrence et al., 2015; Dougas et al., 2021; Manvell et al., 2022; Dong et al., 2023). Six genera, *Bartonella*, *Sphingomonas*, *Wolbachia*, *Cardinium*, *Rickettsia*, and *Ralstonia*, were identified as the most abundant bacteria in the microbiome of fleas (**Figure 2**; **Supplementary Figures S2**, **S7**).

More specifically, nine phylotypes of *Bartonella* were found in the studied fleas and were grouped with the mammalian pathogen *Bartonella* species (**Supplementary Figure S8**). The most abundant isolate, 9c98, was placed next to *B. vinsonii* and *B. japonica*. Transmission of *B. vinsonii*, the causative agent of endocarditis in humans and dogs, has been questioned in previous studies involving *P. irritans* (Edouard et al., 2015; Álvarez-Fernández et al., 2018). Additionally, a strain of *B. japonica* has been identified in the blood of a small Japanese rat, *Apodemus argenteus* (Inoue et al., 2010). Therefore, it supposed that *Bartonella* isolate 9c98 is presumably transmitted by the flea species under investigation and may be implicated in the development of diseases, which call for future investigations. Broadly speaking, each *Bartonella* species is typically transmitted among mammalian hosts by a specific insect vector (Minnick and Anderson, 2006), i.e. vector specificity. This characteristic is confirmed in the current survey by the detection of distinct isolates of *Bartonella* in specific flea species (**Supplementary Table S8**).

*Sphingomonas*, which is isolated from the digestive tract, salivary glands, or reproductive organs of several arthropods (Weiss and Aksoy, 2011; Boissière et al., 2012; Minard et al., 2017; Sonenshine and Stewart, 2021), was shown to help the monophagous scale insect *Steingelia gorodetskia* reside on the roots of birch trees (Michalik et al., 2019). Hence, *Sphingomonas* may support fleas, as well, in adapting to the use of host blood sources, as it is considered a commensal bacterium of insects, which was more or less found in all the specimens of present study (**Figure 2**). Interestingly, the paratransgenic candidate *Sphingomonas* may affect the ability of vector to transmit pathogens, reduce the infection of *Ixodes scapularis* ticks and lower the risk of human anaplasmosis (Mazuecos et al., 2023). Also, glycosphingolipids from *S. paucimobilis* have been included in a nanovaccine formulation, leading to increased efficacy through reducing biofilm formation by *Acinetobacter baumannii* (Khan et al., 2022). Since biofilm formation is the main mechanism needed for *Y. pestis* transmission by fleas (Hinnebusch and Erickson, 2008), the interaction between *Sphingomonas* and *Yersinia* in the gut of plague-carrying fleas is plausible. The presence of *Sphingomonas* could potentially benefit the flea and/or affect the transmission or outcome of the disease.

*Wolbachia*, *Rickettsia*, and *Cardinium* bacteria are common maternally inherited endosymbionts found in many insect groups (Curry et al., 2015; Sazama et al., 2019). The popularity of these symbionts arises from their ability to manipulate the reproduction of the host insects (Thomas, 2016), interact with main human pathogens, maintain the homeostasis of the insects immune system, nutrition, xenobiotic metabolism and provide protection against environmental stress and defense (Eleftherianos et al., 2013; Liu and Zheng, 2019; Maleki-Ravasan et al., 2019; Fan et al., 2022). In this study, the distribution of sequences related to each of the three endosymbionts under investigation was notably different in certain samples. For example, *Wolbachia* was found in PIMA/PIFA, whereas *Cardinium* was identified in NIFS. In the case of *Wolbachia*, similar isolates were found with an equal ratio of 30%/30% in PIMA/PIFA (**Figure 2**). This observation most likely indicates the absence of *Wolbachia*-induced reproductive effects, such as cytoplasmic incompatibility, on the *P. irritans* population. These findings are in line with Flatau et al.; however, they had only reported the presence of *Wolbachia* in the female insects. This discrepancy could be related to the heterogeneity of the studied flea species (Flatau et al., 2021). Phylogenetic study showed the presence of two supergroups, A and B *Wolbachia*, and an unidentified supergroup placed between the S and T supergroups in the studied fleas (**Supplementary Figure S10**). Supergroups A and B are often found in arthropods with roles in reproductive manipulation (Karami et al., 2016; Karimian et al., 2018) while S and T have recently been identified in Pseudoscorpions (Lefoulon et al., 2020) and bed bugs (Laidoudi et al., 2020). In both hosts, their main physiological role is to provide B vitamins. This unspecified supergroup, which was explored in all studied fleas, needs to be accurately identified and determine its supportive role.

While *Cardinium* made up 70% of the NIFS microbiome, no trace of this bacterium was found in its male counterpart, NIMA (**Figure 2**). Similar to our survey, the genus *Cardinium* has been identified as the dominant endosymbiont following *Wolbachia* in echidna stickfast flea, *Echidnophaga ambulans* (Lawrence et al., 2015). All the *Cardinium* isolates of this study were classified in group A, which comprises the prevalent isolates infecting insects (Stouthamer et al., 2019). Since *Cardinium* was absent in male fleas from populations such as NIMA, it is hypothesized that this bacterium may be involved in the male-killing phenotype, which warrants further investigation. In various studies, *Cardinium* has been associated with insects with a limited host range, such as sap-sucking insects, and their parasitoids (Zchori-Fein and Perlman, 2004; Gruwell et al., 2009), as well as *Oropsylla* and *Echidnophaga* fleas (Jones et al., 2012; Lawrence et al., 2015). However, in this study, it was detected in the wide host range flea, *Nosopsyllus* (Maleki-Ravasan et al., 2017), which highlights the significance of studying the role of *Cardinium* in the bioecology of these hosts. In addition, *Cardinium* was suggested to help a thermophilic Acarine adapt to low temperatures (Konecka and Olszanowski, 2019). This adaptability is not surprising in the case of *Nosopsyllus* fleas that were caught in the cold regions of northwestern Iran.

Triple and double infections of fleas populations with *Wolbachia*, *Cardinium* and *Rickettsia* endosymbionts were observed in this study. Triple infections were found in males and females of 10 out of 13 studied specimens. *Wolbachia*-*Rickettsia* infections were discovered in NIMA and PIMA males, whereas *Wolbachia*-*Cardinium* was identified in XNMS males. Remarkably, the co-occurrence of *Wolbachia* with each of the other two symbionts was found in male fleas (**Supplementary Table S8**). The occurrence of double endosymbionts in arthropods has been reported in a few sources (Ros et al., 2012; Brown et al., 2018; Li et al., 2020). However, the detection of three arthropod master endosymbionts in a flea population is reported for the first time in this study. Flea populations appear to be the site of intricate, dynamic, network interactions among master endosymbionts that have positive effects on the host’s feeding physiology, and microbial contents, in addition to fitness and adaptation benefits.

*Ralstonia pickettii*, isolated from various sources (Stelzmueller et al., 2006), is recognized as a conditional pathogen and an emerging nosocomial infection (Nasir et al., 2019). The bacterium has been isolated from the intestines of unfed ticks (Song et al., 2021), house flies (Gupta et al., 2012) and sand flies (Sant’Anna et al., 2012; Karimian et al., 2019). Colonization of *Lutzomyia longipalpis* gut by *Leishmania* parasites may be influenced by secondary metabolites of *R. pickettii* origin (Tobias, 2016). *R. pickettii* may assist in the nitrogen fixation of certain insects (Paulson et al., 2014; Thanganathan et al., 2021). Also, due to its ability to produce biosurfactants, *R. pickettii* has the potential to mitigate some organic pollutants (Setyo et al., 2018). Based on the available evidence and the presence of *Ralstonia* in all the studied fleas, it seems that this human pathogenic bacterium has a vital role in the biology of fleas, demanding for further targeted research.

### 4.2 Why do varied microbiomes exist between fleas from the same species but different location and/or host? What is the role of flea gender?

In the current study, the diversities of bacterial communities associated with fleas were estimated using different measures, but the results of Shannon’s entropy/Faith’s PD and Bray-Curtis/weighted UniFrac metrics, were considered to infer respectively alpha and beta diversities, as in previous studies (Chamankar et al., 2023). Collectively, bacterial richness displayed a declining tendency in five species of NII > XB > XN > CRS > PI and four locations of Tello > Akanlu > Bukan > Sarab and sex of male > female, respectively (**Supplementary Figure S3**; **Supplementary Table S3**). As shown previously, these patterns are mediated by the biotic and non-biotic factors affecting the composition of the microbiome, including host phylogeny, host diet and local environment, and the capability to transfer bacteria to progeny (Engel and Moran, 2013; Lange et al., 2023; Magoga et al., 2023).

Host species identity can dictate which microbes survive within the body (Maleki-Ravasan et al., 2015; Maleki-Ravasan et al., 2020; Malacrinò, 2022; Paddock et al., 2022). The studied fleas belonged to five species and four genera with completely different bio-ecologies (Maleki-Ravasan et al., 2017); therefore, the diversity of their microbiome seems rational. In a study, flea species showed the greatest impact on the structure of the bacterial community, with each flea species harboring unique bacterial lineages (Jones et al., 2015). One of the factors influencing the vector competence, and consequently the microbiome of flea species could be variances in the anatomy of the proventriculus (Bland and Hinnebusch, 2016). During feeding, the proventriculus rhythmically opens and closes with contractions of pharyngeal peristaltic pump muscles, thus aiding the entry and exit of microbes. The proventriculus can act as a morphological intestinal filter to protect the insect’s gut microbiome from disruption as shown in *Cephalotes rohweri* (Lanan et al., 2016). To maintain transmission to the mammalian host, *Y. pestis* is required to be able to block the flea proventriculus. This blockage mainly results from the replication of *Y. pestis* trapped in the anterior half of the proventriculus (Dewitte et al., 2023). After feeding, the peristaltic waves of the midgut and the continuous beats of the proventriculus probably break down the red blood cells, mixing them with digestive enzymes (Hinnebusch et al., 2021) and releasing intracellular bacteria such as *Bartonella*. Therefore, the proventriculus may play a central role in the acquisition and maintenance of the microbiome, and its microstructure and filtering should be investigated in different flea species.

Diet is an important factor in shaping an insect’s phenotype and gut bacterial community, which often engages in diverse symbiotic interactions with the host (Luo et al., 2021). Hematophagous ectoparasites ingest large amounts of blood containing host antibodies, complement proteins, and immune cells, which may lead to taxon-specific manipulation of the microbiota (Maitre et al., 2022). Moreover, bacteria in insects regulate the microbial population through resource limitation or niche partitioning by digesting the feeding substrate (Mason et al., 2020; Brochet et al., 2021). The higher diversity observed in the microbiome of the three flea species, NII, XB, and XN, compared to CRS and PI, is likely related to their respective hosts, *M. persicus*, *M. arvalis*, and *V. vulpes*. This relationship requires additional investigation under controlled conditions and precise methodology.

The host’s external environment may also have a significant effect on the insect microbial communities. According to Lange et al., the diversity of insect microbiomes in different local habitats might be affected by climatic conditions and human activities (Lange et al., 2023). The three studied regions in the West and Northwest of Iran, where plague outbreaks occurred, are cold and mountainous regions, while the control region is located in milder rural environment. Tello, the control region in the northeast of Tehran, has experienced significant environmental alterations in recent years. Moreover, there is a large population of stray dogs in the area, which could affect the diversity of the microbial flora of fleas. Of course, we should not forget the effect of carrying pathogens on the diversity of flea microbiota in endemic areas (Pornwiroon et al., 2007).

In this study, it was not possible to sequence the microbiome of all flea species from all locations; therefore, the effect of location cannot be measured in the same way that species affect the microbiome. For this purpose, we considered only the *X. buxtoni* microbiome that was sequenced from all four regions. Among the 130 bacterial genera identified in this flea species, 44 genera were found in all locations. Therefore, these bacteria are not location-dependent but rather species-specific. On the other hand, the number 5, 12, 33 and 36 unique genera were identified from *X. buxtoni* Sarab, Tello, Bukan and Akanlu specimens, respectively. This observation indicates that the mentioned bacteria are location-dependent. Since the ratio of location-dependent bacteria is lower than the shared bacteria among all locations, it can be simply concluded that the microbiome in *X. buxtoni* is more influenced by flea species than by location.

In general, the results of the present study indicate that the species richness of the microbiome in male fleas is higher than that in females, although the difference is not statistically significant (**Supplementary Figure S3**). More extensive analyses, on the other hand, revealed substantial variations in the microbiomes of males and females of different species in various locations, as well as within the same species in two distinct regions (**Figure 4**). Apart from the taxonomy of the host and the environment, the sources of these discrepancies can be found in the influence of other factors, e.g. obligatory relationships of the flea host and specific endosymbiont, fitness advantages conferred by the symbiont, and reproductive manipulations enforced by the endosymbiont (Flatau et al., 2021).

### 4.3 Mode of transmission: horizontal and/or vertical transmission

The presence of core bacteria in the vector population suggests that horizontal and vertical transmission is in progress (Chamankar et al., 2023). The efficiency of these pathways in establishing the host microbiome has extensively been studied in the ectoparasites such as ticks (Krawczyk et al., 2022; Du et al., 2023), and to a lesser extent in the case of flea-related Rickettsia (Legendre and Macaluso, 2017). The significant impact of flea taxonomy on the microbiome can be attributed to the transmission of microbiomes through these methods. The feeding patterns of larvae and adult fleas, along with the intracellularity of common bacteria found in this study, confirm this claim. Both pathways ultimately promote intraspecific microbiome variation and stochasticity (Lange et al., 2023).

### 4.4 Importance of separating the good from the bad: symbionts versus pathogens

During evolution, fleas appeared later than ticks, and mosquitoes and have revealed primitive and partial relationships with the pathogens they carry, both of which could be important in their adaptation, as well as in the occurrence of emerging and re-emerging diseases (Durden, 2019; Bibikova, 1977). This study provided evidence that a wide range of pathogenic bacteria (as detailed in **Supplementary Table S9**), especially biofilm-forming *Bartonella*, *Sphingomonas*, and *Ralstonia* ones (Gusman et al., 2010; Müller et al., 2011; Liu et al., 2016), are associated with fleas. These bacteria may come into contact with the vertebrate host during flea bites or feces, eventually leading to the infection. Therefore, the potential of these bacteria to cause infection and the role of fleas in their transmission, as emphasized in the literature (Dougas et al., 2021), needs further consideration.

The risk of human plague is highest in areas where the natural foci of plague and human populations intersect, as the foci examined in the current study (WHO, 2022). As a general rule, plague is characterized by long periods of apparent quiescence that are punctuated by epizootic periods (Eisen et al., 2021). During the inactive phase, *Y. pestis* can persist in soil, soil protozoa, and frozen or soft tissues for a long time, or it can circulate at low intensity in small foci among reservoir hosts (Baltazard and Bahmanyar, 1960b; Ryan, 2016; Benavides-Montaño and Vadyvaloo, 2017; Malek et al., 2017). Nevertheless, during epizootic periods, rodent deaths often occur in large numbers, and their accompanying infectious fleas seek new hosts, including humans. As a result, it is almost impossible to forecast the exact location and timing of potentially dangerous epizootics (Eisen et al., 2021). Regrettably, disease outbreaks are unavoidable since rodents and their fleas are resistant to pesticides (Rahelinirina et al., 2022). Besides, fleas have not been studied as hosts for a wide range of pathogens other than the plague bacilli, and these diseases may reappear in epidemic form (Bitam et al., 2010). Thus, microbial control strategies can be a suitable and effective solution for the control of ancient and emerging flea-borne pathogens. On the one hand, the abundance of endosymbiotic bacteria and other microbes transmitted by flea determines the overall abundance of insect microbes (Cohen et al., 2015). Indeed, these bacteria interact with *Y. pestis* in fleas (Jones et al., 2015), and some possible candidates were discussed. On the other hand, depending on the case, three intracellular bacteria, *Wolbachia*, *Cardinium*, and *Rickettsia*, can be used to endanger the lives of fleas and disrupt their sex ratio. Ultimately, a scalable, sustainable and cost-effective paratransgenesis strategy using *Sphingomonas* or *Ralstonia* bacteria to control flea-borne diseases can be recommended.

In general, in microbiome studies, it is essential to include controls to avoid any DNA contamination. In this study, DNA from different flea species was extracted at different times, and the reported bacteria have also been reported in metagenomic studies of other insects. Therefore, it can be safely claimed that in the present study, bacterial contamination of unwanted origin is minimal.

The rarefaction analysis conducted in the present study demonstrated that the specimens examined were sufficient to recover the maximum possible OTU richness from fleas. However, additional replication efforts are necessary to obtain the microbiome information from all specimens across all species, geographic locations, and allotypes.

## 5 Conclusion

In this study, the microbiomes of five flea species were investigated in areas with and without historical plague occurrence. Our findings revealed that flea microbiomes were primarily shaped by species differences, with location playing a secondary role. However, the distribution of six key bacteria—*Bartonella*, *Sphingomonas*, *Wolbachia*, *Cardinium*, *Rickettsia*, and *Ralstonia* —was not significantly influenced by either species or location. Notably, flea microbiomes exhibited a greater abundance of pathogens compared to symbionts, likely reflecting the fleas’ ectoparasitic lifestyle and blood-feeding habits. *Bartonella* dominated the microbiomes, accounting for more than half of the microbial composition, with distinct phylotypes associated with specific flea species. Given the zoonotic potential of *Bartonella* and its transmission via fleas, these findings underscore the importance of integrating One Health approaches into veterinary and human medicine. The results not only enhance our understanding of flea-borne zoonotic diseases but also highlight the potential of symbiotic bacteria as tools for preventive strategies and safe, effective control measures during future plague epizootics.

## Data Availability

The datasets presented in this study can be found in online repositories. The names of the repository/repositories and accession number(s) can be found below: https://www.ncbi.nlm.nih.gov/genbank/, PRJNA995036.
