# Supplementary material for: The interplay between species and locations shapes vector fleas microbial communities in plague foci: pathogens rather than symbionts?
Source: Front Cell Infect Microbiol. 2025 May 20;15:1568103. doi: 10.3389/fcimb.2025.1568103 (PMC12129938; doi:10.3389/fcimb.2025.1568103)
Supplement: Supplementary file 1 [file DataSheet1.docx]

**SUPPLEMENTARY FILE 1**

**The interplay between species and locations shapes vector fleas microbial communities in plague foci: pathogens rather than symbionts?**


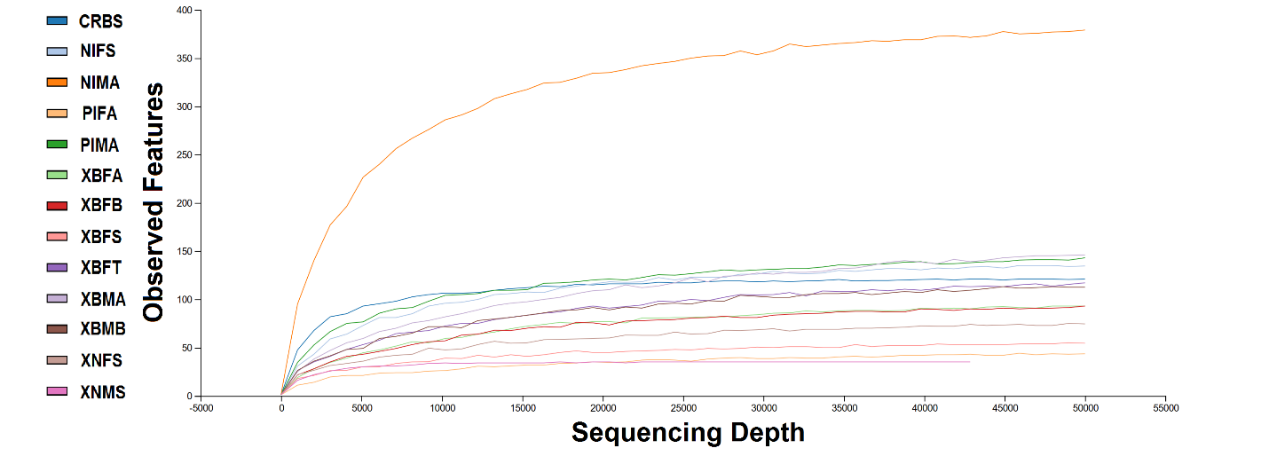


**Figure S1.** Rarefaction analysis of observed features of the microbiome of flea’s specimens.


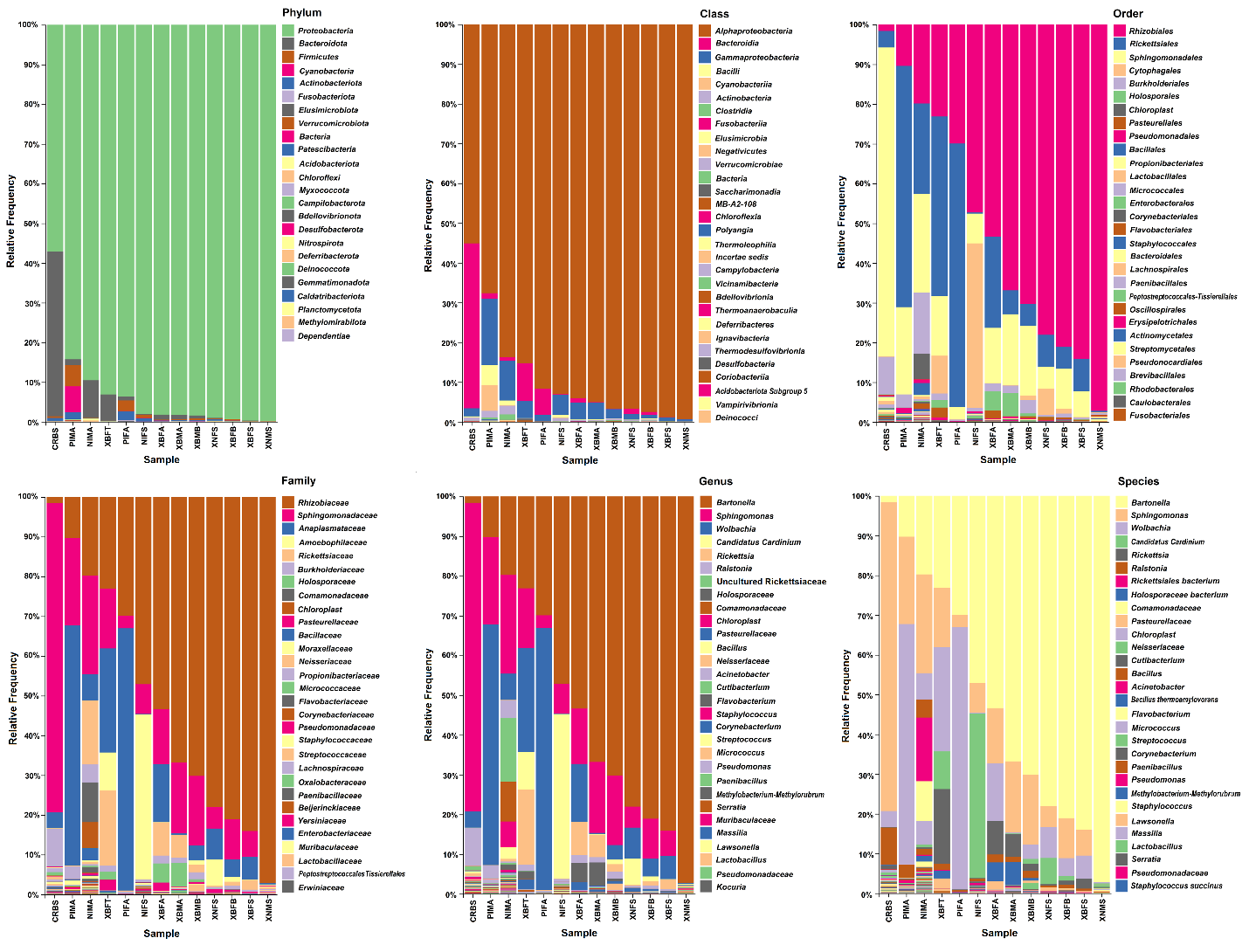


**Figure S2.** Taxonomical composition and relative frequency of the microbiome of fleas in Iran, from phylum to species. Legends are shown for 30 OTUs with high frequency.

*
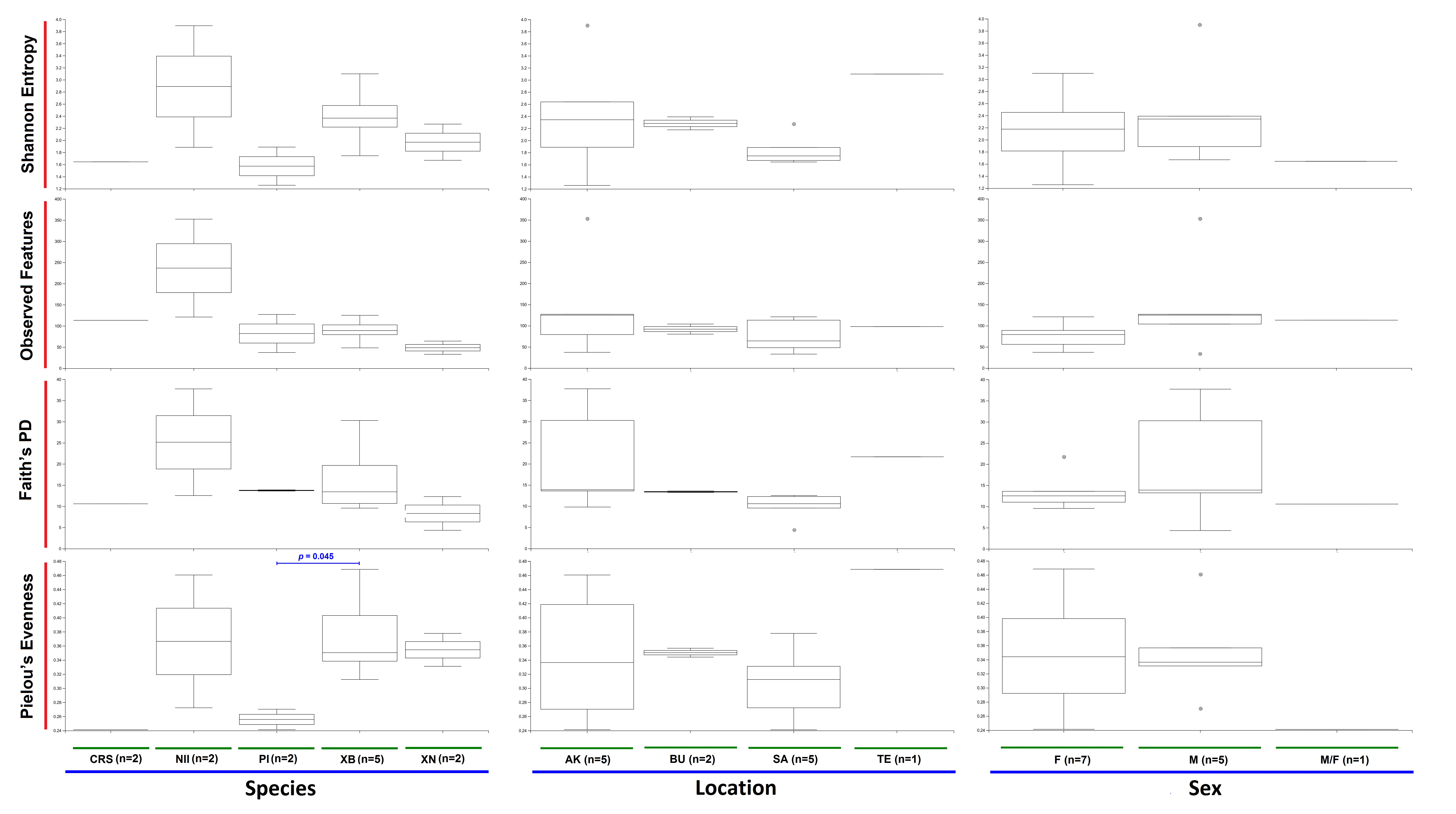
*

**Figure S3.** Box plots of alpha-diversity indices (Shannon Entropy, Observed Features, Faith PD and Pielou Evenness) comparing microbiome in terms of species, locality, and gender of studied fleas specimens. Only significant p-value (≤0.05) is shown in blue color.


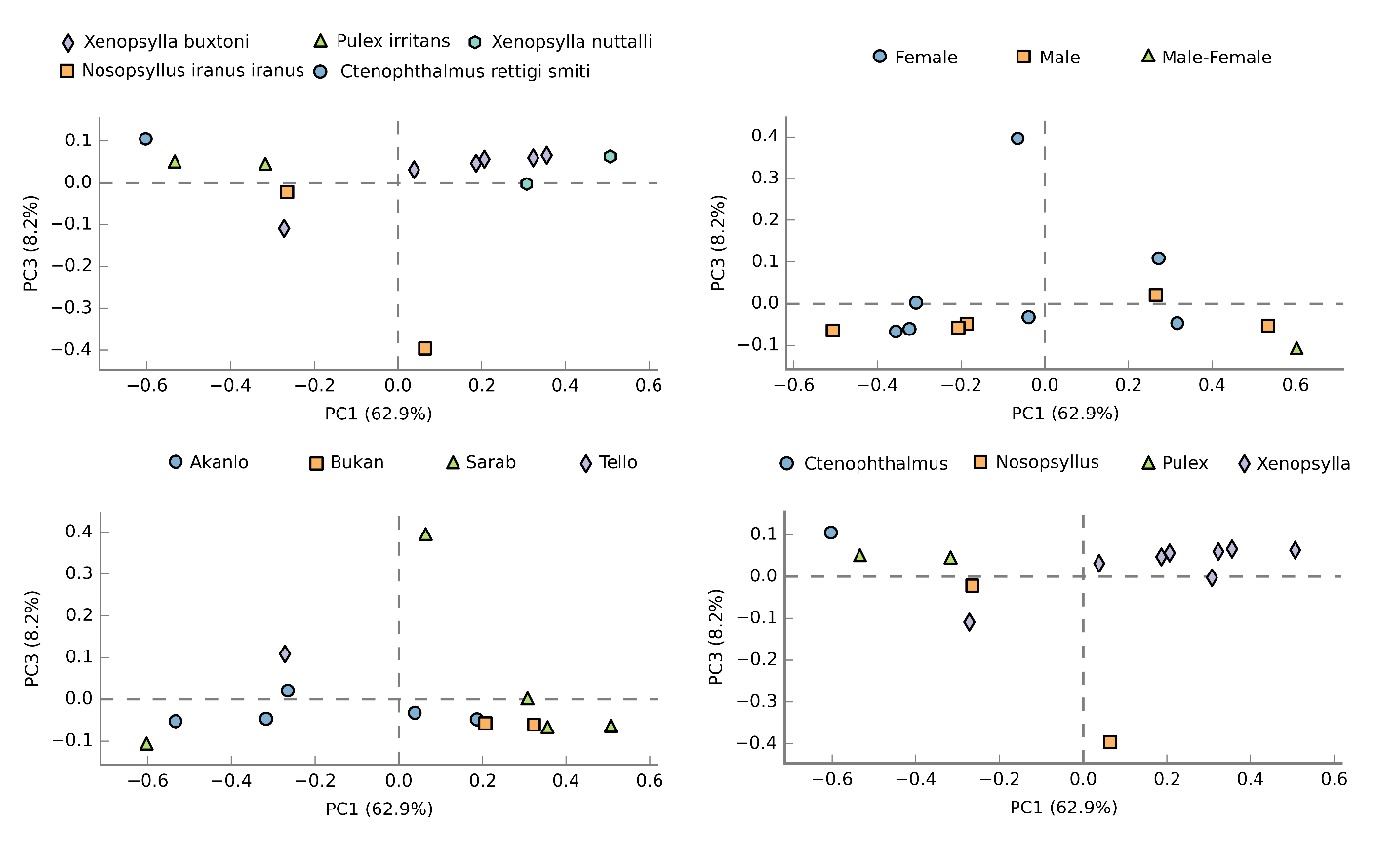


**Figure S4.** Principal component analysis (PCA) of the abundance of bacterial communities in multiple groups of five species, different sex, various locations and four genus of fleas in Iran. Bacteria from different groups are shown in different shapes/colors. Only PC1 is shown for each comparison.


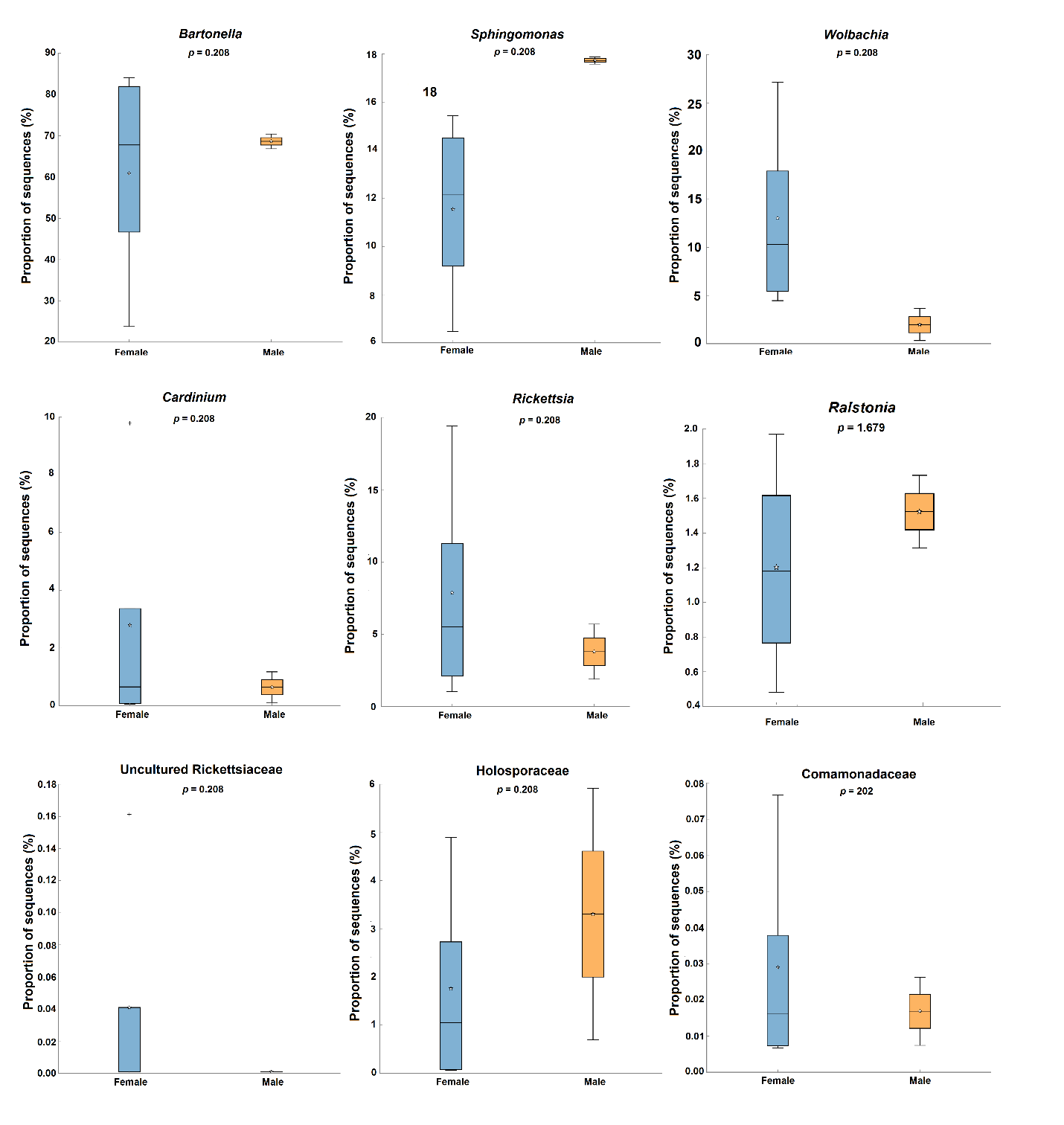


**Figure S5.** Box plot analysis of nine bacterial genera of *Bartonella*, *Sphingomonas*, *Wolbachia*, *Crdinium*, *Rickettsia*, *Ralstonia*, Uncultured Rickettsiaceae, *Holosporaceae*, and *Comamonadaceae* with high abundance in female and male *Xenopsylla buxtoni*.


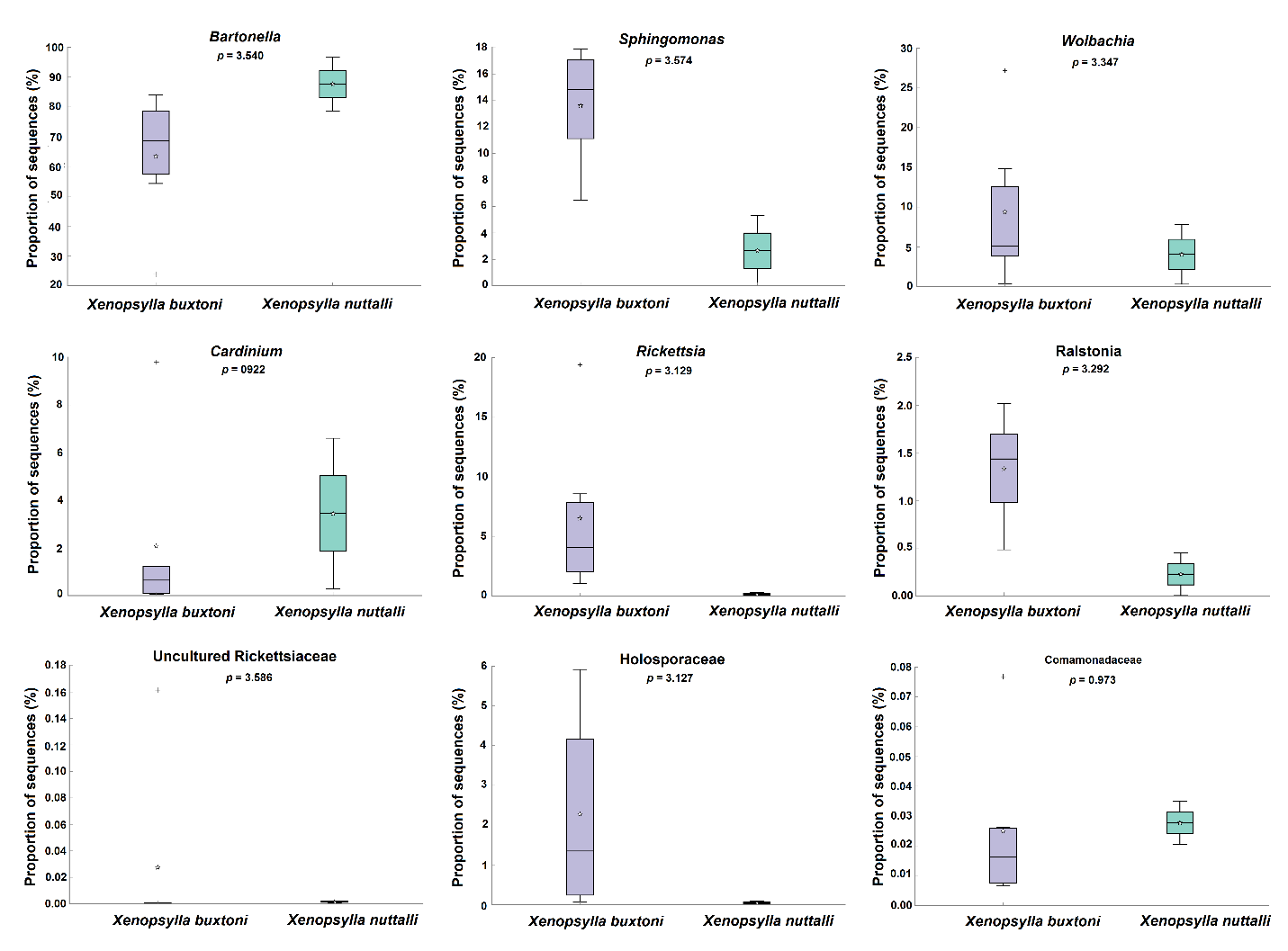


**Figure S6.** Box plot analysis of nine bacterial genera of *Bartonella*, *Sphingomonas*, *Wolbachia*, *Crdinium*, *Rickettsia*, *Ralstonia*, Uncultured Rickettsiaceae, *Holosporaceae*, and *Comamonadaceae* with high abundance in *Xenopsylla buxtoni* and *Xenopsylla nuttalli*.


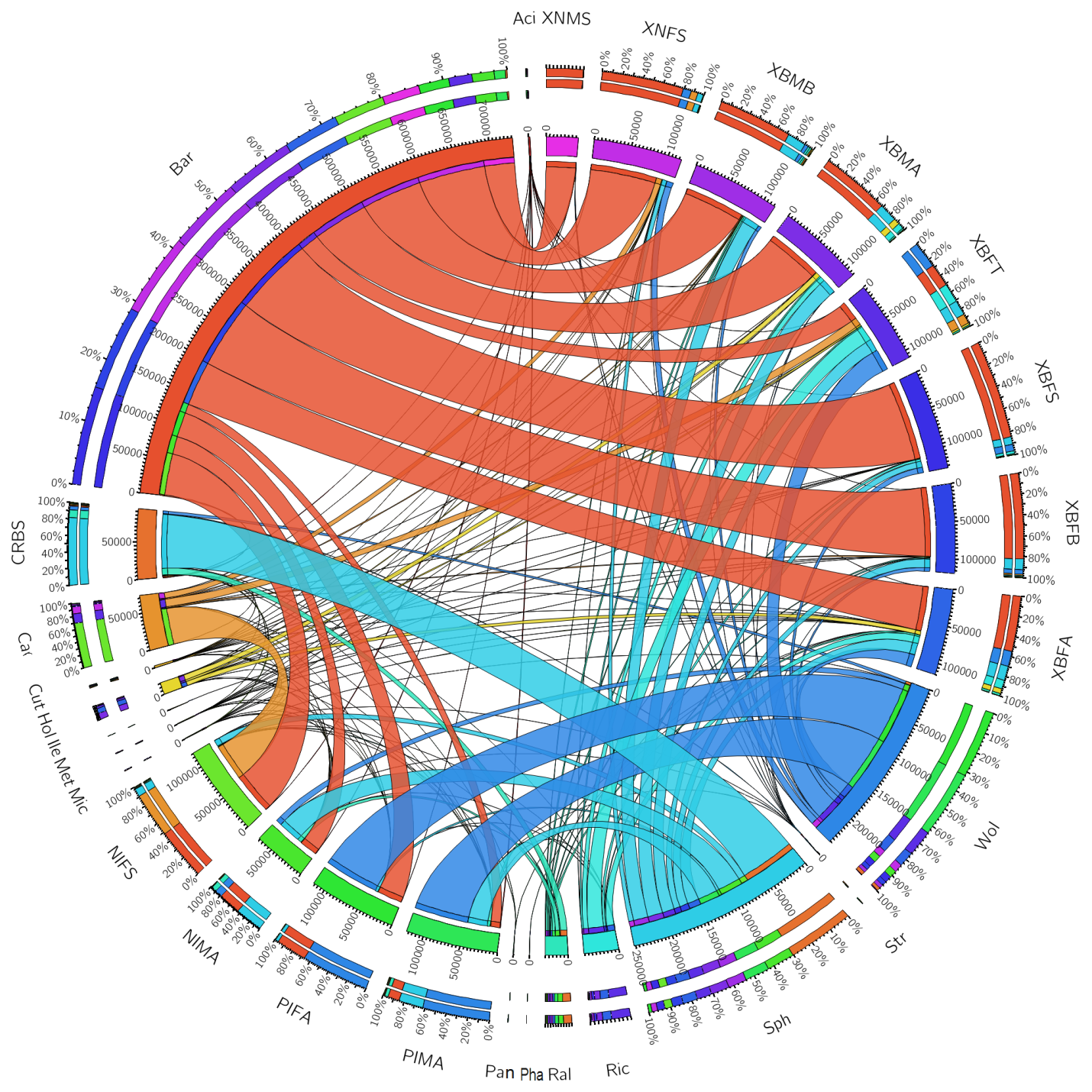


**Figure S7.** Nexus analysis representing the shared and exclusive bacterial genera identified from 13 pooled specimens of fleas collected from provinces with (Hamadan, East and West Azerbaijan) or without (Tehran) plague outbreaks. Only a distribution of fifteen bacteria is shown. Bar, *Bartonella*; Sph, *Sphingomonas*; Wol, *Wolbachia*; Car: *Cardinium*; Ric, *Rickettsia*; Ral, *Ralstonia*; Hol, *Holosporaceae*; Aci, *Acinetobacter*; Cut, *Cutibacterium*; Mic, *Micrococcus*; Met, *Methylobacterium*; Pan, *Pantoea*; Str, *Streptococcus*; Ile, *Ileibacterium* and Pha, *Phascolarctobacterium*. The full specifications of the samples, including species, gender and location, are given in the abbreviations at the end of the article.


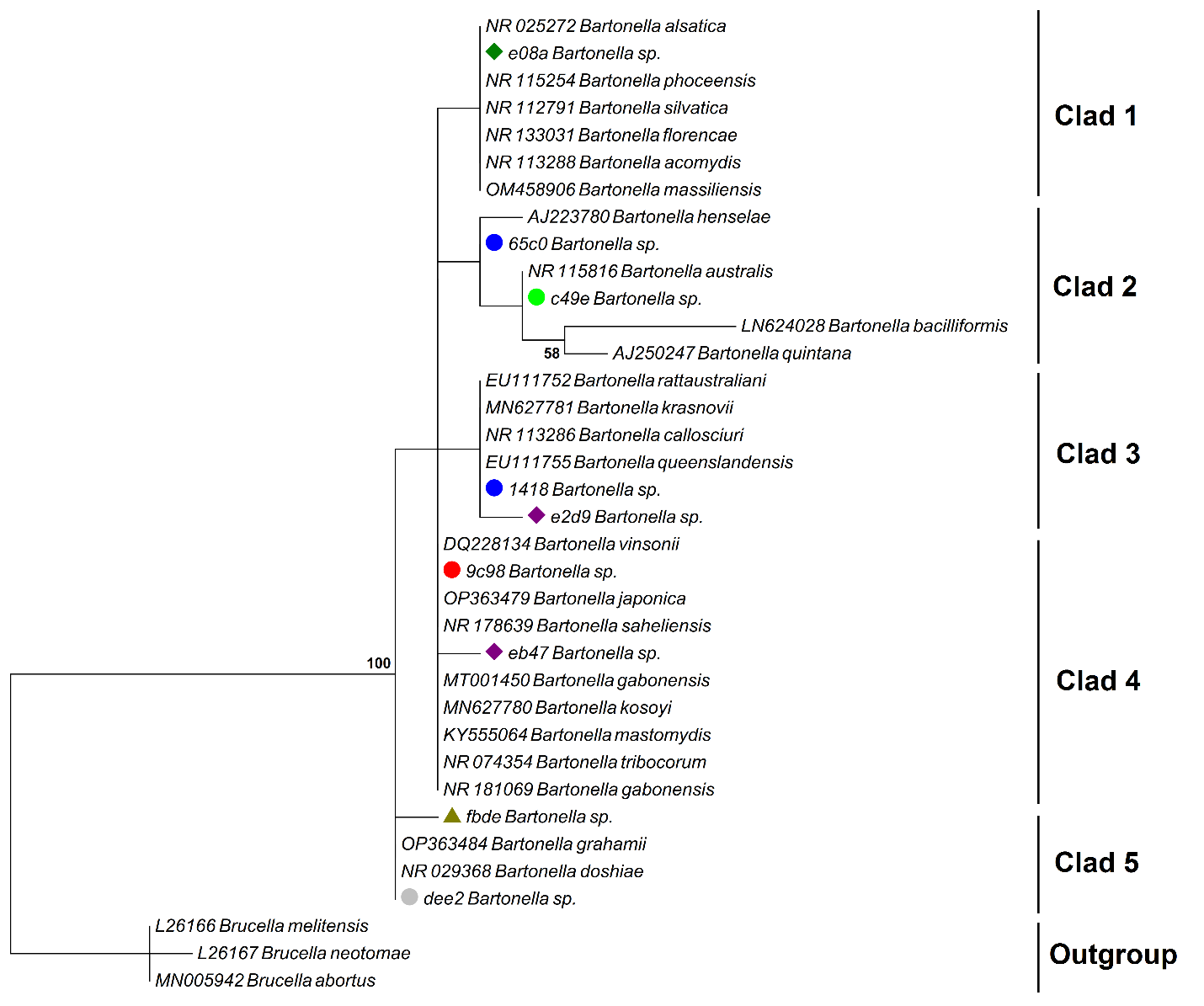


**Figure S8.** Maximum likelihood tree inferred from 404 bp of the *16S rRNA* gene sequences showing the position of nine *Bartonella* sp. obtained in this study among 24 other *Bartonella* spp. reported in literature. Only representative sequences higher than 50 reads was included in the analysis. Green diamond: XBFS, XBMB, and XNFS; purple diamond: XBFS; red circle: all specimens; blue circle: all specimens excluding PIMA and PIFA; gray circle: all specimens excluding PIMA, PIFA and NIMA; light green circle: all specimens excluding NIFS, XBFA, XBMA, XNMS; and olive triangle: NIFS. The sequences of *Brucella* spp. (L26166, L26167 and MN005942) were set as outgroup. Only bootstrap values higher than 50% are shown on the branches. The bar indicates substitutions per site.


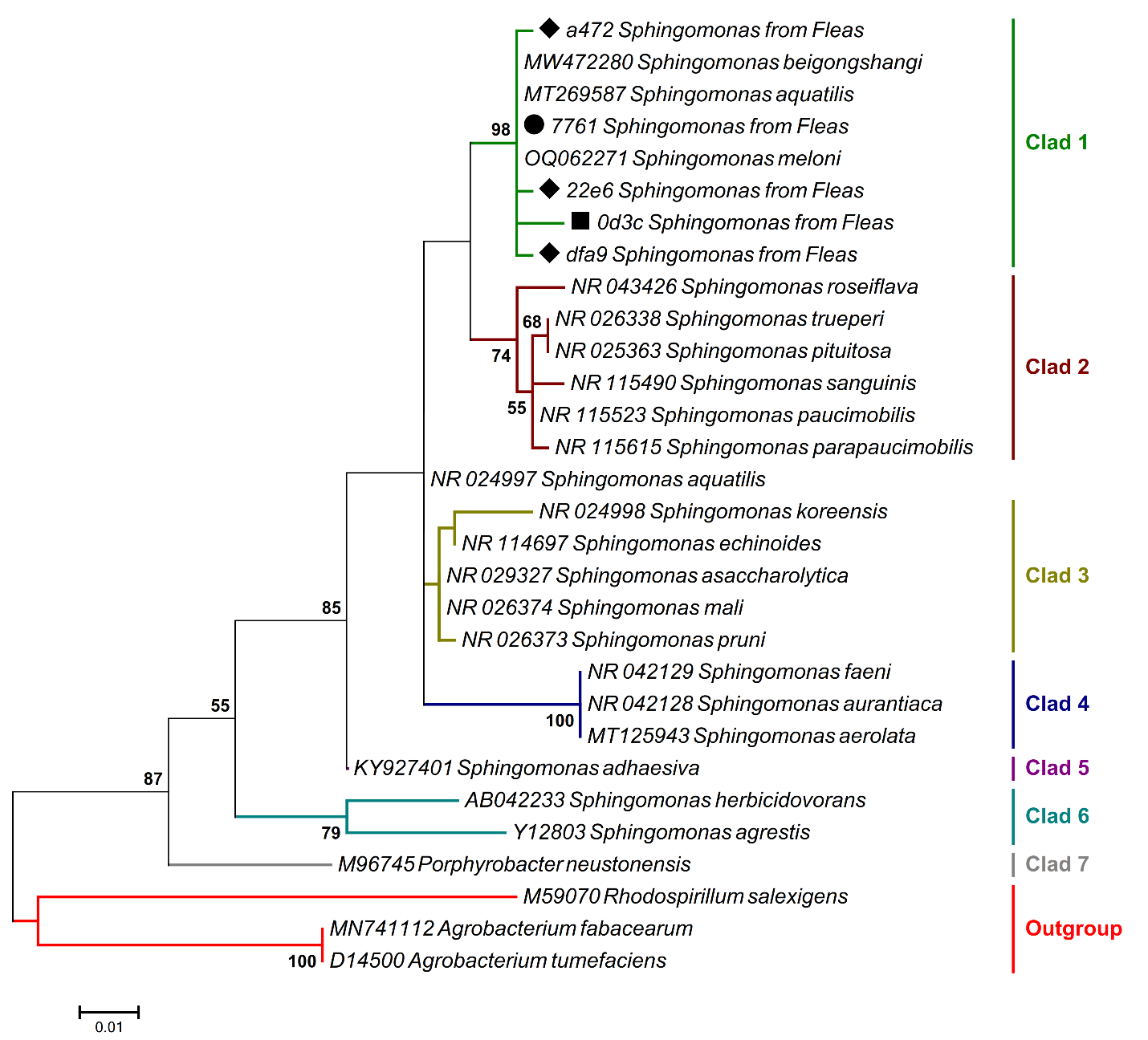


**Figure S9.** Maximum likelihood tree inferred from 404 bp of the *16S rRNA* gene sequences showing the position of 5 *Sphingomonas* isolates obtained in this study among 22 other *Sphingomonas* sequences belonging various strains reported in literature. Only representative sequences higher than 50 reads was included in the analysis. Diamond: CRBS; Circle: all fleas specimens excluding XNMS and square: NIMA. The sequences of *Agrobacterium* spp. (MN741112, and D14500) and *Rhodospirillum* sp. (M59070) were set as outgroup. Only bootstrap values higher than 50% are shown on the branches. The bar indicates substitutions per site.


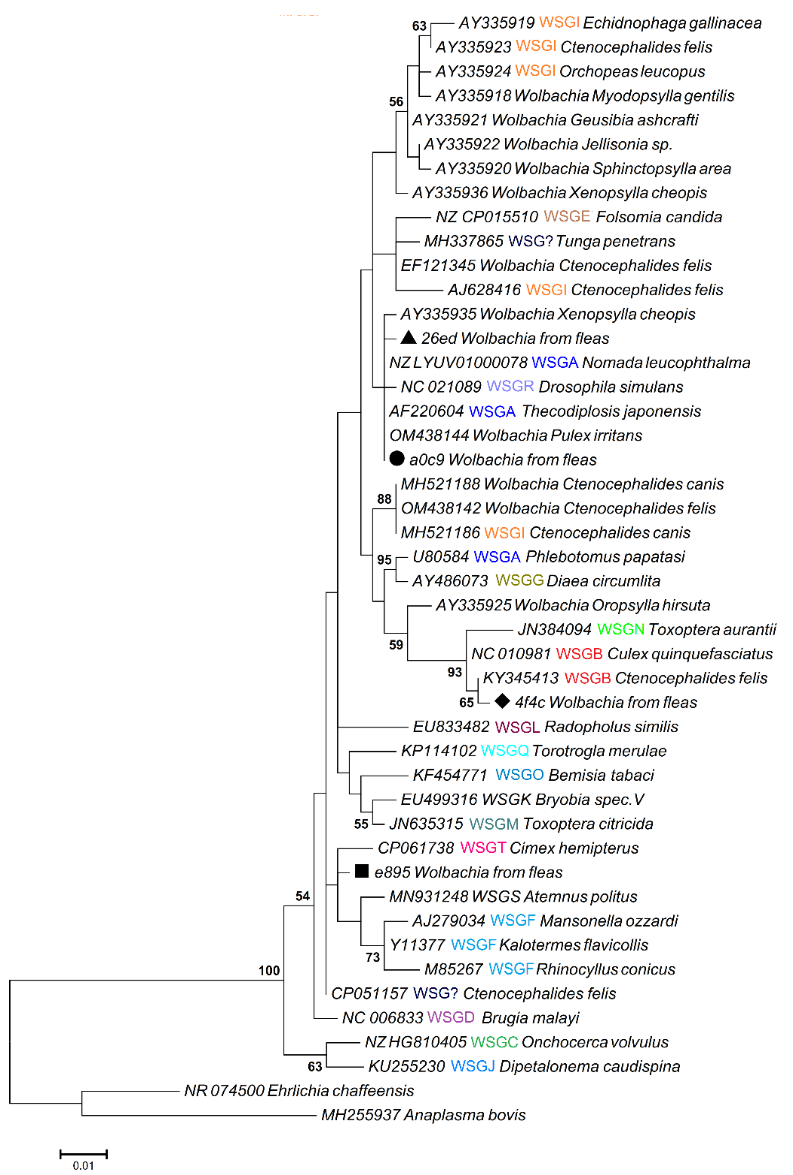


**Figure S10.** Maximum likelihood tree inferred from 404 bp of the *16S rRNA* gene sequences showing the position of 4 *Wolbachia* isolates obtained in this study among 40 other *Wolbachia* sequences belonging to the various super groups reported in literature. Only representative sequences higher than 50 reads was included in the analysis. Square: All fleas specimens; circle: all fleas specimens excluding NIFS and XNMS; tringle: NIMA, PIFA, PIMA and XBFT; and diamond: all specimens excluding XNMS. The sequences of *Ehrlichia* chaffeensis (NR074500) and *Anaplasma bovis* (MH255937) were set as outgroup. Only bootstrap values higher than 50% are shown on the branches. The bar indicates substitutions per site.


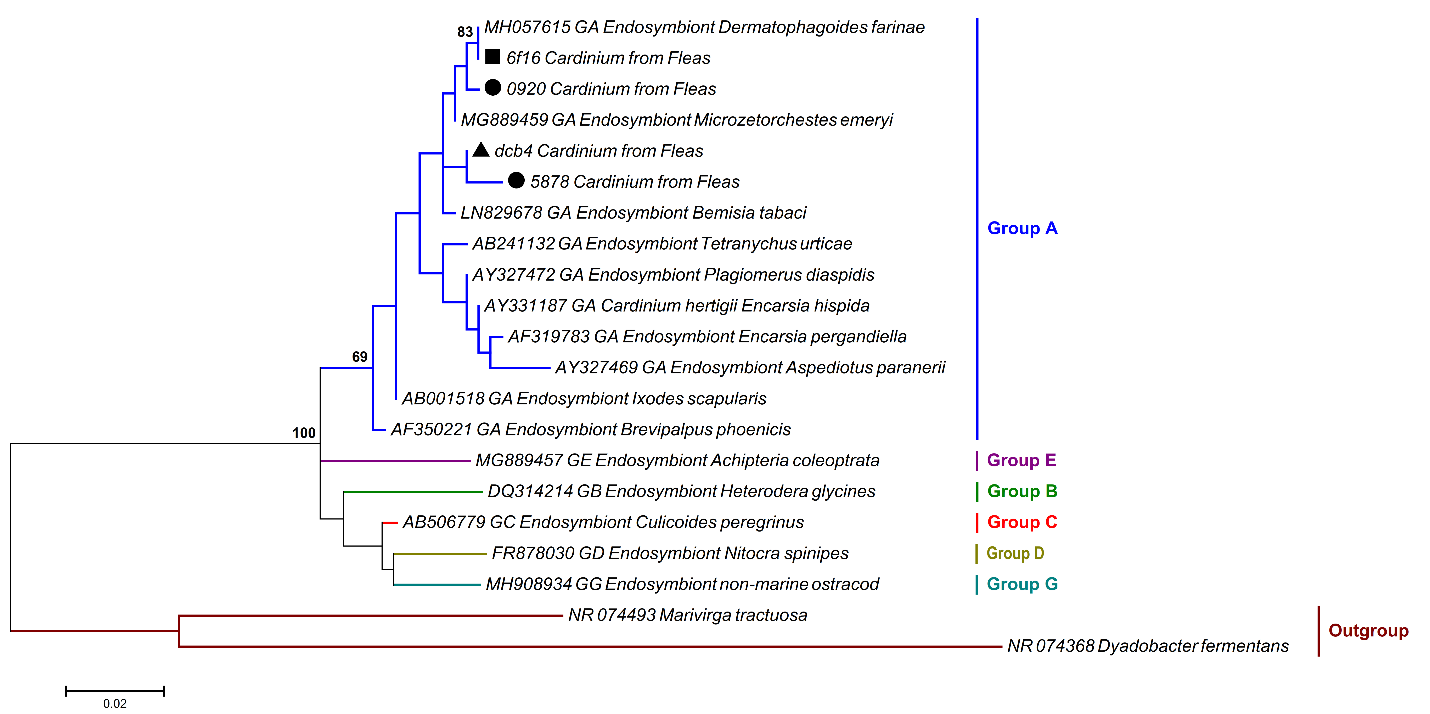


**Figure S11.** Maximum likelihood tree inferred from 404 bp of the *16S rRNA* gene sequences showing the position of 4 *Cardinium* sp. obtained in this study among 15 other *Cardinium* sequences related to the groups A-G reported in literature. Only representative sequences higher than 50 reads was included in the analysis. Square: all fleas specimens excluding NIMA, PIFA, PIMA and XBFT; circle: all fleas specimens excluding NIMA, PIFA, PIMA, XBFT, and XNMS and triangle: PIFA, XBFB, XBFS and XBFT. The sequences of *Marivirge tractuose* (NR074493) and *Dyadobacter fermentans* (NR074368) were set as outgroup. Only bootstrap values higher than 50% are shown on the branches. The bar indicates substitutions per site.


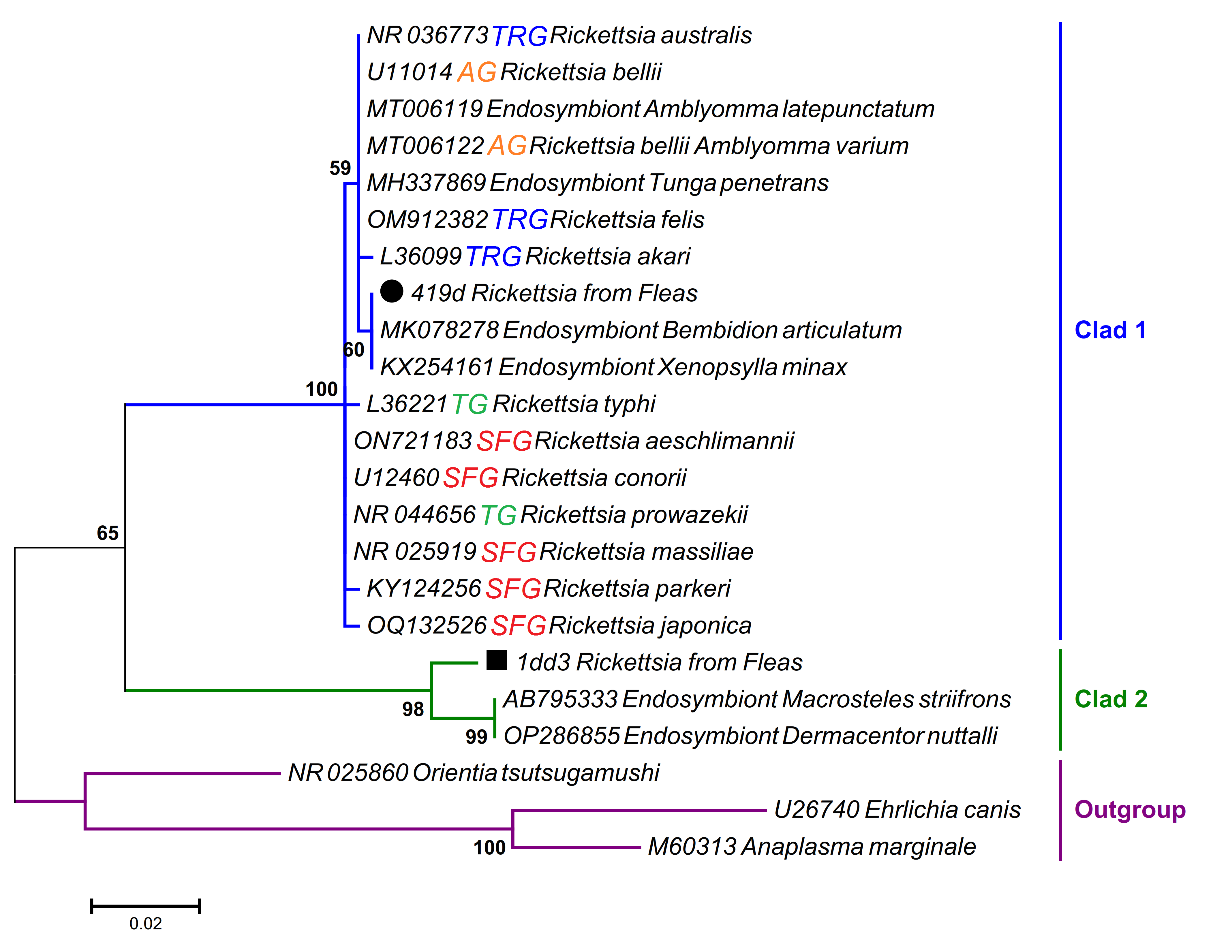


**Figure S 12.** Maximum likelihood tree inferred from 404 bp of the *16S rRNA* gene sequences showing the position of 2 *Rickettsia* sp. obtained in this study among 18 symbiotic/pathogenic *Rickettsial* species reported in literature. Only representative sequences higher than 50 reads was included in the analysis. Circle: all fleas specimens excluding XNMS and square: NIMA, PIFA, PIMA and XBFT. The sequences of *Orientia tsutsugamushis* (NR025860), *Ehrlichia canis* (U26740) and *Anaplasma marginale* (M60313) were set as outgroup. Only bootstrap values higher than 50% are shown on the branches. The bar indicates substitutions per site.


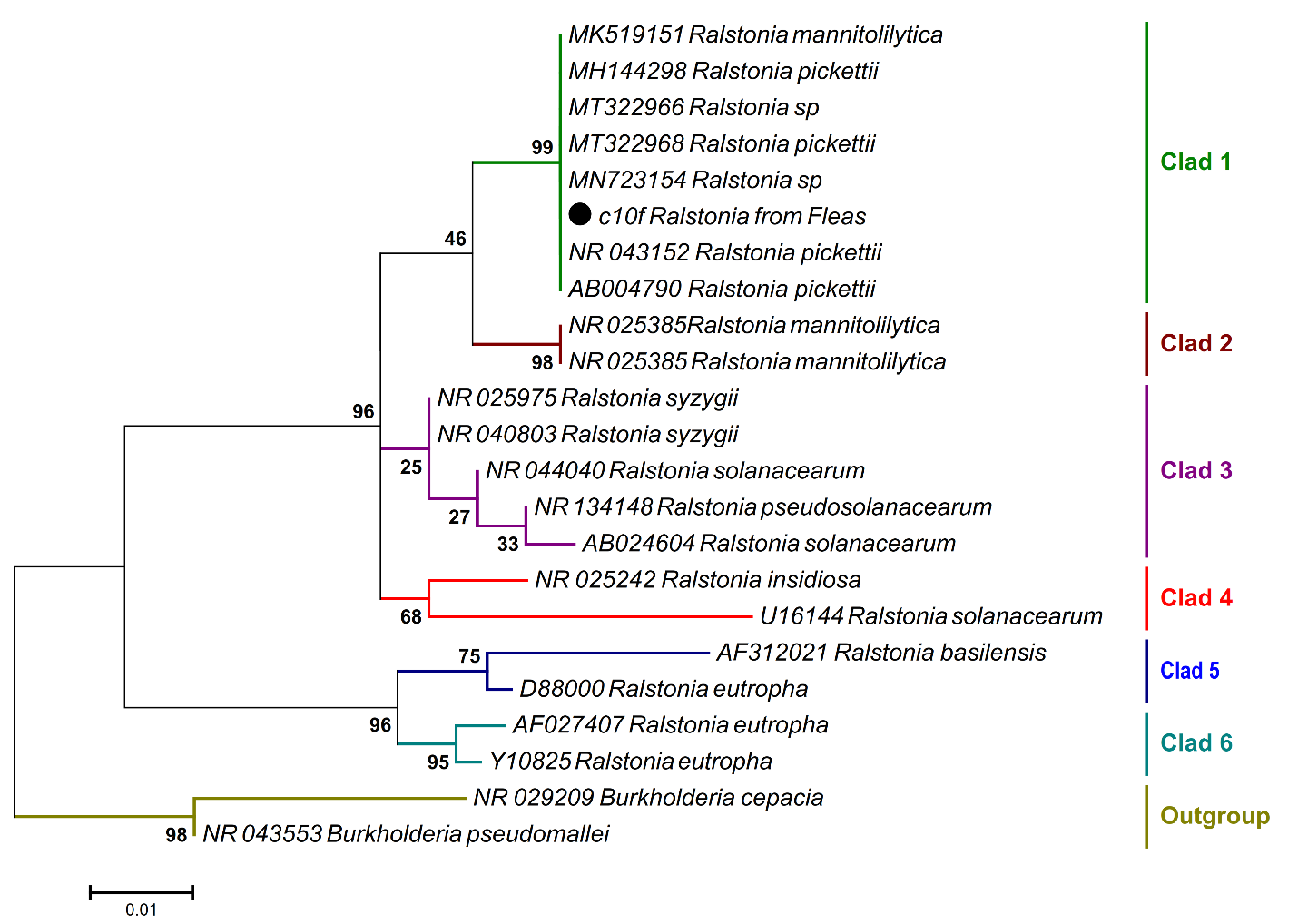


**Figure S13.** Maximum likelihood tree inferred from 404 bp of the *16S rRNA* gene sequences showing the position of a *Ralstonia* sp. obtained in this study among 20 other *Ralstonia* sequences reported in literature. Only representative sequences higher than 50 reads was included in the analysis. Sequences indicated by a circle, represents the putative bacterial sequences found in all fleas specimens except XNMS. The sequences of *Burkholderia* spp. (NR029209 and NR043553) and *Dyadobacter fermentans* (NR074368) were set as outgroups. Only bootstrap values higher than 50% are shown on the branches. The bar indicates substitutions per site**.**

**Table S1.**  Details of collected fleas specimens including species, locations, and hosts as well as the number of individuals used for NGS analyze per location.

| **Location** | **Longitude, latitude** | **Vertebrate host** | | | **Fleas species: (Male/Female), [Specimens used for NGS]** | | | | |  |
| --- | --- | --- | --- | --- | --- | --- | --- | --- | --- | --- |
|  |  | ***Meriones persicus*** | ***Microtus arvalis*** | ***Vulpes vulpes*** | ***Xenopsylla buxtoni*** | ***Xenopsylla nuttalli*** | ***Pulex irritans*** | ***Nosopsyllus iranus iranus*** | ***Ctenophthalmus rettigi smiti*** | **Total fleas** |
| Hamedan, Akanlu: Qeytar Mezruk | X:246019.345  Y:3952635.321 | 1 | 1 | - | 9(4,5)  [0,1] | - | - | - | - | 9 (4,5)  [0,1] |
| Hamedan, Akanlu: Kallik | X:234851.179  Y:3937096.595 | 6 | - | - | 74 (21,53)  [0,1] | - | - | - | - | 74 (21,53)  [0,1] |
| Hamedan, Akanlu: Qeytar Mezruk | X: 226010.006  Y:3944767.661 | 6 | - | - | 35(16,19)  [0,1] | - | - | - | - | 35(16,19)  [0,1] |
| Hamedan, Akanlu: Qara Gol | X:237619.058  Y:3948890.92 | - | 1 | - | 0(0,0)  [0,0] | - | - | - | - | 0(0,0)  [0,0] |
| Hamedan, Akanlu: Bashqurtaran | X:238272.572  Y:3948510.77 | 2 | - | - | 4(2,2)  [0,1] | - | - | - | - | 4 (2,2)  [0,1] |
| Hamedan, Akanlu: Akanlu | X:235582.616  Y:3938978.168 | - | - | 1 | 0(0,0)  [0,0] | - | 54(22,32)  [12,12] | - | - | 54 (22,32)  [12,12] |
| Hamedan, Akanlu: Bashqurtaran | X:236990.806  Y:3948761.417 | 1 | - | - | 9(4,5)  [0,1] | - | - | - | - | 9 (4,5)  [0,1] |
| Hamedan, Akanlu: Bashqurtaran | X: 237006.424  Y:3947416.009 | 2 | 1 | - | 14 (7,7)  [0,1] | - | - | - | - | 14 (7,7)  [0,1] |
| Hamedan, Akanlu: Kallik | X:234315.573  Y:3939015.808 | 3 | - | - | 27(16,11)  [0,1] | - | - | - | - | 27(16,11)  [0,1] |
| Hamedan, Akanlu: Kallik | X:236852.221  Y:3936299.836 | 3 | - | - | 5(2,3)  [0,1] | - | - | - | - | 5 (2,3)  [0,1] |
| Hamedan, Akanlu: Yekeh Chalab Klik | X:236825.672  Y:3936340.103 | 4 | - | - | 60(29,31)  [1,1] | - | - | - | - | 60 (29,31)  [1,1] |
| Hamedan, Akanlu: Kallik | X:236682.151  Y:3936346.799 | 3 | - | - | 26(16,10)  [0,1] | - | - | - | - | 26(16,10)  [0,1] |
| Hamedan, Akanlu: Pir Badam | X:226978.435  Y:3945925.674 | 1 | - | - | 4(2,2)  [0,1] | - | - | - | - | 4 (2,2)  [0,1] |
| Hamedan, Akanlu: Kohneh Hesar | X:233574.299  Y:3937396.818 | 11 | - | - | 25(11,14)  [0,1] | - | - | - | - | 25(11,14)  [0,1] |
| Hamedan, Akanlu: Bashqurtaran | X:238565.079  Y:3948354.083 | 2 | - | - | 24(12,12)  [1,0] | - | - | - | - | 24 (12,12)  [1,0] |
| Hamedan, Akanlu: Bashqurtaran | X:236467.097  Y:3946466.425 | 6 | - | - | 20 (8,12)  [1,0] | - | - | - | - | 20 (8,12)  [1,0] |
| Hamedan, Akanlu: Kohneh Hesar | X:229769.675  Y:3938402.721 | 3 | - | - | 1(1,0)  [1,0] | - | - | - | - | 1(1,0)  [1,0] |
| Hamedan, Akanlu: Yekeh Chalab Klik | X:234315.573  Y:3939015.808 | 1 | - | - | 7(4,3)  [1,0] | - | - | - | - | 7 (4,3)  [1,0] |
| Hamedan, Akanlu: Bashqurtaran | X:237437.095  Y:3947588.371 | 1 | - | - | 0(0,0)  [00] | - | - | - | - | 0(0,0)  [0,0] |
| Hamedan, Akanlu: Bashqurtaran | X:236200.382  Y:3946135.02 | 2 | - | - | 10 (3,7)  [1,0] | - | - | - | - | 10 (3,7)  [1,0] |
| Hamedan, Akanlu: Bashqurtaran | X:234762.338  Y:3946054.364 | 1 | - | - | 2(2,0)  [1,0] | - | - | - | - | 2 (2,0)  [1,0] |
| Hamedan, Akanlu: Bashqurtaran | X:236988.381  Y:3948764.57 | 1 | - | - | 0(0,0)  [0,0] | - | - | - | - | 0(0,0)  [0,0] |
| Hamedan, Akanlu: Bashqurtaran | X:237041.776  Y:3948611.849 | 1 | - | - | 0(0,0)  [0,0] | - | - | - | - | 0(0,0)  [0,0] |
| Hamedan, Akanlu: Bashqurtaran | X:236958.32  Y:3948429.235 | 2 | - | - | 67(38,29)  [1,0] | - | - | - | - | 67(38,29)  [1,0] |
| Hamedan, Akanlu: Dali Chu | X:228040.351  Y:3944017.172 | 3 | - | - | 20(10,10)  [1,0] | - | - | 8(5,3)  [5,0] | - | 28 (15,13)  [6,0] |
| Hamedan, Akanlu: Dali Chu | X:228065.871  Y:3944027.805 | 4 | - | - | 35(14,21)  [1,0] | - | - | 7(5,2)  [5,0] | - | 42 (19,23)  [6,0] |
| Hamedan, Akanlu: Dali Chu | X:233341.276  Y:3950070.11 | 2 | - | - | 3(2,1)  [1,0] | - | - | 4(2,2)  [2,0] | - | 7 (4,3)  [3,0] |
| Hamedan, Akanlu: Chopoqly | X:233220.132  Y:3950227.999 | 1 | - | - | 0(0,0)  [0,0] | - | - | - | - | 0(0,0)  [0,0] |
| **Sum** |  | 73 | 3 | 1 | 481(224,257)  [12,12] | 0 | 54(22,32)  [12,12] | 19(12,7)  [12,0] | 0 | 554(258,296)  [36,24] |
| West Azerbaijan, Seyed-Abad: A | X:597533.791  Y:4036973.256 | 3 | - | - | 4(2,2)  [1,1] | - | - | - | - | 4 (2,2)  [1,1] |
| West Azerbaijan, Seyed-Abad: B | X:597853.689  Y:4036637.874 | 4 | - | - | 35(15,20)  [1,1] | - | - | - | - | 35(15,20)  [1,1] |
| West Azerbaijan, Seyed-Abad: C | X:598240.075  Y:4036367.971 | 2 | - | - | 7(3,4)  [1,1] | - | - | - | - | 7(3,4)  [1,1] |
| West Azerbaijan, Seyed-Abad: D | X:596885.64  Y:4035511.363 | 5 | - | - | 38(16,22)  [1,1] | - | - | - | - | 38(16,22)  [1,1] |
| West Azerbaijan, Seyed-Abad: E | X:597423.958  Y:4036780.932 | 7 | - | - | 14(6,8)  [1,1] | - | - | - | - | 14 (6,8)  [1,1] |
| West Azerbaijan, Seyed-Abad: F | X:597627.269  Y:4036410.345 | 7 | - | - | 39 (17,22)  [1,1] | - | - | - | - | 39 (17,22)  [1,1] |
| West Azerbaijan, Shahrikand: A | X:597461.899  Y:4036947.77 | 1 | - | - | 2(1,1)  [0,0] | - | - | - | - | 2 (1,1)  [0,0] |
| West Azerbaijan, Shahrikand: B | X:598125.117  Y:4035312.699 | 2 | - | - | 30(12,18)  [1,1] | - | - | - | - | 30 (12,18)  [1,1] |
| West Azerbaijan, Shahrikand: C | X:598084.477  Y:4036699.049 | 4 | - | - | 28(14,14)  [1,1] | - | - | - | - | 28 (14,14)  [1,1] |
| West Azerbaijan, Shahrikand: D | X:598105.399  Y:4036391.115 | 1 | - | - | 29(14,15)  [1,1] | - | - | - | - | 29 (14,15)  [1,1] |
| West Azerbaijan, Shahrikand: E | X:597348.949  Y:4036810.916 | 5 | - | - | 62(34,28)  [1,1] | - | - | - | - | 62 (34,28)  [1,1] |
| West Azerbaijan, Seyed-Abad: G | X:597509.6  Y:4036911.349 | 2 | - | - | 3(1,2)  [0,0] | - | - | - | - | 3 (1,2)  [0,0] |
| West Azerbaijan, Seyed-Abad: H | X:597878.228  Y:4036668.956 | 1 | - | - | 8(3,5)  [0,1] | - | - | - | - | 8 (3,5)  [0,1] |
| West Azerbaijan, Seyed-Abad: I | X:598483.777  Y:4037261.383 | 3 | - | - | 7(3,4)  [1,0] | - | - | - | - | 7 (3,4)  [1,0] |
| West Azerbaijan, Seyed-Abad: J | X:598331.374  Y:4037530.858 | 7 | - | - | 11(4,7)  [1,1] | - | - | - | - | 11 (4,7)  [1,1] |
| **Sum** |  | 54 | 0 | 0 | 317(145,172)  [12,12] | 0 | 0 | 0 | 0 | 317(145,172)  [12,12] |
| East Azerbaijan, Sarab: Razliq | X:723051.738  Y:4213435.56 | 4 | - | - | - | - | - | 1(0,1)  [0,1] | - | 1 (0,1)  [0,1] |
| East Azerbaijan, Sarab: Khatunabad | X:711340.18  Y:4202438.117 | 1 | - | - | - | 5(3,2)  [1,1] | - | - | - | 5 (3,2)  [1,1] |
| East Azerbaijan, Sarab: Khatunabad | X:711360.507  Y:4202123.989 | 2 | - | - | - | 12(8,4)  [1,1] | - | - | - | 12(8,4)  [1,1] |
| East Azerbaijan, Sarab: Cherlu | X:711316.644  Y:4202119.776 | 5 | - | - | - | 61(26,35)  [1,1] | - | - | - | 61(26,35)  [1,1] |
| East Azerbaijan, Sarab: Idehlu | X:711212.545  Y:4202083.147 | 1 | - | - | 3(1,2)  [0,1] | - | - | - | - | 3(1,2)  [0,1] |
| East Azerbaijan, Sarab: Idehlu | X:711383.423  Y:4200101.012 | - | 6 | - | - | - | - | - | 2(0,2)  [0,1] | 2 (0,2)  [0,1] |
| East Azerbaijan, Sarab: Idehlu | X:711340.18  Y:4202438.117 | 4 | 2 | - | - | 1(0,1)  [0,1] | - | 2(0,2)  [0,2] | 1(1,0)  [1,0] | 4 (0,4)  [1,3] |
| East Azerbaijan-Idehlu | X:711026.901  Y:4202840.29 | 5 | - | - | 63(25,38)  [0,2] | - | - | 9(4,5)  [0,5] | - | 72(29,43)  [0,7] |
| East Azerbaijan, Sarab: Khatunabad | X:721136.369  Y:4210828.997 | 3 | - | - | - | 12 (6,6)  [2,1] | - | - | - | 12 (6,6)  [2,1] |
| East Azerbaijan, Sarab: Khatunabad | X:711577.412  Y:4200726.059 | 1 | - | - | - | 12(5,7)  [2,2] | - | - | - | 12 (5,7)  [2,2] |
| East Azerbaijan, Sarab: Khatunabad | X:711577.412  Y:4200726.059 | 1 | - | - | - | 1(0,1)  [0,1] | - | - | - | 1(0,1)  [0,1] |
| East Azerbaijan, Sarab: Idehlu | X:753470.07  Y:3985539.411 | 1 | - | - | 0(0,0)  [0,0] | - | - | - | - | 0(0,0)  [0,0] |
| East Azerbaijan, Sarab: Idehlu | X:756612.538  Y:3972076.337 | 6 | - | - | 54(17,37)  [0,3] | - | - | - | - | 54 (17,37)  [0,3] |
| East Azerbaijan, Sarab: Idehlu | X:748953.131  Y:4209858.374 | 8 | 1 | - | 3 (0,3)  [0,3] | - |  | 4(0,4)  [0,3] | - | 7 (0,7)  [0,6] |
| East Azerbaijan, Sarab: Idehlu | X:748985.542  Y:4208795.364 | 7 | - | - | 49(19,30)  [0,2] | 23(9,14)  [1,1] | - | - | - | 72 (26,46)  [1,3] |
| East Azerbaijan, Sarab: Cherlu | X:710780.925  Y:4200440.234 | 1 | - | - | - | 26(12,14)  [1,1] | - | - | - | 26 (12,14)  [1,1] |
| East Azerbaijan, Sarab: Cherlu | X:712947.266  Y:4199913.267 | 1 | - | - | - | - | - | 1(0,1)  [0,1] | - | 1 (0,1)  [0,1] |
| East Azerbaijan, Sarab: Cherlu | X:710831.628  Y:4199324.888 | 2 | - | - | - | 10(4,6)  [2,1] | - | - | - | 10 (4,6)  [2,1] |
| East Azerbaijan, Sarab: Idehlu | X:742354.297  Y:4204269.917 | 1 | - | - | - | 8(4,4)  [1,1] | - | - | - | 8 (4,4)  [1,1] |
| **Sum** |  | 54 | 7 | 0 | 172(62,110)  [0,12] | 171(77,94)  [12,12] | 0 | 17(4,13)  [0,12] | 3(1,2)  [1,1] | 363(144,219)  [13,37] |
| Tehran, Tello: A | X:556324.002  Y:3961538.428 | 1 | - | - | 7 (2,5)  [0,1] | - | - | - | - | 7 (2,5)  [0,1] |
| Tehran, Tello: B | X:556533.918  Y:3959718.867 | 1 | - | - | 23(10,13)  [0,2] | - | - | - | - | 23 (10,13)  [0,2] |
| Tehran, Tello: C | X:553970.14  Y:3962260.12 | 4 | - | - | 24(12,12)  [0,2] | - | - | - | - | 24 (12,12)  [0,2] |
| Tehran, Tello: D | X:556465.018  Y:3959499.669 | 7 | - | - | 47(17,30)  [0,2] | - | - | - | - | 47 (17,30)  [0,2] |
| Tehran, Tello: E | X:556693.409  Y:3959904.746 | 15 | 2 | - | 98(32,66)  [0,5] | - | - | 4(2,2)  [0,0] | - | 102(32,68)  [0,5] |
| **Sum** |  | 28 | 2 | 0 | 199(73,126)  [0,12] | 0 | 0 | 4(2,2)  [0,0] | 0 | 203 (75,128)  [0,12] |
| **Total** |  | **209** | **12** | **1** | **1169(504,665)**  **[24,48]** | **171(77,94)**  **[12,12]** | **54(22,32)**  **[12,12]** | **40(18,22)**  **[12,12]** | **3(1,2)**  **[1,1]** | **1437 (622,815)**  **[61,85]** |

**Table S2.** Statistics for raw and refined sequences obtained in this study.

| **Sample id** | **input numeric** | **filtered numeric** | **Percentage of input passed filter numeric** | **denoised numeric** | **merged numeric** | **Percentage of input merged numeric** | **non-chimeric numeric** | **Percentage of input non-chimeric numeric** |
| --- | --- | --- | --- | --- | --- | --- | --- | --- |
| **CRBS** | 105964 | 99024 | 93.45 | 98851 | 98409 | 92.87 | 98084 | 92.56 |
| **NIFS** | 135991 | 126712 | 93.18 | 126477 | 126007 | 92.66 | 123734 | 90.99 |
| **NIMA** | 147788 | 136050 | 92.06 | 135519 | 133925 | 90.62 | 127622 | 86.35 |
| **PIFA** | 135450 | 122828 | 90.68 | 122678 | 121616 | 89.79 | 117131 | 86.48 |
| **PIMA** | 142243 | 130469 | 91.72 | 130201 | 129364 | 90.95 | 126475 | 88.91 |
| **XBFA** | 129124 | 120289 | 93.16 | 120193 | 119167 | 92.29 | 118536 | 91.8 |
| **XBFB** | 134027 | 123377 | 92.05 | 123252 | 121326 | 90.52 | 120247 | 89.72 |
| **XBFS** | 148024 | 137199 | 92.69 | 137060 | 135974 | 91.86 | 133952 | 90.49 |
| **XBFT** | 135120 | 123773 | 91.6 | 123357 | 122034 | 90.32 | 114879 | 85.02 |
| **XBMA** | 132079 | 122605 | 92.83 | 122106 | 120668 | 91.36 | 117796 | 89.19 |
| **XBMB** | 134262 | 124096 | 92.43 | 123797 | 122508 | 91.25 | 120635 | 89.85 |
| **XNFS** | 133258 | 122471 | 91.91 | 122289 | 121539 | 91.21 | 119699 | 89.83 |
| **XNMS** | 68528 | 51308 | 74.87 | 50232 | 43715 | 63.79 | 43706 | 63.78 |
| **Total** | 1681858 | 1540201 | --- | 1536012 | 1516252 | --- | 1482496 | --- |

**Table S3** The mean alpha diversity for flea microbiome determined by four (Shannon, observed features, Faith, and Pielou) indices based on species, location, and sex.

|  | Species | | | | | Location | | | | Gender | | |
| --- | --- | --- | --- | --- | --- | --- | --- | --- | --- | --- | --- | --- |
| Indices | CRS | NII | PI | XB | XN | Akanlu | Bukan | Sarab | Tello | female | male | f/m |
| Shannon entropy | 1.64 | **2.89** | **1.57** | 2.40 | 1.97 | 2.40 | 2.28 | **1.84** | **3.10** | **2.15** | **2.44** | 1.64 |
| observed features | 113 | **236** | **82** | 89 | 48 | 144 | 92 | **76** | **98** | **75** | **148** | 113 |
| Faith's PD | 11 | **25** | **14** | 16 | 8 | 21 | 13 | **10** | **22** | **13** | **20** | 11 |
| Pielou evenness | 0.24 | **0.37** | **0.25** | 0.37 | 0.35 | 0.34 | 0.35 | **0.31** | **0.47** | **0.34** | **0.35** | 0.24 |

* The most and the least complex microbiota are highlighted and underlined respectively.

**Table S4.** Details of the number of unique bacterial genera in each flea species studied.

| **Flea species** | **Number of bacterial genera** | **Number of unique bacterial genera** |
| --- | --- | --- |
| *Ctenophthalmus rettigi smiti* | 51 | 51 |
| *Nosopsyllus iranus iranus* | 129 | 129 |
| *Pulex irritans* | 71 | 71 |
| *Xenopsylla buxtoni* | 131 | 131 |
| *Xenopsylla nuttalli* | 46 | 46 |
| **Overall number of unique bacterial genera** | | **230** |

**Table S5.** Details of the bacterial genera shared between the studied flea species.

| **Flea species** | **Total** | **Bacterial genera** |
| --- | --- | --- |
| *Ctenophthalmus rettigi smiti Nosopsyllus iranus iranus Pulex irritans Xenopsylla buxtoni Xenopsylla nuttalli* | 22 | Streptococcus, Lawsonella, Micrococcus, Pseudomonas, Corynebacterium, Acinetobacter, Ralstonia, Staphylococcus, Enhydrobacter, Cardinium, Cutibacterium, Bartonella ,Haemophilus, Bacillus, Rickettsia, Sphingomonas, Actinomyces, Serratia, Kocuria, Wolbachia, Methylobacterium-Methylorubrum, Lactobacillus |
| *Ctenophthalmus rettigi smiti Nosopsyllus iranus iranus Pulex irritans Xenopsylla buxtoni* | 2 | Brevibacillus, Rothia |
| *Ctenophthalmus rettigi smiti Nosopsyllus iranus iranus Pulex irritans Xenopsylla nuttalli* | 1 | Stenotrophomonas |
| *Ctenophthalmus rettigi smiti Pulex irritans Xenopsylla buxtoni Xenopsylla nuttalli* | 1 | Gemella |
| *Nosopsyllus iranus iranus Pulex irritans Xenopsylla buxtoni Xenopsylla nuttalli* | 4 | Actinotignum, Nocardioides, Bacteroides, Enterobacter |
| *Ctenophthalmus rettigi smiti Nosopsyllus iranus iranus Pulex irritans* | 2 | Veillonella, Prevotella |
| *Ctenophthalmus rettigi smiti Nosopsyllus iranus iranus Xenopsylla buxtoni* | 4 | Anaerococcus, Arthrobacter, Peptoniphilus, Pantoea |
| *Ctenophthalmus rettigi smiti Pulex irritans Xenopsylla buxtoni* | 3 | Neisseria, Granulicatella, Proteiniphilum |
| *Ctenophthalmus rettigi smiti Xenopsylla buxtoni Xenopsylla nuttalli* | 1 | Fusobacterium |
| *Nosopsyllus iranus iranus Pulex irritans Xenopsylla buxtoni* | 10 | Psychrobacter, Brachybacterium, Brevibacterium, Massilia, Paenibacillus, Rubellimicrobium Fictibacillus Uncultured rickettsiaceae Paracoccus Aeromonas |
| *Nosopsyllus iranus iranus Pulex irritans Xenopsylla nuttalli* | 1 | Alloprevotella |
| *Nosopsyllus iranus iranus Xenopsylla buxtoni Xenopsylla nuttalli* | 4 | Brevundimonas, Mycobacterium, Pelomonas, Moraxella |
| *Ctenophthalmus rettigi smiti Nosopsyllus iranus iranus* | 2 | Myroides, Faecalibacterium |
| *Ctenophthalmus rettigi smiti Pulex irritans* | 1 | Cellulomonas |
| *Ctenophthalmus rettigi smiti Xenopsylla buxtoni* | 3 | Dermacoccus, Agathobacter ,Herbaspirillum |
| *Ctenophthalmus rettigi smiti Xenopsylla nuttalli* | 1 | Parvimonas |
| *Nosopsyllus iranus iranus Pulex irritans* | 6 | Blastococcus, Holdemanella, Erysipelothrix, Flavobacterium, Abiotrophia, Actinobacillus |
| *Nosopsyllus iranus iranus Xenopsylla buxtoni* | 17 | Romboutsia, Umezawaea, Enterococcus, Microbacterium, Finegoldia, Noviherbaspirillum, Lautropia, Escherichia, Dietzia, Skermanella, Bifidobacterium, Streptomyces, Colidextribacter, Campylobacter, Ruminococcus, Phenylobacterium, Cellulosilyticum |
| *Nosopsyllus iranus iranus Xenopsylla nuttalli* | 1 | Aquabacterium |
| *Pulex irritans Xenopsylla buxtoni* | 3 | Limnobacter, Amaricoccus, Brumimicrobium |
| *Pulex irritans Xenopsylla nuttalli* | 1 | Asinibacterium |
| *Xenopsylla buxtoni Xenopsylla nuttalli* | 1 | Oscillibacter |
| *Ctenophthalmus rettigi smiti* | 8 | Sphingobacterium, Mycoplasma, Fenollaria, Pseudopropionibacterium, Geminicoccus, Murdochiella, Cnuella, Mogibacterium |
| *Nosopsyllus iranus iranus* | 53 | Marinobacter, Oribacterium, Vagococcus, Rodentibacter, Francisella, Blastopirellula, Saccharopolyspora, Ruminiclostridium, Erwinia, Solirubrobacter, Rhodoplanes, Providencia, Dermabacter, Dokdonia, Kribbella, Cetobacterium, Leuconostoc, Rubrobacter, Promicromonospora, Sulfitobacter, Chishuiella, Lysinibacillus, Comamonas, Woeseia, Stackebrandtia, Ignatzschineria, Actinomadura, Lechevalieria, Abyssicoccus, Collinsella, Actinomycetospora, Pseudonocardia, Nonlabens, Dolosigranulum, Rhodobacter, Ramlibacter, Nocardia, Akkermansia, Lentimonas, Hymenobacter, Pseudarthrobacter, Sandaracinus, Megasphaera, Dechloromonas, Gordonia, Actinocorallia, Weissella, Lachnospira, Saccharothrix, Porphyromonas, Cloacibacterium, Dinghuibacter, Paraclostridium |
| *Pulex irritans* | 14 | Slackia, Dorea, Dialister, Macellibacteroides, Carnobacterium, Georgenia, Lachnoanaerobaculum, Rufibacter, Thermus, Paeniclostridium, Longispora, Shewanella, Cruoricaptor, Methylorosula |
| *Xenopsylla buxtoni* | 56 | Aureimonas, Zoogloea, Haliangium, Enteractinococcus, Hydrogenophaga, Fastidiosipila, Geobacillus, Pedobacter, Alloiococcus, Propioniciclava, Nesterenkonia, Eremococcus, Babeliales, Saccharimonas, Schlegelella, Megamonas, Peredibacter, Microvirga, Adhaeribacter, Curtobacterium, Helicobacter, Porphyrobacter, Caulobacter, Subdoligranulum, Azospirillum, Quinella, Chryseobacterium, Bdellovibrio, Pseudolabrys, Alysiosphaera, Phascolarctobacterium, Terrisporobacter, Dubosiella, Tepidimonas, Acidovorax xylanophilum, Catonella, Haliscomenobacter, Nomurabacteria, Deinococcus, Mariniphaga, Phyllobacterium, Nitrospira, Acidocella, Actinophytocola, Helcococcus, Lactococcus, Actinotalea, Agrococcus, Ileibacterium, Tessaracoccus, Leptotrichia, Anoxybacillus, Hyphomicrobium, Alkanindiges, Rheinheimera |
| *Xenopsylla nuttalli* | 8 | Aurantisolimonas,, Modestobacter, Sphingobium, Anaerostipes, Novosphingobium, Blastomonas, Parabacteroides, Mucispirillum |

**Table S6** Details of the number of unique bacterial genera of fleas in each studied location.

| **Location** | **Number of bacterial genera** | **Number of unique bacterial genera** |
| --- | --- | --- |
| Akanlu | 161 | 161 |
| Bukan | 73 | 73 |
| Sarab | 105 | 105 |
| Tello | 38 | 38 |
| Overall number of bacterial genera | | 229 |

**Table S7** Details of the bacterial genera of fleas shared between the studied locations.

| **Location** | **Total** | **Bacterial genera** |
| --- | --- | --- |
| Akanlu Bukan Sarab Tello | 16 | Streptococcus, Pseudomonas, Psychrobacter, Corynebacterium, Acinetobacter, Staphylococcus, Ralstonia, Cardinium, Cutibacterium, Bartonella, Bacillus, Rickettsia, Sphingomonas, Nocardioides, Wolbachia, Methylobacterium-Methylorubrum |
| Akanlu Bukan Tello | 2 | Amaricoccus, Escherichia |
| Akanlu Sarab Tello | 6 | Mycobacterium, Brevibacillus, Haemophilus, Erysipelothrix, Rothia ,Uncultured Rickettsiaceae |
| Akanlu Bukan Sarab | 17 | Brevundimonas Lawsonella, Micrococcus, Enhydrobacter, Massilia, Rubellimicrobium, Gemella, Actinomyces, Neisseria, Granulicatella, Herbaspirillum, Serratia, Actinotignum ,Paracoccus, Enterobacter ,Lactobacillus, Proteiniphilum |
| Akanlu Tello | 4 | Umezawaea, Noviherbaspirillum, Alysiosphaera, Terrisporobacter |
| Bukan Tello | 3 | Dubosiella, Leptotrichia, Alkanindiges |
| Akanlu Bukan | 10 | Brachybacterium, Enterococcus, Finegoldia, Skermanella, Bifidobacterium, Streptomyces, Colidextribacter, Fictibacillus, Aeromonas, Rheinheimera |
| Akanlu Sarab | 26 | Romboutsia, Saccharopolyspora, Solirubrobacter, Providencia, Asinibacterium, Lautropia, Alloprevotella, Stenotrophomonas, Brumimicrobium, Oscillibacter, Anaerococcus, Arthrobacter ,Veillonella, Hymenobacter, Aquabacterium, Pelomonas, Peptoniphilus, Myroides, Moraxella, Kocuria, Prevotella, Bacteroides, Faecalibacterium, Cellulosilyticum, Cellulomonas, Pantoea |
| Bukan Sarab | 7 | Dermacoccus, Fastidiosipila, Agathobacte,r Fusobacterium, Pseudolabrys, Campylobacter, Phenylobacterium |
| Tello | 7 | Pedobacter, Alloiococcus, Caulobacter, Chryseobacterium, Acidovorax, Catonella, Phyllobacterium |
| Akanlu | 80 | Slackia, Marinobacter, Oribacterium, Vagococcus, Zoogloea, Dorea, Rodentibacter, Francisella, Blastopirellula, Limnobacter, Hydrogenophaga, Blastococcus, Geobacillus, Dermabacter, Dokdonia, Propioniciclava, Microbacterium, Kribbella, Leuconostoc, Sulfitobacter, Chishuiella, Lysinibacillu,s Comamonas, Babeliales, Woeseia, Stackebrandtia, Dialister, Ignatzschineria, Schlegelella, Peredibacter, Actinomadura, Adhaeribacter, Macellibacteroides, Dietzia, Brevibacterium, Lechevalieria, Helicobacter, Porphyrobacter, Holdemanella, Abyssicoccus, Pseudonocardia, Nonlabens, Dolosigranulum, Carnobacterium, Flavobacterium, Ramlibacter, Nocardia, Georgenia, Phascolarctobacterium, ,Paenibacillus, Akkermansia, Lentimonas, xylanophilum, Lachnoanaerobaculum, Sandaracinus, Haliscomenobacter, Abiotrophia, Rufibacter, Thermus, Paeniclostridium, Deinococcus, Megasphaera, Longispora, Shewanella, Gordonia ,Actinocorallia, Weissella, Helcococcus, Ruminococcus, Actinotalea, Lactococcus, Cruoricaptor, Ileibacterium, Tessaracoccus, Lachnospira, Saccharothrix, Methylorosula, Porphyromonas, Actinobacillus, Anoxybacillus |
| Bukan | 18 | Aureimonas, Haliangium, Enteractinococcus, Nesterenkonia, Eremococcus, Thermoactinomyces, Saccharimonas, Megamonas, Microvirga, Curtobacterium, Subdoligranulum, Azospirillum, Quinella, Tepidimonas, Nitrospira, Acidocella, Actinophytocola, Hyphomicrobium |
| Sarab | 33 | Aurantisolimonas, Modestobacter, Ruminiclostridium, Erwinia, Sphingobacterium, Rhodoplanes, Sphingobium ,Cetobacterium, Rubrobacter, Anaerostipes, Promicromonospora, Mycoplasma, Actinomycetospora, Fenollaria ,Rhodobacter, Novosphingobium, Bdellovibrio, Blastomonas, Pseudarthrobacter, Parvimonas, Pseudopropionibacterium, Geminicoccus, Mariniphaga, Dechloromonas, Parabacteroides, Murdochiella, Cnuella, Mogibacterium, Agrococcus, Cloacibacterium, Dinghuibacter, Mucispirillum Paraclostridium |

**Table S8.** Details of the phylotypes of 6 top bacteria found in this study. Each phylotype is identified by a four-letter code in the studied flea species.

|  | **CRBS** | **NIFS** | **NIMA** | **PIFA** | **PIMA** | **XBFA** | **XBMA** | **XBFB** | **XBMB** | **XNFS** | **XNMS** | **XBFS** | **XBFT** |
| --- | --- | --- | --- | --- | --- | --- | --- | --- | --- | --- | --- | --- | --- |
| *Bartonella* |  |  |  |  |  |  |  |  | e08a | e08a |  | e08a |  |
|  |  |  |  |  |  |  |  |  |  |  |  | eb47 |  |
|  |  |  |  |  |  |  |  |  |  |  |  | e2d9 |  |
|  | 9c98 | 9c98 | 9c98 | 9c98 | 9c98 | 9c98 | 9c98 | 9c98 | 9c98 | 9c98 | 9c98 | 9c98 | 9c98 |
|  | 1418 | 1418 | 1418 |  |  | 1418 | 1418 | 1418 | 1418 | 1418 | 1418 | 1418 | 1418 |
|  | 65c0 | 65c0 | 65c0 |  |  | 65c0 | 65c0 | 65c0 | 65c0 | 65c0 | 65c0 | 65c0 | 65c0 |
|  | dee2 | dee2 |  |  |  | dee2 | dee2 | dee2 | dee2 | dee2 | dee2 | dee2 | dee2 |
|  | c49e |  | c49e | c49e | c49e |  |  | c49e | c49e | c49e |  | c49e | c49e |
|  |  | fbde |  |  |  |  |  |  |  |  |  |  |  |
| *Sphingomonas* | a472 |  |  |  |  |  |  |  |  |  |  |  |  |
|  | 22e6 |  |  |  |  |  |  |  |  |  |  |  |  |
|  | dfa9 |  |  |  |  |  |  |  |  |  |  |  |  |
|  | 7761 | 7761 | 7761 | 7761 | 7761 | 7761 | 7761 | 7761 | 7761 | 7761 |  | 7761 | 7761 |
|  |  |  | 0d3c |  |  |  |  |  |  |  |  |  |  |
| *Wolbachia* | e895 | e895 | e895 | e895 | e895 | e895 | e895 | e895 | e895 | e895 | e895 | e895 | e895 |
|  | a0c9 |  | a0c9 | a0c9 | a0c9 | a0c9 | a0c9 | a0c9 | a0c9 | a0c9 |  | a0c9 | a0c9 |
|  |  |  | 26ed | 26ed | 26ed |  |  |  |  |  |  |  | 26ed |
|  | 4f4c | 4f4c | 4f4c | 4f4c | 4f4c | 4f4c | 4f4c | 4f4c | 4f4c | 4f4c |  | 4f4c | 4f4c |
| *Cardinium* | 6f16 | 6f16 |  |  |  | 6f16 | 6f16 | 6f16 | 6f16 | 6f16 | 6f16 | 6f16 |  |
|  | 5878 | 5878 |  |  |  | 5878 | 5878 | 5878 | 5878 | 5878 |  | 5878 |  |
|  | 0920 | 0920 |  |  |  | 0920 | 0920 | 0920 | 0920 | 0920 |  | 0920 |  |
|  |  |  |  | dcb4 |  |  |  | dcb4 |  |  |  | dcb4 | dcb4 |
| *Rickettsiia* | 419d | 419d | 419d | 419d | 419d | 419d | 419d | 419d | 419d | 419d |  | 419d | 419d |
|  |  |  | 1dd3 | 1dd3 | 1dd3 |  |  |  |  |  |  |  | 1dd3 |
| *Ralstonia* | c10f | c10f | c10f | c10f | c10f | c10f | c10f | c10f | c10f | c10f |  | c10f | c10f |

**Table S9.** Details of the bacterial species identified from flea species with isolation sources and their bio/ecological importance mentioned in the literature.

| **Species** | **Isolation source in current study** | | | | | | | | | | | | | **Functions of bacteria*** | **Representative isolation source in the literature** | **Bio/ecological significance** |
| --- | --- | --- | --- | --- | --- | --- | --- | --- | --- | --- | --- | --- | --- | --- | --- | --- |
|  | **CRBS** | **NIFS** | **NIMA** | **PIFA** | **PIMA** | **XBFA** | **XBMA** | **XBFB** | **XBMB** | **XNFS** | **XNMS** | **XBFS** | **XBFT** |  |  |  |
| *Acinetobacter rudis* |  |  | * |  |  |  |  |  |  |  |  |  |  | Environmental/ Opportunistic pathogens | Raw milk and wastewater [1] | a psychrophilic bacterium can propagate in raw milk stored in refrigerators [2] |
| *Acinetobacter schindleri* |  |  |  | * | * | * | * |  | * |  |  |  | * | Environmental/ Opportunistic pathogens | relatives are found in diverse environments, ranging from soil, water, and air to [3] human clinical samples [4] | ability to survive on inert surfaces for a long duration [3] |
| *Acinetobacter ursingii* |  | * | * |  | * |  |  |  | * |  |  |  | * | Opportunistic pathogens | Human clinical samples [3, 5] | mostly causes primary bacteremia in patients with malignancies [5] |
| *Actinocorallia longicatena* |  |  | * |  |  |  |  |  |  |  |  |  |  | environmental | leaf and root of herbaceous plant [6] | containing madurose as the diagnostic sugar [6] |
| *Actinomyces massiliensis* |  |  | * |  |  |  |  |  |  |  |  |  |  | pathogen | oral mucosa of humans and animals [7] | production of subgingival biofilms and the development of periodontitis [8] |
| *Anaerococcus octavius* |  |  |  |  |  |  | * |  |  |  |  |  |  | Opportunistic pathogens | Normal flora of the nose, skin, and vagina [9, 10]. | Fermentation of ribose, glucose, and mannose [10] |
| *Bacillus coagulans* |  | * |  |  |  |  |  |  |  |  |  |  |  | Probiotic | Products containing milk and carbohydrate [11] | high tolerance of extreme environments [11] |
| *Bacillus thermoamylovorans* |  |  | * |  |  |  |  |  |  |  |  |  |  | Environmental | Warm spring water [12]; Products containing milk [13] | production of lipolytic enzymes and β-galactosidase linked to food spoilage and dairy products [14] |
| *Bacteroides plebeius* |  |  |  |  |  |  | * |  |  |  |  |  |  | commensal | Human gut and feces [15]. | as a prebiotic may hydrolyze agar [16] |
| *Bacteroides vulgatus* |  | * |  |  |  |  |  |  |  |  |  |  |  | commensal | Human colon [17] | formation of biofilms on the surface of food particles in the colon [18] |
| *Bifidobacterium commune* |  |  | * |  |  |  |  |  |  |  |  |  |  | probiotic, symbiont | guts of bumble bees [19] | potentially beneficial effect on bee health [19] |
| *Brevibacterium pityocampae* |  |  |  |  |  |  | * |  |  |  |  |  |  | symbiont | caterpillars of *Thaumetopoea pityocampa* [20] | builds an aerial mycelium |
| *Campylobacter gracilis* |  |  |  |  |  |  |  |  | * |  |  |  |  | commensal | gingival crevice which may cause periodontal and pleuropulmonary infections [21, 22] | Formation of biofilms on different surfaces [23] |
| *Corynebacterium*  *jeikeium* |  |  | * |  |  |  |  |  |  |  |  |  |  | opportunistic pathogen mainly in bone marrow transplant patients | soil, water, and part of normal human skin flora [24] | a biofilm-forming non-diphtheria Corynebacterium [25] |
| *Corynebacterium amycolatum* |  | * | * |  |  |  |  |  |  |  |  |  |  | commensal | causing various infections, especially in immunocompromised people [26] | multidrug resistance isolates with the ability to produce biofilm [25] |
| *Corynebacterium appendicis* | * |  |  |  |  | * |  |  |  |  |  |  |  | pathogen | human clinical specimen, mainly appendicitis [27] | a lipophilic coryneform [27] |
| *Corynebacterium imitans* |  |  |  |  |  |  |  | * |  | * |  |  |  | pathogen | nasopharynx of suspected diphtheria patients [28] | potential to produce biofilms in patients [25] |
| *Corynebacteriumdurum* |  |  | * |  |  |  |  |  |  |  |  |  |  | commensal | human digestive and respiratory tracts [29] | maintaining systematic homeostasis via metabolite [30] |
| *Cruoricaptor ignavus* |  |  |  |  | * |  |  |  |  |  |  |  |  | Pathogen | Human blood culture [31] | polymorphic morphology |
| *Cutibacterium avidum* |  | * |  |  |  |  |  |  |  |  |  |  |  | Opportunistic pathogen | Human axillary region or wet sites; involved in endocarditis, and cutaneous infections [32] | It can form biofilms and exhibits antitumor properties, the number of microorganisms increases during puberty [32] |
| *Cutibacterium granulosum* |  |  | * |  |  |  |  |  |  |  |  |  |  | commensal | human skin, especially around sebaceous glands [33] | Secretion of a biofilm matrix degrading enzyme, BmdE [34] |
| *Dietzia maris* |  |  | * |  |  |  | * |  |  |  |  |  |  | opportunistic pathogen | soil; skin, and the intestinal tract of carp [35, 36] in humans isolated from bloodstream infections, artificial hips, bone marrow, and aortitis [36-39] | oil degradation and biosurfactant production [40] |
| *Haemophilus parahaemolyticus* |  |  | * |  |  |  |  |  |  |  |  |  |  | Opportunistic pathogens | commensal of the upper respiratory tract may involve in endocarditis and pharyngitis [41] | associated with acute respiratory distress syndrome and septic shock [41] |
| *Hymenobacter saemangeumensis* |  | * |  |  |  |  |  |  |  |  |  |  |  | Environmental | Sea water [42]. | extremely halophilic red-pink-pigmented bacterium [42] |
| *Ileibacterium valens* |  |  |  |  |  | * |  |  |  |  |  |  |  | commensal | Murine intestinal [43] | association with metabolic health |
| *Kocuria palustris* |  | * |  |  |  | * | * |  |  |  |  |  |  | commensal | cause various infections, particularly in neonates and immunocompromised individuals [44-46]. | formation of complex biofilms with other microorganisms [47, 48]. |
| *Lactobacillus aviarius* |  |  | * |  |  |  |  |  |  |  |  |  |  | Probiotic | Gastrointestinal tract of chicken [49] | good probiotic characteristics [49] |
| *Lactobacillus panis* |  |  |  |  |  |  |  |  | * |  |  |  |  | Probiotic | Long-fermented rye sourdough [50] | Fermentation [50] |
| *Lactobacillus pontis* |  |  |  |  |  | * |  |  |  |  |  |  |  | Probiotic | Fermented milk, sourdough, and broiler chicken gastrointestinal tract [51] | with long chains of slender cells responsible for sourdough fermentation [51] |
| *Leptotrichia hongkongensis* |  |  |  |  |  |  |  |  | * |  |  |  |  | Opportunistic pathogens | oral cavity and blood culture of a patient with metastatic breast carcinoma [52] | A Gram-variable bacillus [52] |
| *Massilia plicata* |  |  |  |  |  |  | * |  |  |  |  |  |  | Environmental | heavy-metal-polluted farm soil [53] | Formation of coiled colonies [53] |
| *Metabacterium polyspora* |  | * |  |  |  |  |  |  |  |  |  |  |  | symbiont | Gastrointestinal tract of guinea pig [54] | Unusual bacterium with the ability to produce multiple endospores [54] |
| *Moraxella atlantae* |  |  |  |  |  | * |  |  |  |  |  |  |  | Rare opportunistic pathogen | Blood, pleural fluid, a dog bite wound [55] | A mesophilic bacterium [55] |
| *Mucispirillum schaedleri* |  |  |  |  |  |  |  |  |  |  | * |  |  | symbiont | a mucus inhabitant and core member of the murine and human gut microbiota [56, 57] | Nitrate reduction and antioxidant activity [56] |
| *Murdochiella asaccharolytica* | * |  |  |  |  |  |  |  |  |  |  |  |  | Pathogen | Human wound specimen [58] | Inability to digest sugar [58] |
| *Mycobacterium tuberculosis* |  |  |  |  |  | * |  |  |  |  |  |  |  | pathogen | Mammalian respiratory system [59] | Biofilm formation is involved in casseous necrosis and cavity formation in lung tissue [59] |
| *Mycoplasma penetrans* | * |  |  |  |  |  |  |  |  |  |  |  |  | pathogen | Human urogenital and respiratory system [60]. | Penetration into human cells and bacterial internalization into the urothelium [60] |
| *Myroides odoratimimus* |  |  | * |  |  |  |  |  |  |  |  |  |  | opportunistic pathogen; symbiont! | soil, fresh and marine water, food, and sewage treatment plants [61]; intestine of flesh flies and lepidopterans [62] | the capacity for self-accumulation and biofilm formation [63]; antagonistic effects on bacteria isolated from human clinical samples, environmental samples, and insect midgut [62] |
| *Myroides phaeus* | * |  |  |  |  |  |  |  |  |  |  |  |  | Opportunistic pathogen | Human saliva [17] and blood [64], medicinal maggots [64] | brown colour colonies on LB agar |
| *Nocardioides furvisabuli* |  |  |  |  |  |  | * |  |  |  |  |  |  | environmental | Black sand from Samyang Beach [65] | No mycelium or spore production [65] |
| *Parabacteroides goldsteinii* |  |  |  |  |  |  |  |  |  |  | * |  |  | commensal | intestines, faeces and blood  [66-69] | a novel next-generation probiotic acting against Helicobacter pylori [67] |
| *Peredibacter starrii* |  |  |  |  |  |  | * |  |  |  |  |  |  | Environmental | Soil [70] | bacteriovorous species that feeds on larger Gram-negative bacteria |
| *Prevotella copri* |  |  | * |  |  |  |  |  |  |  |  |  |  | commensal; Pathogen | The human gut [71] and contributing factor for the development of rheumatoid arthritis [72] | modulating the glucose metabolism and insulin levels [73] |
| *Prevotella melaninogenica* |  |  | * |  |  |  |  |  |  |  |  |  |  | commensal of the upper respiratory tract | oral cavity, throat, tonsils [74]; lungs of people with cystic fibrosis [75] | The formation of biofilm in the oral cavity and favoring the conditions for periodontitis [76, 77] |
| *Prevotella nanceiensis* |  |  |  |  | * |  |  |  |  |  |  |  |  | pathogen | Bronchial fluid, lung abscesses, or blood in human [75] | as a discriminatory species for previous smokers [75] |
| *Prevotella oulorum* | * |  |  |  |  |  |  |  |  |  |  |  |  | pathogen | Subgingival plaque [74] | The formation of biofilm in the oral cavity and favoring the conditions for periodontitis [76, 77]. |
| *Prevotella salivae* |  |  | * |  |  |  |  |  |  |  |  |  |  | pathogen | Oral cavity (saliva) [78] | The cause of chronic periodontitis [78] |
| *Pseudomonas luteola* |  |  | * |  |  |  |  |  |  |  |  |  |  | Opportunistic pathogen | Human and animal clinical specimens [79], rice flour [80] and soil [81] | Production of amylase and lipase [81] |
| *Pseudomonas saudimassiliensis* |  |  |  |  |  |  |  | * |  |  |  |  |  | Environmental | Isolated from air samples in the urban environment of Makkah [82]. | growth at alkaline pH with an optimum at pH ≥10 [82] |
| *Ruminococcus flavefaciens* |  |  | * |  |  |  |  |  |  |  |  |  |  | Commensal | Pony and donkey cecum [83] | cellulose degradation [84] |
| *Slackia piriformis* |  |  |  |  | * |  |  |  |  |  |  |  |  | human gut microbiota | faeces [85] | Important in soybean metabolism in human intestine [86] |
| *Sphingobacterium tabacisoli* | * |  |  |  |  |  |  |  |  |  |  |  |  | Environmental | Tobacco field soil [87] | Growth at 10–35 °C, and pH 6.0–9.0 [87] |
| *Sphingobium xenophagum* |  |  |  |  |  |  |  |  |  | * |  |  |  | Environmental | water resources [88] | phenanthrene degradation [88] |
| *Stackebrandtia cavernae* |  |  | * |  |  |  |  |  |  |  |  |  |  | environmental | a rock from a karst cave [89] | optimum growth at 28–30 °C and pH 7–8 [89] |
| *Stackebrandtia endophytica* |  |  | * |  |  |  |  |  |  |  |  |  |  | Endophytic | *Tripterygium wilfordii* tree stems [90] | Utilizes trehalose, d-galactose, sucrose and xylose as sole carbon sources [90] |
| *Staphylococcus succinus* |  | * | * |  |  |  |  |  |  |  |  |  |  | Commensal | diverse environments, including cheese [91], sausages [92], the skin of various mammals [93, 94], Drosophila [95], Phlebotomus papatasi gut [96], Dominican amber [97]; and human clinical specimens [94] | a plant growth-promoting bacterium [98] |
| *Stenotrophomonas rhizophila* |  | * | * |  | * |  |  |  |  | * |  |  |  | Environmental / non-pathogen | A plant-associated bacterium isolated from the rhizosphere [99] | Protection of plants against bacterial and fungal pathogens [100]; lignocellulose degradation [101]; a harmless alternative for biotechnological applications because unable to growth at human body temperature [102] |
| *Streptococcus mutans* |  |  |  |  |  |  |  |  | * |  |  |  |  | pathogen | Human oral cavity, cause of dental caries [103] | Multispecies biofilms formed on the hard surfaces of the tooth [103]. |
| *Streptococcus parauberis* |  | * |  |  |  |  |  |  | * |  |  |  |  | pathogen | Mammary gland, [104]; Fish [105]; Human hand infection associated with seafood handling [106]. | pyogenic streptococcus [104] |
| *Streptococcus salivarius* |  |  | * |  | * |  |  |  | * |  |  | * | * | Probiotic; Opportunistic pathogens | Oral cavity digestive tract, and brain [107-109]. | generally with anti-inflammatory properties [18, 110] |
| *Thermomonospora umbrina* |  |  | * |  |  |  |  |  |  |  |  |  |  | Environmental | the genus have been isolated from springs, water holes, mud holes, and soil [111] | Convert plant residues into compost [112, 113] |
| *Thermus scotoductus* |  |  |  |  | * |  |  |  |  |  |  |  |  | Environmental | Warm spring water [114] | A thermophilic Bacterium |
| *Weissella viridescens* |  |  | * |  |  |  |  |  |  |  |  |  |  | Opportunistic pathogens | Feces of children with celiac disease and blood from patients with bacteremia [115, 116]; cured meat [117]. | Prevention of *C. acnes* growth and reduction its adhesion to keratinocytes [118] |
| *Zoogloea oryzae* |  |  |  |  |  | * |  |  |  |  |  |  |  | Environmental | Rice paddy soil [119] | Nitrogen-fixation [119] |

**Abbreviation**

**AK:** Akanlo

**BU:** Bukan

**SA:** Sarab

**TE:** Tello

**F:** Female

**M:** Male

**MF:** Male and Female

**NI:** *Nosopsyllus iranus iranus*

**PI:** *Pulex irritans*

**XB:** *Xenopsylla buxtoni*

**XN:** *Xenopsylla nuttalli*

**CRBS**: *Ctenophthalmus rettigi smiti* both male and female from Sarab

**NIFS**: *Nosopsyllus iranus iranus* female from Sarab

**NIMA**: *Nosopsyllus iranus iranus* male from Akanlo

**PIFA**: *Pulex irritans* female from Akanlo

**PIMA**: *Pulex irritans* male from Akanlo

**XBFA**: *Xenopsylla buxtoni* female from Akanlo

**XBFB**: *Xenopsylla buxtoni* female from Bukan

**XBFS**: *Xenopsylla buxtoni* female from Sarab

**XBFT**: *Xenopsylla buxtoni* female from Tello

**XBMA**: *Xenopsylla buxtoni* male from Akanlo

**XBMB**: *Xenopsylla buxtoni* male from Bukan

**XNFS**: *Xenopsylla nuttalli* female from Sarab

**XNMS**: *Xenopsylla nuttalli* male from Sarab

**CRB:** *Ctenophthalmus rettigi smiti*

**References**1. Vaz-Moreira I, Novo A, Hantsis-Zacharov E *et al.* Acinetobacter rudis sp. Nov., isolated from raw milk and raw wastewater. *International journal of systematic and evolutionary microbiology*. 2011;**61**:2837-43 <https://doi.org/10.1099/ijs.0.027045-0>

2. Munsch-Alatossava P, Alatossava T. Phenotypic characterization of raw milk-associated psychrotrophic bacteria. *Microbiological research*. 2006;**161**:334-46 <https://doi.org/10.1016/j.micres.2005.12.004>

3. Heinle CE, Junqueira ACM, Uchida A *et al.* Complete genome sequence of lelliottia nimipressuralis type strain sgair0187, isolated from tropical air collected in singapore. *Genome announcements*. 2018;**6** <https://doi.org/10.1128/genomeA.00231-18>

4. Dortet L, Legrand P, Soussy CJ *et al.* Bacterial identification, clinical significance, and antimicrobial susceptibilities of acinetobacter ursingii and acinetobacter schindleri, two frequently misidentified opportunistic pathogens. *Journal of clinical microbiology*. 2006;**44**:4471-8 <https://doi.org/10.1128/jcm.01535-06>

5. Chiu CH, Lee YT, Wang YC *et al.* A retrospective study of the incidence, clinical characteristics, identification, and antimicrobial susceptibility of bacteremic isolates of acinetobacter ursingii. *BMC Infect Dis*. 2015;**15**:400 <https://doi.org/10.1186/s12879-015-1145-z>

6. Itoh T, Kudo T, Oyaizu H *et al.* Two new species in the genus actinomadura: A. Glomerata sp. Nov., and a. Longicatena sp. Nov. *J Actinomycetologica*. 1995;**9**:164-77

7. Renvoise A, Raoult D, Roux V. Actinomyces massiliensis sp. Nov., isolated from a patient blood culture. *International journal of systematic and evolutionary microbiology*. 2009;**59**:540-4 <https://doi.org/10.1099/ijs.0.001503-0>

8. Stingu CS, Borgmann T, Rodloff AC *et al.* Rapid identification of oral actinomyces species cultivated from subgingival biofilm by maldi-tof-ms. *Journal of oral microbiology*. 2015;**7**:26110 <https://doi.org/10.3402/jom.v7.26110>

9. Cobo F, Navarro-Marí JM. First description of anaerococcus octavius as cause of bacteremia. *Anaerobe*. 2020;**61**:102130 <https://doi.org/10.1016/j.anaerobe.2019.102130>

10. Ezaki T, Kawamura Y, Li N *et al.* Proposal of the genera anaerococcus gen. Nov., peptoniphilus gen. Nov. And gallicola gen. Nov. For members of the genus peptostreptococcus. *International journal of systematic and evolutionary microbiology*. 2001;**51**:1521-28 <https://doi.org/10.1099/00207713-51-4-1521>

11. Cao J, Yu Z, Liu W *et al.* Probiotic characteristics of bacillus coagulans and associated implications for human health and diseases. *Journal of Functional Foods*. 2020;**64**:103643 <https://doi.org/https://doi.org/10.1016/j.jff.2019.103643>

12. Yohandini H, Julinar, Muharni. Isolation and phylogenetic analysis of thermophile community within tanjung sakti hot spring, south sumatera, indonesia. *HAYATI Journal of Biosciences*. 2015;**22**:143-48 <https://doi.org/https://doi.org/10.1016/j.hjb.2015.10.006>

13. Flint S, Gonzaga ZJ, Good J *et al.* Bacillus thermoamylovorans – a new threat to the dairy industry – a review. *International Dairy Journal*. 2017;**65**:38-43 <https://doi.org/https://doi.org/10.1016/j.idairyj.2016.10.002>

14. Lücking G, Stoeckel M, Atamer Z *et al.* Characterization of aerobic spore-forming bacteria associated with industrial dairy processing environments and product spoilage. *International journal of food microbiology*. 2013;**166**:270-9 <https://doi.org/10.1016/j.ijfoodmicro.2013.07.004>

15. Kitahara M, Sakamoto M, Ike M *et al.* Bacteroides plebeius sp nov and bacteroides coprocola sp nov., isolated from human faeces. *International journal of systematic and evolutionary microbiology*. 2005;**55**:2143-47 <https://doi.org/10.1099/ijs.0.63788-0>

16. Li M, Li G, Zhu L *et al.* Isolation and characterization of an agaro-oligosaccharide (ao)-hydrolyzing bacterium from the gut microflora of chinese individuals. *PLOS ONE*. 2014;**9**:e91106 <https://doi.org/10.1371/journal.pone.0091106>

17. Yang J, Pu J, Lu S *et al.* Species-level analysis of human gut microbiota with metataxonomics. *J Frontiers in microbiology*. 2020;**11**:2029

18. Macfarlane S, Macfarlane GT. Composition and metabolic activities of bacterial biofilms colonizing food residues in the human gut. *Applied and environmental microbiology*. 2006;**72**:6204-11 <https://doi.org/10.1128/aem.00754-06>

19. Praet J, Meeus I, Cnockaert M *et al.* Bifidobacterium commune sp. Nov. Isolated from the bumble bee gut. *Antonie van Leeuwenhoek*. 2015;**107**:1307-13 <https://doi.org/10.1007/s10482-015-0425-3>

20. Katı H, İnce İ A, Demir İ *et al.* Brevibacterium pityocampae sp. Nov., isolated from caterpillars of thaumetopoea pityocampa (lepidoptera, thaumetopoeidae). *International journal of systematic and evolutionary microbiology*. 2010;**60**:312-16 <https://doi.org/10.1099/ijs.0.006692-0>

21. Tanner A, Maiden MF, Macuch PJ *et al.* Microbiota of health, gingivitis, and initial periodontitis. *Journal of clinical periodontology*. 1998;**25**:85-98 <https://doi.org/10.1111/j.1600-051x.1998.tb02414.x>

22. Johnson CC, Reinhardt JF, Edelstein MA *et al.* Bacteroides gracilis, an important anaerobic bacterial pathogen. *Journal of clinical microbiology*. 1985;**22**:799-802 <https://doi.org/10.1128/jcm.22.5.799-802.1985>

23. Gunther NWt, Chen CY. The biofilm forming potential of bacterial species in the genus campylobacter. *Food microbiology*. 2009;**26**:44-51 <https://doi.org/10.1016/j.fm.2008.07.012>

24. *Principles and practice of pediatric infectious diseases*: Elsevier Health Sciences, March 10, 2022.

25. Ozdemir S, Aydogan O, Koksal Cakirlar F. Biofilm formation and antimicrobial susceptibility of non-diphtheria corynebacterium strains isolated from blood cultures: First report from turkey. *Medeniyet medical journal*. 2021;**36**:123-29 <https://doi.org/10.5222/mmj.2021.60252>

26. Rudresh SM, Ravi GS, Alex AM *et al.* Non diphtheritic corynebacteria: An emerging nosocomial pathogen in skin and soft tissue infection. *Journal of clinical and diagnostic research : JCDR*. 2015;**9**:Dc19-21 <https://doi.org/10.7860/jcdr/2015/15580.6977>

27. Yassin AF, Steiner U, Ludwig W. Corynebacterium appendicis sp. Nov. *International journal of systematic and evolutionary microbiology*. 2002;**52**:1165-69 <https://doi.org/10.1099/00207713-52-4-1165>

28. Funke G, Efstratiou A, Kuklinska D *et al.* Corynebacterium imitans sp. Nov. Isolated from patients with suspected diphtheria. *Journal of clinical microbiology*. 1997;**35**:1978-83 <https://doi.org/10.1128/jcm.35.8.1978-1983.1997>

29. Dinakaran V, Mandape SN, Shuba K *et al.* Identification of specific oral and gut pathogens in full thickness colon of colitis patients: Implications for colon motility. *Frontiers in microbiology*. 2018;**9**:3220 <https://doi.org/10.3389/fmicb.2018.03220>

30. Kim JH, Bang IH, Noh YJ *et al.* Metabolites produced by the oral commensal bacterium corynebacterium durum extend the lifespan of caenorhabditis elegans via sir-2.1 overexpression. *J International Journal of Molecular Sciences*. 2020;**21**:2212

31. Yassin AF, Inglis TJ, Hupfer H *et al.* Cruoricaptor ignavus gen. Nov., sp. Nov., a novel bacterium of the family flavobacteriaceae isolated from blood culture of a man with bacteraemia. *Systematic and applied microbiology*. 2012;**35**:421-6 <https://doi.org/10.1016/j.syapm.2012.08.003>

32. Corvec S. Clinical and biological features of cutibacterium (formerly propionibacterium) avidum, an underrecognized microorganism. *Clinical microbiology reviews*. 2018;**31** <https://doi.org/10.1128/cmr.00064-17>

33. McGinley KJ, Webster GF, Leyden JJ. Regional variations of cutaneous propionibacteria. *Applied and environmental microbiology*. 1978;**35**:62-6 <https://doi.org/10.1128/aem.35.1.62-66.1978>

34. Bronnec V, Eilers H, Jahns AC *et al.* Propionibacterium (cutibacterium) granulosum extracellular dnase bmde targeting propionibacterium (cutibacterium) acnes biofilm matrix, a novel inter-species competition mechanism. *Frontiers in cellular and infection microbiology*. 2021;**11**:809792 <https://doi.org/10.3389/fcimb.2021.809792>

35. Nesterenko O, Nogina T, Kasumova S *et al.* Rhodococcus luteus nom. Nov. And rhodococcus maris nom. Nov. *J International Journal of Systematic Evolutionary Microbiology*. 1982;**32**:1-14

36. Reyes G, Navarro JL, Gamallo C *et al.* Type a aortic dissection associated with dietzia maris. *Interactive cardiovascular and thoracic surgery*. 2006;**5**:666-8 <https://doi.org/10.1510/icvts.2006.135640>

37. Pidoux O, Argenson JN, Jacomo V *et al.* Molecular identification of a dietzia maris hip prosthesis infection isolate. *Journal of clinical microbiology*. 2001;**39**:2634-6 <https://doi.org/10.1128/jcm.39.7.2634-2636.2001>

38. Azcona-Gutiérrez J, Arponen S, Sarriá C *et al.* Isolation of dietzia maris from bone marrow in an immunocompetent patient. *Clinical Microbiology Newsletter*. 2011;**33**:52-4 <https://doi.org/10.1016/j.clinmicnews.2011.03.002>

39. Bemer-Melchior P, Haloun A, Riegel P *et al.* Bacteremia due to dietzia maris in an immunocompromised patient. *Clinical Infectious Diseases*. 1999;**29**:1338-40 <https://doi.org/10.1086/313490> %J Clinical Infectious Diseases

40. Wang W, Cai B, Shao Z. Oil degradation and biosurfactant production by the deep sea bacterium dietzia maris as-13-3. 2014;**5** <https://doi.org/10.3389/fmicb.2014.00711>

41. Le Floch AS, Cassir N, Hraiech S *et al.* Haemophilus parahaemolyticus septic shock after aspiration pneumonia, france. *Emerging infectious diseases*. 2013;**19**:1694-5 <https://doi.org/10.3201/eid1910.130608>

42. Kang JY, Chun J, Choi A *et al.* Hymenobacter koreensis sp. Nov. And hymenobacter saemangeumensis sp. Nov., isolated from estuarine water. *International journal of systematic and evolutionary microbiology*. 2013;**63**:4568-73 <https://doi.org/10.1099/ijs.0.051870-0>

43. Cox LM, Sohn J, Tyrrell KL *et al.* Description of two novel members of the family erysipelotrichaceae: Ileibacterium valens gen. Nov., sp. Nov. And dubosiella newyorkensis, gen. Nov., sp. Nov., from the murine intestine, and emendation to the description of faecalibaculum rodentium. *International journal of systematic and evolutionary microbiology*. 2017;**67**:1247-54 <https://doi.org/10.1099/ijsem.0.001793>

44. Stackebrandt E, Koch C, Gvozdiak O *et al.* Taxonomic dissection of the genus micrococcus: Kocuria gen. Nov., nesterenkonia gen. Nov., kytococcus gen. Nov., dermacoccus gen. Nov., and micrococcus cohn 1872 gen. Emend. *International journal of systematic bacteriology*. 1995;**45**:682-92 <https://doi.org/10.1099/00207713-45-4-682>

45. Purty S, Saranathan R, Prashanth K *et al.* The expanding spectrum of human infections caused by kocuria species: A case report and literature review. *Emerging microbes & infections*. 2013;**2**:e71 <https://doi.org/10.1038/emi.2013.71>

46. Lee MK, Choi SH, Ryu DW. Descending necrotizing mediastinitis caused by kocuria rosea: A case report. *BMC Infectious Diseases*. 2013;**13**:475 <https://doi.org/10.1186/1471-2334-13-475>

47. Kandi V, Palange P, Vaish R *et al.* Emerging bacterial infection: Identification and clinical significance of kocuria species. *Cureus*. 2016;**8**:e731 <https://doi.org/10.7759/cureus.731>

48. Dotis J, Printza N, Stabouli S *et al.* Kocuria species peritonitis: Although rare, we have to care. *Peritoneal dialysis international : journal of the International Society for Peritoneal Dialysis*. 2015;**35**:26-30 <https://doi.org/10.3747/pdi.2013.00138>

49. Wang L, Fang M, Hu Y *et al.* Characterization of the most abundant lactobacillus species in chicken gastrointestinal tract and potential use as probiotics for genetic engineering. *Acta biochimica et biophysica Sinica*. 2014;**46**:612-9 <https://doi.org/10.1093/abbs/gmu037>

50. Wiese BG, Strohmar W, Rainey FA *et al.* Lactobacillus panis sp. Nov., from sourdough with a long fermentation period. *International journal of systematic bacteriology*. 1996;**46**:449-53 <https://doi.org/10.1099/00207713-46-2-449>

51. Vogel RF, Böcker G, Stolz P *et al.* Identification of lactobacilli from sourdough and description of lactobacillus pontis sp. Nov. *International journal of systematic bacteriology*. 1994;**44**:223-9 <https://doi.org/10.1099/00207713-44-2-223>

52. Woo PC, Wong SS, Teng JL *et al.* Leptotrichia hongkongensis sp. Nov., a novel leptotrichia species with the oral cavity as its natural reservoir. *Journal of Zhejiang University Science B*. 2010;**11**:391-401 <https://doi.org/10.1631/jzus.B1000056>

53. Zhang YQ, Li WJ, Zhang KY *et al.* Massilia dura sp. Nov., massilia albidiflava sp. Nov., massilia plicata sp. Nov. And massilia lutea sp. Nov., isolated from soils in china. *International journal of systematic and evolutionary microbiology*. 2006;**56**:459-63 <https://doi.org/10.1099/ijs.0.64083-0>

54. Chatton E, Pérard C. *Schizophytes du caecum du cobaye*, vol. 74, Compendes Rendus Hebdomadaires Societe de Biologie (Paris): Maretheux, 1913.

55. De Baere T, Muylaert A, Everaert E *et al.* Bacteremia due to moraxella atlantae in a cancer patient. *Journal of clinical microbiology*. 2002;**40**:2693-5 <https://doi.org/10.1128/jcm.40.7.2693-2695.2002>

56. Loy A, Pfann C, Steinberger M *et al.* Lifestyle and horizontal gene transfer-mediated evolution of mucispirillum schaedleri, a core member of the murine gut microbiota. *mSystems*. 2017;**2** <https://doi.org/10.1128/mSystems.00171-16>

57. Herp S, Durai Raj AC, Salvado Silva M *et al.* The human symbiont mucispirillum schaedleri: Causality in health and disease. *Medical microbiology and immunology*. 2021;**210**:173-79 <https://doi.org/10.1007/s00430-021-00702-9>

58. Ulger-Toprak N, Liu C, Summanen PH *et al.* Murdochiella asaccharolytica gen. Nov., sp. Nov., a gram-stain-positive, anaerobic coccus isolated from human wound specimens. *International journal of systematic and evolutionary microbiology*. 2010;**60**:1013-16 <https://doi.org/10.1099/ijs.0.015909-0>

59. Borgdorff MW, Nagelkerke NJ, Dye C *et al.* Gender and tuberculosis: A comparison of prevalence surveys with notification data to explore sex differences in case detection. *The international journal of tuberculosis and lung disease : the official journal of the International Union against Tuberculosis and Lung Disease*. 2000;**4**:123-32

60. Lo SMJ, McElhaney RN, Finch LR, Baseman JB. Mycoplasmas and aids. Mycoplasmas: Molecular biology and pathogenesis. *Washington, DC: American Society for Microbiology*. 1992:525–45

61. Holmes B, Snell JJ, Lapage SP. Flavobacterium odoratum: A species resistant to a wide range of antimicrobial agents. *Journal of clinical pathology*. 1979;**32**:73-7 <https://doi.org/10.1136/jcp.32.1.73>

62. Dharne MS, Gupta AK, Rangrez AY *et al.* Antibacterial activities of multi drug resistant myroides odoratimimus bacteria isolated from adult flesh flies (diptera: Sarcophagidae) are independent of metallo beta-lactamase gene. *Brazilian journal of microbiology : [publication of the Brazilian Society for Microbiology]*. 2008;**39**:397-404 <https://doi.org/10.1590/s1517-838220080002000035>

63. Vancanneyt M, Segers P, Torck U *et al.* Reclassification of flavobacterium odoratum (stutzer 1929) strains to a new genus, myroides, as myroides odoratus comb. Nov. And myroides odoratimimus sp. Nov. *International journal of systematic bacteriology*. 1996;**46** <https://doi.org/10.1099/00207713-46-4-926>

64. Pérez-Lazo G, Morales-Moreno A, Soto-Febres F *et al.* First report of myroides phaeus bacteraemia identified by polymerase chain reaction and genetic sequencing. *IDCases*. 2020;**19**:e00695 <https://doi.org/10.1016/j.idcr.2020.e00695>

65. Lee SD. Nocardioides furvisabuli sp. Nov., isolated from black sand. *International journal of systematic and evolutionary microbiology*. 2007;**57**:35-39 <https://doi.org/10.1099/ijs.0.64444-0>

66. Song Y, Liu C, Lee J *et al.* “Bacteroides goldsteinii sp. Nov.” isolated from clinical specimens of human intestinal origin. *J Journal of clinical microbiology*. 2005;**43**:4522-27

67. Lai CH, Lin TL, Huang MZ *et al.* Gut commensal parabacteroides goldsteinii mts01 alters gut microbiota composition and reduces cholesterol to mitigate helicobacter pylori-induced pathogenesis. *Frontiers in immunology*. 2022;**13**:916848 <https://doi.org/10.3389/fimmu.2022.916848>

68. Awadel-Kariem FM, Patel P, Kapoor J *et al.* First report of parabacteroides goldsteinii bacteraemia in a patient with complicated intra-abdominal infection. *Anaerobe*. 2010;**16**:223-5 <https://doi.org/10.1016/j.anaerobe.2010.01.001>

69. Krogh TJ, Agergaard CN, Møller-Jensen J *et al.* Draft genome sequence of parabacteroides goldsteinii with putative novel metallo-β-lactamases isolated from a blood culture from a human patient. *Genome announcements*. 2015;**3** <https://doi.org/10.1128/genomeA.00937-15>

70. Seideler RJ, Mandel M, Baptist JN. Molecular heterogeneity of the bdellovibrios: Evidence of two new species. *Journal of bacteriology*. 1972;**109**:209-17 <https://doi.org/10.1128/jb.109.1.209-217.1972>

71. Tett A, Huang KD, Asnicar F *et al.* The prevotella copri complex comprises four distinct clades underrepresented in westernized populations. *Cell host & microbe*. 2019;**26**:666-79.e7 <https://doi.org/10.1016/j.chom.2019.08.018>

72. Moreno J. Prevotella copri and the microbial pathogenesis of rheumatoid arthritis. *Reumatologia clinica*. 2015;**11**:61-3 <https://doi.org/10.1016/j.reuma.2014.11.001>

73. Kovatcheva-Datchary P, Nilsson A, Akrami R *et al.* Dietary fiber-induced improvement in glucose metabolism is associated with increased abundance of prevotella. *Cell metabolism*. 2015;**22**:971-82 <https://doi.org/10.1016/j.cmet.2015.10.001>

74. Shah HN, Collins MD, Watabe J *et al.* Bacteroides oulorum sp. Nov., a nonpigmented saccharolytic species from the oral cavity. *J International Journal of Systematic*

*Evolutionary Microbiology*. 1985;**35**:193-97

75. Lamoureux C, Guilloux CA, Courteboeuf E *et al.* Prevotella melaninogenica, a sentinel species of antibiotic resistance in cystic fibrosis respiratory niche? *Microorganisms*. 2021;**9** <https://doi.org/10.3390/microorganisms9061275>

76. Albaghdadi SZ, Altaher JB, Drobiova H *et al.* In vitro characterization of biofilm formation in prevotella species. *J Frontiers in Oral Health*. 2021;**2**:724194

77. Bamashmous S, Kotsakis GA, Kerns KA *et al.* Human variation in gingival inflammation. *Proceedings of the National Academy of Sciences of the United States of America*. 2021;**118** <https://doi.org/10.1073/pnas.2012578118>

78. Sakamoto M, Suzuki M, Huang Y *et al.* Prevotella shahii sp. Nov. And prevotella salivae sp. Nov., isolated from the human oral cavity. *International journal of systematic and evolutionary microbiology*. 2004;**54**:877-83 <https://doi.org/10.1099/ijs.0.02876-0>

79. Ali MA, Aljanaby AAJ First case report of pseudomonas luteola isolated from urinary tract infection in babylon city, iraq. *E3S Web of Conferences*: EDP Sciences.

80. Kodama K, Kimura N, Komagata K. Two new species of pseudomonas: P. Oryzihabitans isolated from rice paddy and clinical specimens and p. Luteola isolated from clinical specimens. *J International Journal of Systematic*

*Evolutionary Microbiology*. 1985;**35**:467-74

81. Khannous L, Jrad M, Dammak M *et al.* Isolation of a novel amylase and lipase-producing pseudomonas luteola strain: Study of amylase production conditions. *Lipids in health and disease*. 2014;**13**:9 <https://doi.org/10.1186/1476-511x-13-9>

82. Azhar EI, Papadioti A, Bibi F *et al.* 'Pseudomonas saudimassiliensis' sp. Nov. A new bacterial species isolated from air samples in the urban environment of makkah, saudi arabia. *New microbes and new infections*. 2017;**16**:43-44 <https://doi.org/10.1016/j.nmni.2016.12.021>

83. Julliand V, de Vaux A, Millet L *et al.* Identification of ruminococcus flavefaciens as the predominant cellulolytic bacterial species of the equine cecum. *Applied and environmental microbiology*. 1999;**65**:3738-41 <https://doi.org/10.1128/aem.65.8.3738-3741.1999>

84. Maki M, Leung KT, Qin W. The prospects of cellulase-producing bacteria for the bioconversion of lignocellulosic biomass. *International journal of biological sciences*. 2009;**5**:500-16 <https://doi.org/10.7150/ijbs.5.500>

85. Nagai F, Watanabe Y, Morotomi M. Slackia piriformis sp. Nov. And collinsella tanakaei sp. Nov., new members of the family coriobacteriaceae, isolated from human faeces. *International journal of systematic and evolutionary microbiology*. 2010;**60**:2639-46 <https://doi.org/10.1099/ijs.0.017533-0>

86. Yuan JP, Wang JH, Liu X. Metabolism of dietary soy isoflavones to equol by human intestinal microflora--implications for health. *Molecular nutrition & food research*. 2007;**51**:765-81 <https://doi.org/10.1002/mnfr.200600262>

87. Zhou XK, Li QQ, Mo MH *et al.* Sphingobacterium tabacisoli sp. Nov., isolated from a tobacco field soil sample. *International journal of systematic and evolutionary microbiology*. 2017;**67**:4808-13 <https://doi.org/10.1099/ijsem.0.002381>

88. Iwabuchi T. Phenanthrene-degrading sphingobium xenophagum are widely distributed in the western pacific ocean. *Canadian journal of microbiology*. 2022;**68**:315-28 <https://doi.org/10.1139/cjm-2021-0177>

89. Zhang WQ, Li YQ, Liu L *et al.* Stackebrandtia cavernae sp. Nov., a novel actinobacterium isolated from a karst cave sample. *International journal of systematic and evolutionary microbiology*. 2016;**66**:1206-11 <https://doi.org/10.1099/ijsem.0.000859>

90. Xiong ZJ, Miao CP, Zheng YK *et al.* Stackebrandtia endophytica sp. Nov., an actinobacterium isolated from tripterygium wilfordii. *International journal of systematic and evolutionary microbiology*. 2015;**65**:1709-13 <https://doi.org/10.1099/ijs.0.000166>

91. Place RB, Hiestand D, Burri S *et al.* Staphylococcus succinus subsp. Casei subsp. Nov., a dominant isolate from a surface ripened cheese. *Systematic and applied microbiology*. 2002;**25**:353-9 <https://doi.org/10.1078/0723-2020-00130>

92. Corbière Morot-Bizot S, Leroy S, Talon R. Staphylococcal community of a small unit manufacturing traditional dry fermented sausages. *International journal of food microbiology*. 2006;**108**:210-7 <https://doi.org/10.1016/j.ijfoodmicro.2005.12.006>

93. Hauschild T, Sliżewski P, Masiewicz P. Species distribution of staphylococci from small wild mammals. *Systematic and applied microbiology*. 2010;**33**:457-60 <https://doi.org/10.1016/j.syapm.2010.08.007>

94. Nováková D, Sedláček I, Pantůček R *et al.* Staphylococcus equorum and staphylococcus succinus isolated from human clinical specimens. *Journal of medical microbiology*. 2006;**55**:523-28 <https://doi.org/10.1099/jmm.0.46246-0>

95. Singh K, Zulkifli M, Prasad NG. Identification and characterization of novel natural pathogen of drosophila melanogaster isolated from wild captured drosophila spp. *Microbes and infection*. 2016;**18**:813-21 <https://doi.org/10.1016/j.micinf.2016.07.008>

96. Maleki-Ravasan N, Oshaghi MA, Afshar D *et al.* Aerobic bacterial flora of biotic and abiotic compartments of a hyperendemic zoonotic cutaneous leishmaniasis (zcl) focus. *Parasites & vectors*. 2015;**8**:63 <https://doi.org/10.1186/s13071-014-0517-3>

97. Lambert LH, Cox T, Mitchell K *et al.* Staphylococcus succinus sp. Nov., isolated from dominican amber. *International journal of systematic bacteriology*. 1998;**48 Pt 2**:511-8 <https://doi.org/10.1099/00207713-48-2-511>

98. Shirmohammadi E, Alikhani HA, Pourbabaee AA *et al.* Effect of super strains of bacillus and staphylococcus isolated from dryland farming on quantitative and qualitative indices of wheat under stress condition. *JOURNAL OF WATER AND SOIL (AGRICULTURAL SCIENCES AND TECHNOLOGY)*. 2020;**34**

99. Raio A, Brilli F, Neri L *et al.* Stenotrophomonas rhizophila ep2.2 inhibits growth of botrytis cinerea through the emission of volatile organic compounds, restricts leaf infection and primes defense genes. *Frontiers in plant science*. 2023;**14**:1235669 <https://doi.org/10.3389/fpls.2023.1235669>

100. Berg G, Egamberdieva D, Lugtenberg B *et al.* Symbiotic plant–microbe interactions: Stress protection, plant growth promotion, and biocontrol by stenotrophomonas. *J Symbioses stress: joint ventures in biology*. 2010:445-60

101. de Lima Brossi MJ, Jiménez DJ, Cortes-Tolalpa L *et al.* Soil-derived microbial consortia enriched with different plant biomass reveal distinct players acting in lignocellulose degradation. *Microbial ecology*. 2016;**71**:616-27 <https://doi.org/10.1007/s00248-015-0683-7>

102. Berg G, Martinez JL. Friends or foes: Can we make a distinction between beneficial and harmful strains of the stenotrophomonas maltophilia complex? *Frontiers in microbiology*. 2015;**6**:241 <https://doi.org/10.3389/fmicb.2015.00241>

103. Zhang Q, Ma Q, Wang Y *et al.* Molecular mechanisms of inhibiting glucosyltransferases for biofilm formation in streptococcus mutans. *International Journal of Oral Science*. 2021;**13**:30 <https://doi.org/10.1038/s41368-021-00137-1>

104. Williams AM, Collins MD. Molecular taxonomic studies on streptococcus uberis types i and ii. Description of streptococcus parauberis sp. Nov. *The Journal of applied bacteriology*. 1990;**68**:485-90 <https://doi.org/10.1111/j.1365-2672.1990.tb02900.x>

105. Woo SH, Park SI. Streptococcous parauberis infection in starry flounder, platichthys stellatus: Characterization of innate immune responses following experimental infection. *Fish & shellfish immunology*. 2013;**35**:413-20 <https://doi.org/10.1016/j.fsi.2013.04.047>

106. Huan S, Tan JSW, Chin AYH. Streptococcus parauberis infection of the hand. *The Journal of hand surgery, European volume*. 2021;**46**:83-84 <https://doi.org/10.1177/1753193420938504>

107. Hakalehto E, Vilpponen-Salmela T, Kinnunen K *et al.* Lactic acid bacteria enriched from human gastric biopsies. *ISRN gastroenterology*. 2011;**2011**:109183 <https://doi.org/10.5402/2011/109183>

108. Wang M, Ahrné S, Jeppsson B *et al.* Comparison of bacterial diversity along the human intestinal tract by direct cloning and sequencing of 16s rrna genes. *FEMS microbiology ecology*. 2005;**54**:219-31 <https://doi.org/10.1016/j.femsec.2005.03.012>

109. MacDonald KW, Chanyi RM, Macklaim JM *et al.* Streptococcus salivarius inhibits immune activation by periodontal disease pathogens. *BMC Oral Health*. 2021;**21**:245 <https://doi.org/10.1186/s12903-021-01606-z>

110. Kaci G, Goudercourt D, Dennin V *et al.* Anti-inflammatory properties of streptococcus salivarius, a commensal bacterium of the oral cavity and digestive tract. *Applied and environmental microbiology*. 2014;**80**:928-34 <https://doi.org/10.1128/aem.03133-13>

111. Dworkin M. *The prokaryotes: Vol. 3: Archaea. Bacteria: Firmicutes, actinomycetes*: Springer Science & Business Media, 2006.

112. Fergus C. Thermophilic and thermotolerant molds and actinomycetes of mushroom compost during peak heating. *J Mycologia*. 1964;**56**:267-84

113. Stutzenberger FJ. Cellulase production by thermomonospora curvata isolated from municipal solid waste compost. *Applied microbiology*. 1971;**22**:147-52 <https://doi.org/10.1128/am.22.2.147-152.1971>

114. Kristjánsson JK, Hjörleifsdóttir S, Marteinsson VT *et al.* Thermus scotoductus, sp. Nov., a pigment-producing thermophilic bacterium from hot tap water in iceland and including thermus sp. X-1. *Systematic and applied microbiology*. 1994;**17**:44-50 <https://doi.org/https://doi.org/10.1016/S0723-2020(11)80030-5>

115. Kulwichit W, Nilgate S, Chatsuwan T *et al.* Accuracies of leuconostoc phenotypic identification: A comparison of api systems and conventional phenotypic assays. *BMC Infect Dis*. 2007;**7**:69 <https://doi.org/10.1186/1471-2334-7-69>

116. Sanz Y, Sánchez E, Marzotto M *et al.* Differences in faecal bacterial communities in coeliac and healthy children as detected by pcr and denaturing gradient gel electrophoresis. *FEMS immunology and medical microbiology*. 2007;**51**:562-8 <https://doi.org/10.1111/j.1574-695X.2007.00337.x>

117. Niven Jr C, Evans JB. Lactobacillus viridescens nov. Spec., a heterofermentative species that produces a green discoloration of cured meat pigments. *Journal of bacteriology*. 1957;**73**:758-59

118. Espinoza-Monje M, Campos J, Alvarez Villamil E *et al.* Characterization of weissella viridescens uco-smc3 as a potential probiotic for the skin: Its beneficial role in the pathogenesis of acne vulgaris. *Microorganisms*. 2021;**9** <https://doi.org/10.3390/microorganisms9071486>

119. Xie CH, Yokota A. Zoogloea oryzae sp. Nov., a nitrogen-fixing bacterium isolated from rice paddy soil, and reclassification of the strain atcc 19623 as crabtreella saccharophila gen. Nov., sp. Nov. *International journal of systematic and evolutionary microbiology*. 2006;**56**:619-24 <https://doi.org/10.1099/ijs.0.63755-0>
